# Resolving three-dimensional wind velocity fields with sequential wind-Doppler LiDAR for wind energy in the complex terrain - Gotthard Pass, Switzerland

**DOI:** 10.12688/openreseurope.19095.1

**Published:** 2025-01-15

**Authors:** Brandon van Schaik, Hendrik Huwald, Michael Lehning

**Affiliations:** 1 Laboratory of Cryospheric Sciences, Environmental Engineering Institute, Ecole polytechnique federale de Lausanne (EPFL), Sion, Valais, 1950, Switzerland; 2 Snow Processes, Swiss Institute for Snow and Avalanche Research (SLF), Davos, Graubünden, 7260, Switzerland

**Keywords:** wind, energy, complex terrain, lidar, wind turbine, shear, turbulence, turbulence kinetic energy, wind turbine efficiency, wind channeling, stratification, atmosphere, Gotthard, Switzerland

## Abstract

**Background:**

Understanding the effects of the complex terrain on wind turbines in alpine regions requires high-resolution computational modelling accompanied by detailed wind observations. In technologically advanced measurement campaigns, often multiple synchronised wind-Doppler LiDARs are deployed to overcome the limitation of these instruments to only measure line-of-sight velocity.

**Methods:**

In this work, a novel deployment method, a sequential wind-Doppler LiDAR deployment is introduced. We present the example of a field campaign on the Gotthard Pass, a narrow north-south permutated high-mountain pass in the central Swiss Alps. We propose a matching algorithm that can robustly group wind profiles, enabling comparable scientific detail to study turbine efficiency as in synchronised triple LiDAR campaigns, whilst only requiring a single LiDAR instrument to be deployed.

**Results:**

In the three-month study period in the summer of 2023, we successfully used turbulence kinetic energy, wind shear and veer, as well as wind channelling to explain turbine power production discrepancies observed in the five turbines erected on a mountain pass.

## Plain Language Summary

We have developed a new method of using an existing measurement device to improve the information we can gather about the changes in the wind as they pass over a mountain pass. Five wind turbines were constructed in 2020 on top of a prominent Swiss mountain pass in the Gotthard Wind Park. It is known that these mountain winds are much stronger due to an effect called wind channelling, allowing the wind turbines to generate more energy. With our new measurement method, we can visualise the airflow through the wind park with just one sensor by applying an algorithm that can identify similar wind conditions at different times. Our measurement campaign during the Summer of 2023 allowed us to study several different flow patterns and identify some complex flow structures that affect the efficiency of the wind turbine. We show for each turbine, specifically for Northerly and Southerly winds, how efficient their energy production is, and explain with our detailed wind flow measurements, how these can arise due to the surrounding mountains.

## Introduction

Exact and reliable measurements of the three-dimensional wind are paramount for wind energy potential calculations for the design of wind farms. Wind measurements in complex terrain are much more challenging than over flat terrain due to the increased complexity of wind patterns both horizontally and vertically. The complex terrain, in a wind energy assessment (WEA) context, refers to an area with varied and intricate orographical features that can significantly impact wind flow patterns. Complex terrain often includes diverse landscape elements such as hills, valleys, ridges, cliffs, boulders, trees, or human infrastructure irregularities such as buildings or wind turbines that can cause complex interactions with the incident wind ^1^. In WEA, understanding and quantifying the effects of complex terrain is crucial because these features can lead to variations in wind speed, wind direction, and turbulence, influencing the performance of wind turbines by reducing or augmenting their efficiency ^2^.

In Switzerland, the consideration of complex terrain for WEA is particularly pertinent. Policymakers have shown their intentions to phase out nuclear energy sources and transition to a fully renewable energy strategy by 2050 ^3^, which was approved by the public referendum on 21 May 2017. In a 2022 report by the Swiss Federal Office of Energy, it was concluded that nearly half of Swiss wind energy potential is located in the alpine region ^4^. This amount becomes even more impressive when the issues related to alpine wind energy are highlighted: 1) the logistics of wind turbine construction in the mountains is more challenging and costly ^5, 6^, 2) the endangerment of wildlife, and especially bird species can be unacceptable in some regions ^7^, 3) social acceptance in the form of nature and wilderness zoning, including natural horizon objections can limit wind turbine projects to several kilometres away from settlements and mountain roads or trails ^6, 8^. The recent construction of several wind parks in the complex terrain of the Alps emphasises the imperative for further detailed studies in this domain. The present study focuses only on the physical aspects of wind potential assessment, not on ecological and social acceptance issues.

## Methods

### LiDAR studies in the complex terrain

Several extensive field experiments addressing flows in the complex terrain have been conducted in the past twenty years. In 2007–2008, the Bolund experiment, ~25 km East of Copenhagen, Denmark, set the benchmark for experimental complex terrain studies using remote sensing applications such as wind-Doppler Light Detection and Ranging (LiDAR). This small 300 m wide mesa-shaped island with slopes on all directions exceeding 30° surrounded by a large body of water serves as an idealised example of showcasing the effects of terrain features on the wind ^9^. The hill was covered with 10 meteorological masts (MET-mast) ranging between 20 and 150 m in height, with a LiDAR on the far end of the peninsula and a LiDAR 300 m away on the leeward side of the hill. Based on the 60-day experimental campaign, CFD simulations were validated to understand the exact flow over the terrain feature up to 150 m above ground level (a.g.l.) ^10^. Although the Bolund experiment provides the benchmark for simulations ^11^ and data of high-resolution wind profiles ^12^ for WEA, the peninsula does not accurately represent more complex terrain such as alpine environments.

A more advanced complex terrain study in 2015–2017, specifically aimed at WEA was carried out in Perdigão, ~150 km northeast of Lisbon, Portugal ^13^. Here, two parallel hill ridge lines, separated by approx. 1500 m are covered with wind turbines. Over the three years, various measurement campaigns were held including multiple wind-Doppler LiDARs, which were used in conjunction to project three-dimensional wind velocity fields throughout the entire valley ^14, 15^. Additionally, an airborne LiDAR was used to measure wind transects along the valley ^16^. Both long-term studies of the meso-scale wind profile for WEA have been validated with LiDAR data ^17, 18^, as well as much smaller-scale turbine wake simulations ^19, 20^, proving the validity of LiDAR measurements over a wide range of spatial resolutions.

In the largest instances of complex terrain wind studies such as the Meso-scale Alpine Project (MAP) ^21^ have defined the state-of-the-art for the past 20 years. Although the study lacks specific wind energy analyses, the project serves as the basis for the validation of numerical weather prediction models in highly complex terrain ^22– 25^.

Such large-scale wind energy projects provide a wealth of detailed observational data available for high-resolution simulations of their respective study areas, however not all WEA studies merit and require such large investments. WEAs rarely operate more than one LiDAR to perform a wind energy assessment.

Single LiDAR experiments have proven to be capable of forecasting short-term wind fluctuations ^26^, as well as suitable for CFD model validation in complex terrain ^27– 29^. Kristianti *et al.* 2023 ^30^ investigated the complex alpine sites of Les Diablerets and the Lukmanier Pass, both representative of the Swiss Alps, in a comparative study to estimate the wind energy potential at these locations. Here a novel approach pioneered the use of artificial neural networks to combine weather station data with limited wind-Doppler LiDAR measurements to improve WEA. Additionally, the use of a machine learning model, called Wind-Topo ^31^, which forecasts 10 m a.g.l. wind speeds at 53 m resolution, was coupled to 100 m a.g.l. wind speeds measured by the LiDAR system using a specific transfer function. Although this transfer function could not yet be validated in a general alpine context, it proves the potential for the combination of different data sources to improve complex terrain WEA without increasing investment costs massively.

### State-of-the-art in the industry

The prevalence of terrestrial remote sensing techniques, such as LiDAR experiments, has significantly advanced the ability of WEA in scientific communities. Despite their popularity, conventional MET-masts equipped with anemometers extending up to at least two-thirds of the projected turbine hub height remain the industry standard. This preference is attributed in part to the advantage of obtaining a single-point three-dimensional wind measurement ^32^, a feature challenging for LiDAR systems, especially in complex terrains where horizontal wind continuity and uniformity cannot be guaranteed within the scanning cone ^33^. Conversely, the assumption that is made to extrapolate MET-mast measurements from two-thirds of the turbine hub height to a complete profile over the swept area by the turbine blade, usually by logarithmic wind profile theory, cannot be applied to extreme complex terrain locations ^34^.

While the concept of employing multiple LiDARs appears promising in addressing this challenge, practical issues persist in installation, maintenance, data analysis, cost and instrument availability ^35– 37^. These complexities demand an advanced understanding of the instruments and the environmental conditions, consequently contributing to elevated operational costs. Moreover, the construction expense, impracticality of installing MET-masts in mountainous regions combined with severe weather and increased risk of natural hazards further underscore the need for alternative approaches ^38^.

In response to these challenges, remote sensing methodologies employing a single wind-Doppler LiDAR have gained traction as a cost-effective and efficient alternative. In certain scenarios, sound-wave-based alternatives like Sonic Detection and Ranging (SoDAR) have been explored ^39, 40^, albeit infrequently. This is primarily due to the limitations in range and concerns about noise pollution associated with SoDARs, positioning single LiDAR WEA methodologies as the most logical step forward for prospective wind parks ^41^.

Additionally, an argument can be made for the use of LiDAR profilers instead of free-scanning LiDARs. A LiDAR profiler is designed for measuring vertical atmospheric profiles, emitting laser pulses in fixed directions, often in a conical shape, to capture wind data at a range of altitudes after which it combines the line-of-sight velocities captured in different directions to provide a volume-averaged three-dimensional wind speed of the cone swept by the laser. In contrast, a free-scanning LiDAR can point its laser beam in any direction of a hemisphere following predefined scan patterns, providing more freedom in choosing the line-of-sight wind speeds that are to be measured by the laser beam. This freedom does come at the cost that an experienced user is required to program the suitable scanning patterns and to analyse the data, particularly in the complex terrain. The cost of free-scanning LiDARs is nearly three times higher than that of a LiDAR profiler further limiting the incentive for prospective investors to choose this option. Additionally, the free-scanning LiDAR are heavier, more delicate, and have higher power demands, which limits the range of deployment in the complex terrain drastically.

### The Gotthard sequential LiDAR transect experiment

It is for these reasons that in this study, we introduce a novel approach employing a *sequential LiDAR* transect experiment utilising a single wind-Doppler LiDAR profiler. The applicability of this methodology is demonstrated in a specific case study focused on a wind farm located on the Gotthard Pass (46.55°N, 8.56°E, 2106 m a.s.l.) connecting the northern and southern alpine regions in Switzerland. Since October 2020, the upper plateau of the pass has been home to five wind turbines, forming the basis of our investigation and providing wind energy data for validation.

This work serves as a proof of concept and as a preliminary showcase of the unique data sets and analyses that are unlocked by the sequential LiDAR experimental methodology, enabling the high-resolution study of the three-dimensional wind over the entire wind park domain.

In Section 2, we provide a detailed description of the Gotthard Pass environment and the wind park site, emphasising the existing and additionally installed study-specific instrumentation used. Sections 3 and 4 outline the methodology of the sequential LiDAR transect experiment, and propose a novel method to analyse sequential LiDAR experiments. Section 5 highlights the unique wind flow over the sequential LiDAR transect including the site-specific and temporal evolution of wind profiles over the complex terrain for the Gotthard wind park, exploring the effects of the complex terrain on the airflow in the wind park and how this affects turbine performance. Sections 6 discusses and concludes on the efficacy of the sequential LiDAR method in view of logistical challenges and key findings of this experiment providing an outlook into the implications to future WEA studies.

### Field site and instrumentation

**Figure 1.**
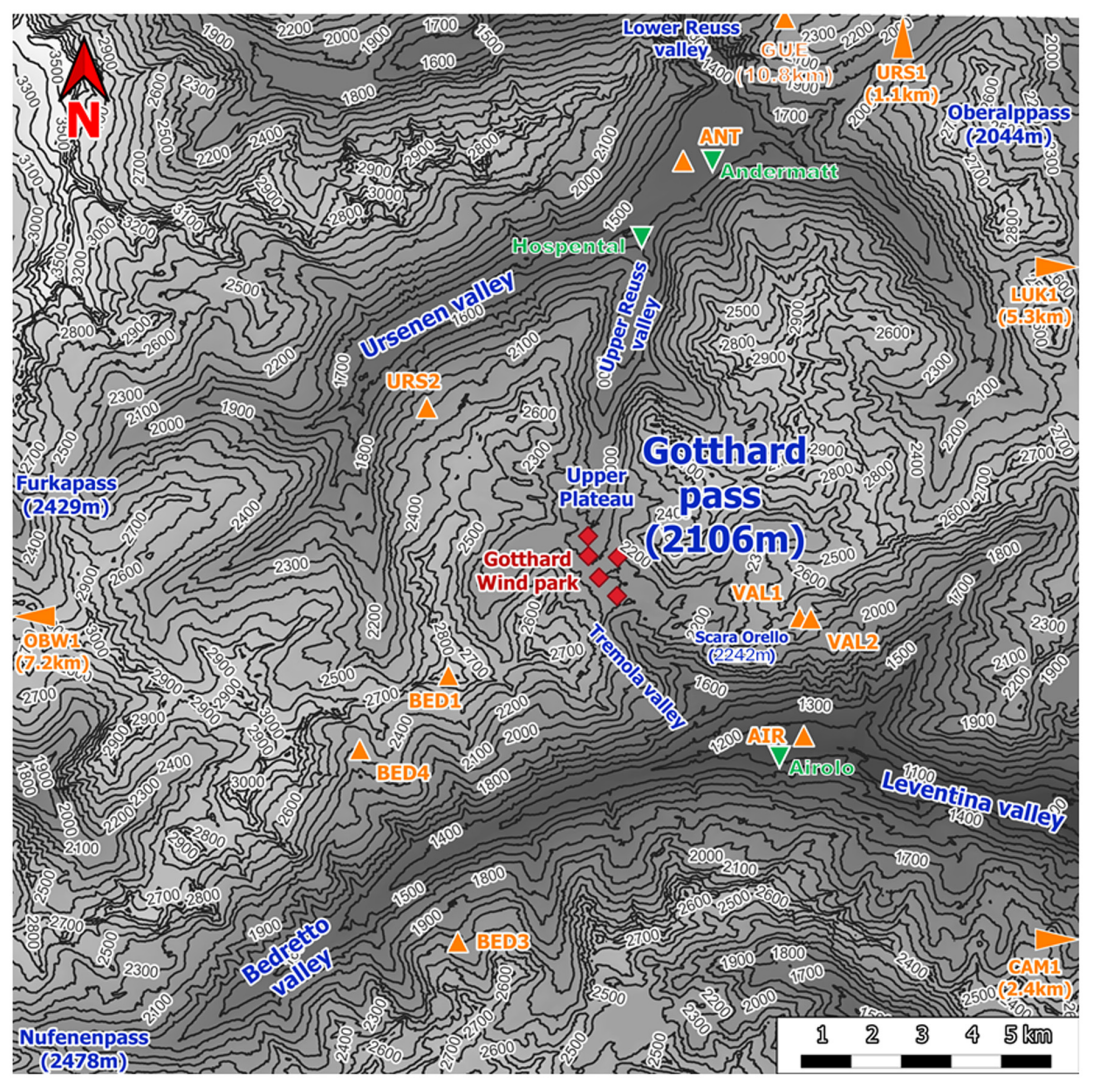
Topographical overview of the Gotthard region.

The Gotthard wind park is situated atop the Gotthard Pass at an elevation of 2106 m a.s.l. in the Swiss Alps, at approximately 46.55°N latitude and 8.56°E longitude. Figure 1 shows the Gotthard region with 100 m elevation contour lines, the Gotthard wind park turbines (red diamonds), the topographical points of interest (blue), towns (green triangles) and the surrounding weather stations (orange triangles). This pass is a saddle point between the Northern Urseren valley from the town Andermatt, canton Uri (approximately 1440 m a.s.l.) and the Southern Bedretto valley at the town of Airolo, canton Ticino (approximately 1210 m a.s.l.). From the Urseren valley, the pass ascends over a distance of 6.8 km along a near-north facing slope with an aspect of 7° up to the summit of the Gotthard Pass. This Upper Reuss valley is characterised by an average slope of 5.6° with minimal deviation and few obstacles to obstruct the wind flow. Subsequently, the pass descends over a distance of 4.3 km along a South-East facing slope with an aspect of 147° called the Tremola valley, featuring an average slope of 12.1°. From the village of Hospental to Airolo, the pass curves gradually towards the East with a radius of curvature of 7.5 km.

The central location of the Gotthard Pass is most frequently a major weather divide for the Northern and Southern Alps. Its north-south orientation provides its distinctive and characteristic feature of exhibiting both North and South Föhn winds, thus often experiencing increased wind flow on the respective lee side of the pass increasing wind energy potential ^30^.

**Figure 2.**
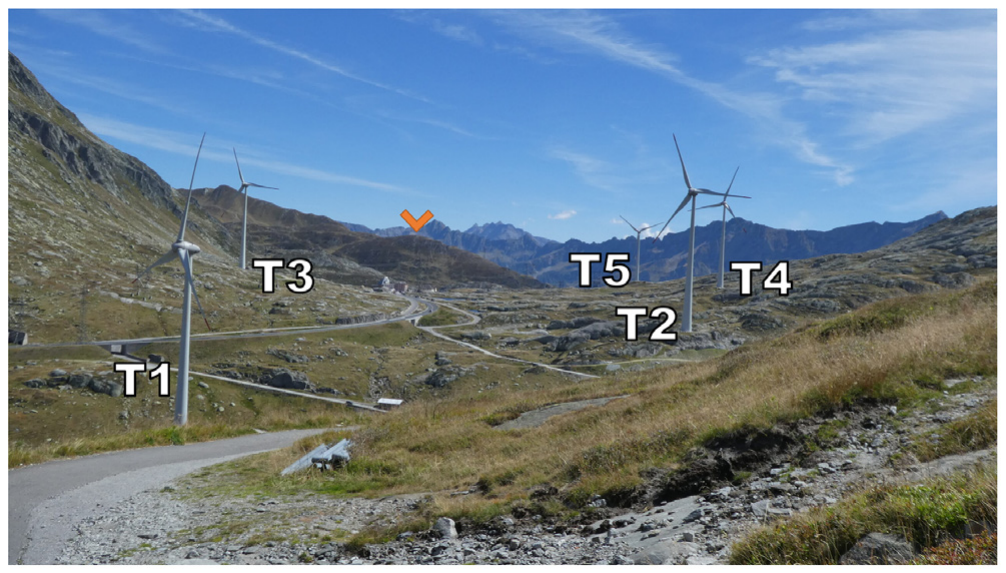
The Gotthard Pass in summer seen from the northwestern side of the Upper Plateau.

Continuing with Figure 1, the contours of the mountain pass are indicated. The southern valley, colloquially known as the Tremola valley, is notably narrower compared to its northern counterpart, with a transverse distance of approximately 300 m at an elevation of 100 m above the valley floor. In contrast, the Northern valley is wider with a valley width of 500-750 m at the 100 m above the valley floor. Moreover, the Tremola valley showcases an orographic prominence, indicated on Figure 2 (orange marker), the Scara Orello mountain (2242 m a.s.l.), located 2 km southeast of the upper plateau, blocking the free-flowing streamline of air over the eastern side of the pass in southerly winds. This situation will be discussed in more detail later. To the East and the West of the saddle point, prominent mountains reaching up several hundred meters above the upper plateau shelter the wind park from easterly and westerly winds, in fact, they are responsible for the flow channelling customarily observed in the Gotthard wind park.

**Figure 3.**
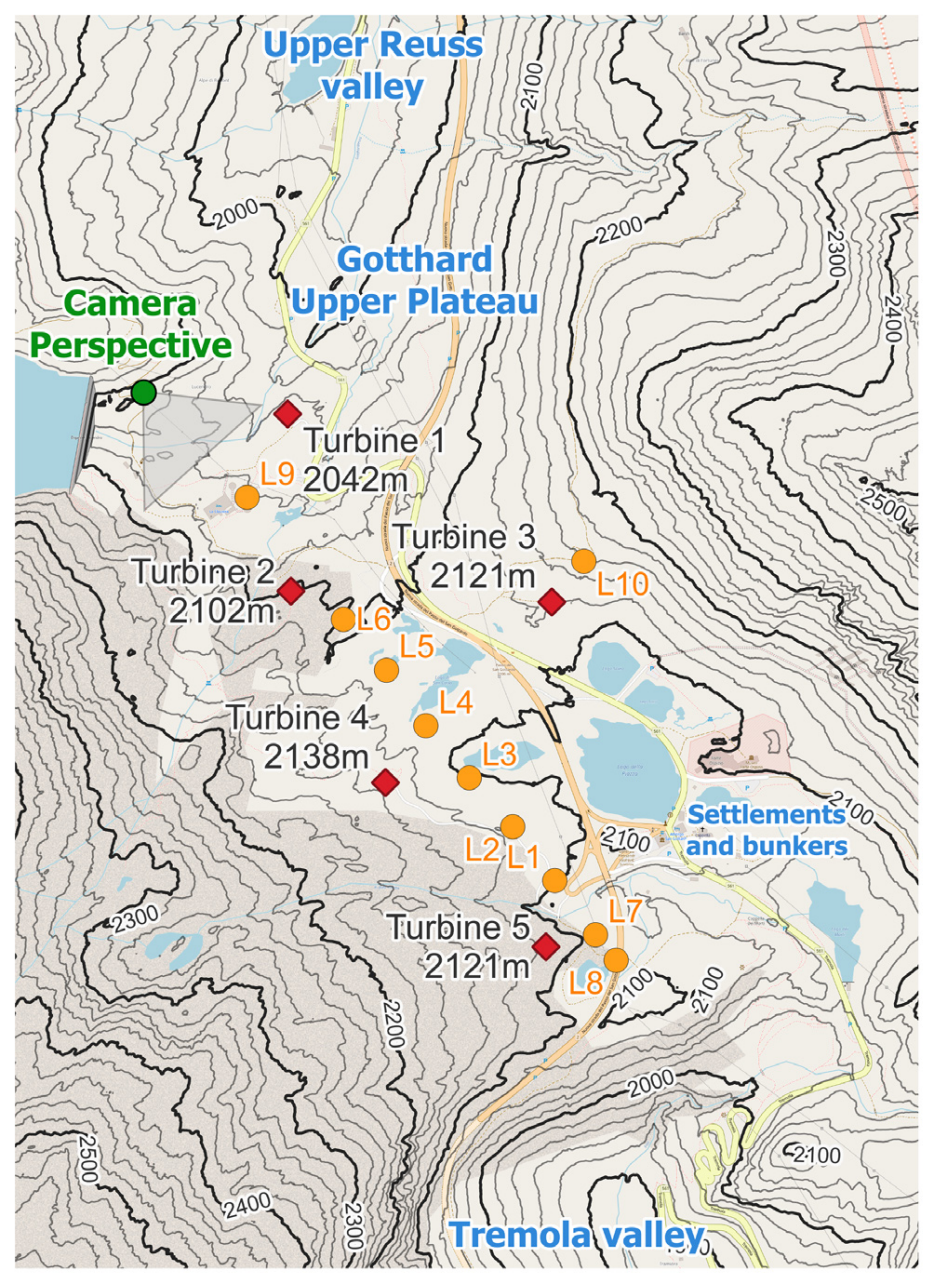
The Gotthard Upper Plateau, featuring wind turbines (red diamonds), LiDAR locations (orange dots), and the camera perspective of Figure 2.

Zooming in on the Gotthard wind park itself ( Figure 3), the Gotthard wind park comprises five turbines positioned on the upper plateau. These turbines are numbered sequentially from North to South, with Turbines 1, 2, 4, and 5 situated on the western side, while Turbine 3 occupies the eastern side. The surrounding landscape features boulders of up to 15 m tall, several small lakes, and grass patches as visible in Figure 2. Notably, the treeline is located far below the upper plateau and thus does not significantly influence the wind flow in the wind park itself. To the Southeast of Turbine 3, a small settlement consisting of low buildings and shelters is observed, exhibiting a similar surface roughness to the boulders present elsewhere on the upper plateau by estimate.

### Instrumentation overview

The five wind turbines in the Gotthard wind park are Enercon E92 turbines with a blade diameter of 92 m mounted upon a 98 m mast. They are specified for 2.35MW rated power, and a cut-in, rated, and cut-out wind speed of 2 m/s, 11 m/s, and 25 m/s, respectively. Table 1 provides details regarding the geographical coordinates, elevation, and characteristics of these turbines, as well as all instrumentation employed in this study. Firstly, the sequential LiDAR data set includes wind profiles at twenty range gates focused around the turbine height of the wind park, starting with range gates at 40 m up to 160 m increasing by steps of 10 m. After which, the range gates increase by steps of 20 m up to 300 m, to cover wind speeds well above the wind turbines to provide a comprehensive wind profile of the wind park. A range gate is a specific range interval from the LiDAR instrument within which it measures the wind speed and direction by analysing the Doppler shift of the returned signal within this interval.

**Table 1.** Instrumentation and data availability.

Dataset	Station	Data access	Easting [m]	Northing [m]	Altitude [m]	Period [ISO 8601, UTC]	Description
**LiDAR transect**	L1	Available via Zenodo DOI: 10.5281/ zenodo.14524723	2686269	1156520	2101	2023-06-19T16:20Z - 2023-06-21T22:50Z	Origin of transect
	L2		2686173	1156369	2116	2023-07-14T13:10Z - 2023-07-14T13:00Z	+150m along transect
	L3		2686074	1156747	2103	2023-07-14T13:20Z - 2023-07-21T10:50Z	+300m along transect
	L4		2685975	1156862	2110	2023-07-21T15:00Z - 2023-07-27T11:40Z	+450m along transect
	L5		2685885	1156869	2112	2023-07-27T17:50Z - 2023-08-08T13:00Z	+600m along transect
	L6		2685787	1157097	2097	2023-08-11T14:10Z - 2023-08-12T11:10Z	+750m along transect
	L7		2686362	1157400	2096	2023-08-18T16:20Z - 2023-08-29T11:10Z	-150m along transect
	L8		2686409	1156444	2089	2023-08-29T12:40Z - 2023-09-04T09:00Z	-225m along transect
	L9		2685567	1157235	2063	2023-09-04T17:50Z - 2023-09-12T08:30Z	+1100m along transect
	L10		2686324	1157325	2157	2023-09-12T13:00Z - 2023-09-20T00:00Z	Off transect near Turbine 3
**Wind Turbines (AET)**	T1	N/A	2685655	1157555	2140	2020-10-29T09:50Z - 2023-09-20T00:00Z	
	T2		2685668	1157159	2200	2020-10-08T16:00Z - 2023-09-20T00:00Z	
	T3		2686253	1157143	2219	2020-10-19T17:30Z - 2023-09-20T00:00Z	On west-facing slope
	T4		2685886	1156734	2236	2020-11-23T11:30Z - 2023-09-20T00:00Z	
	T5		2686251	1156372	2219	2020-11-16T17:40Z - 2023-09-20T00:00Z	
**Weather stations (SLF)**	BED1	Publicly available by request to WSL SLF IMIS	2682908	1154748	2963	2023-06-19T00:00Z - 2023-09-20T00:00Z	Mountain top (West)
	BED3		2683169	1149445	2101	2023-06-19T00:00Z - 2023-09-20T00:00Z	Bedretto valley (North facing)
	BED4		2681160	1153250	2305	2023-06-19T00:00Z - 2023-09-20T00:00Z	Bedretto valley (South facing)
	CAM1		2696885	1148709	2668	2023-06-19T00:00Z - 2023-09-20T00:00Z	Leventina valley (North facing)
	GOS3		2689425	1167908	2209	2023-06-19T00:00Z - 2023-09-20T00:00Z	Mountain ridge (North)
	LUK1		2703254	1163181	3040	2023-06-19T00:00Z - 2023-09-20T00:00Z	Mountain top (East, far)
	OBW1		2666706	1155765	2733	2023-06-19T00:00Z - 2023-09-20T00:00Z	Mountain top (West, far)
	URS1		2691804	1169246	2784	2023-06-19T00:00Z - 2023-09-20T00:00Z	Mountain top (North)
	URS2		2682405	1160066	2169	2023-06-19T00:00Z - 2023-09-20T00:00Z	Urseren valley (North facing)
	VAL1		2689900	1156001	2450	2023-06-19T00:00Z - 2023-09-20T00:00Z	Tremola valley (Eastern mountain top)
	VAL2		2690126	1155980	2628	2023-06-19T00:00Z - 2023-09-20T00:00Z	Tremola valley (South facing)
**Weather stations** ** (MeteoSwiss)**	ANT	Pubicly available by request to MeteoSwiss	2687445	1165044	1435	2023-06-19T00:00Z - 2023-09-20T00:00Z	North exit of Gotthard pass (Andermatt)
	AIR		2690019	1153645	1157	2023-06-19T00:00Z - 2023-09-20T00:00Z	South exit of Gotthard pass (Airolo)
	GUE		2690087	1167480	2286	2023-06-19T00:00Z - 2023-09-20T00:00Z	Mountain ridge Gütsch (North, 11km)

This large array of wind profiles is complemented by both wind speed and power metrics from individual turbines, which are made available by the turbine operators, Azienda Elettrica Ticinese, for this study to validate and compare the LiDAR profiles along the transect.

Data from eleven mountain weather stations, operated by the Swiss Institute for Snow and Avalanche Research (SLF) ^42^, and three weather stations in the vicinity of the Gotthard Pass, and on a nearby wind park at similar elevation, operated by the Swiss Federal Office for Meteorology and Climatology (MeteoSwiss) ^43^ are extracted around the Gotthard Pass to complement the meteorological data collected during this campaign.

## Sequential LiDAR methodology

To analyse the evolution of wind patterns across the complex terrain of the Gotthard wind park, a sequential LiDAR transect was executed. This transect, conducted over three months from 19 June to 20 September 2023, focuses on the upper plateau where the mountains slopes channel the prevailing wind in downwind direction. The transect includes nine distinct LiDAR locations, positioned at regular increments, plus one additional deployment near Turbine 3 on the eastern side of the plateau. This LiDAR transect aims to provide an extensive collection of wind profiles along the prevailing wind direction axis of the wind park, emphasising high-resolution, three-dimensional wind velocity fields unattainable through conventional single LiDAR deployments.

### LiDAR details

The VAISALA WindCube v2.1 WLS7-1726 profiler was the sole remote-sensing wind measurement instrument used for this campaign, sequentially situated between the five turbines in the Gotthard wind park. The LiDAR recorded wind data at twenty discrete range gates with a width of 20 m, covering elevations from 40 m to 160 m in 10 m increments. Beyond 160 m, increments increased to 20 m, reaching up to 300 m above the local ground level.

The LiDAR profiler makes use of five pulsed laser beams, which are magnetically controlled by the instrument with 0.8 s of exposure in a vertical stare position after the instrument moves the laser in 0.2 s to its next positions: North, East, South, and West with 28° angle from the vertical axis with the same exposure time. The return signal of the 1543 nm, 30 mW mean power laser is recaptured, and based on the Doppler shift of the five line-of-sight laser beams a three-dimensional wind vector is determined for each range gate in the profile.

This specific instrument logs data at two temporal resolutions: one-second, individual beam data, as well as 10-min averaged data, which can be used for both relatively high-level wind profile and shear structure analysis as well as turbulence intensity calculations for WEA purposes. Both time resolution data sets are made available via the instructions in Table 1.

### Sequential LiDAR transect

The LiDAR transect logistics involved the transport of the LiDAR by vehicle over the various wind turbine servicing roads. When the closest point to the sequential LiDAR position was reached, the LiDAR was carried up to 200 m away from infrastructure over rough terrain by two individuals. It was placed on a levelled wooden palette to minimize movement in soft soil and to protect against flooding. Power was accessed through extension cables up to 330 m long, connected to the grid via different wind turbines. The two extremes of the transect were defined by distances of -225 m and +1’100 m from the origin, with the former constrained by a highway crossing and the latter avoiding potential wake interference from Turbines 1 and 2.

A radiation-shielded combined pressure, temperature, and humidity sensor was placed near the LiDAR 1 m above the ground. This sensor provides 10-minute averaged atmospheric variables at the LiDAR site. The LiDAR wind profiles could be monitored remotely via a 4G connection using the LTE network available on the Gotthard Pass, and the data was extracted on a near-weekly basis when the LiDAR was moved to its new position.

In some cases, data gaps are observed within the LiDAR transect which occurred due to disconnection or power loss from the grid from which the LiDAR was powered. In particular, heavy thunderstorms and hail storms which are common in the Swiss high-alpine region were prone to shutting down the LiDAR measurements. Due to long access time to the study area, the LiDAR sometimes could not be serviced promptly after shutdown further extending the data gaps.

The nine sequential LiDAR locations, spaced at 150 m increments, mostly covered the western part of the wind park. The orientation of the transect was based on the prevailing wind direction extracted from the Swiss Wind Atlas providing wind rose information at 100 m above the ground level, at 100 m horizontal and 25 m vertical resolution ^44^. Additionally, a tenth LiDAR location near Turbine 3 on the eastern side aimed to capture representative wind profiles from the eastern sector.

## Sequential LiDAR inter-comparison

The primary challenge in analysing sequential LiDAR measurements lies in addressing the inherent lack of spatio-temporal continuity within the data set. As the weather is dynamic throughout the three-month measurement campaign, a method of inter-comparison of the different LiDAR deployments along the transect becomes imperative.

The method presented in this work involves the wind velocity measured on the nacelles of the five turbines situated on the upper plateau. By considering the wind vectors measured at the nacelles of each turbine, similar inflow characteristics can be deduced from which these common time frames can be compared over the entire sequential LiDAR study to show the evolution of wind profiles along the upper plateau.

These nacelle wind velocities are collected by anemometer stations installed on the nacelle, located at 98 m above ground level. These stations measure average wind speed and direction at 10-minute intervals, but do not include a measure for turbulence intensity. Within the time domain of the sequential LiDAR campaign, the horizontal wind speed components U and V are calculated for the total of 13’295 10-minute intervals covering the entire campaign period.

As shown in Figure 5, the vectorial difference in wind speed for each turbine is evaluated for each pair of 10-minute intervals (i.e., 5 ⋅ 13'295 ^2^/2 ≈ 4.4 ⋅ 10 ^8^ pairs are evaluated) in the data set ( Equation 1).


$$\;\left[\begin{array}{c} \Delta V_{ij}^{T_{1}}\\ \Delta V_{ij}^{T_{2}}\\ \Delta V_{ij}^{T_{3}}\\ \Delta V_{ij}^{T_{4}}\\ \Delta V_{ij}^{T_{5}} \end{array}\right]=\left[\begin{array}{c} \left|{\vec{v}}_{i}^{T_{1}}-{\vec{v}}_{j}^{T_{1}}\right|\\ \left|{\vec{v}}_{i}^{T_{2}}-{\vec{v}}_{j}^{T_{2}}\right|\\ \left|{\vec{v}}_{i}^{T_{3}}-{\vec{v}}_{j}^{T_{3}}\right|\\ \left|{\vec{v}}_{i}^{T_{4}}-{\vec{v}}_{j}^{T_{4}}\right|\\ \left|{\vec{v}}_{i}^{T_{5}}-{\vec{v}}_{j}^{T_{5}}\right| \end{array}\right]\;(1)$$

$$match\;({\vec{v}}_{i}^{L_{x_{1}}},{\vec{v}}_{j}^{L_{x_{2}}})=i\overset{match}{\Leftrightarrow }j=\max_{T_{x}\in \{T_{1},T_{2},T_{3},T_{4},T_{5}\}}\Delta V_{ij}^{T_{x}}\leq V_{margin}\;(2)$$

Where ${\vec{v}}_{i}^{T_{x}}$ and ${\vec{v}}_{j}^{T_{x}}$ are turbine nacelle wind velocities at time interval *i* and *j*, respectively, for turbine *T*_*x*∈{1,2,3,4,5}_ If all five turbines exhibit a wind velocity within the given error margin *V _margin_* ( Equation 2), the pair is considered to be a match. These matches are used later in the analysis to inter-compare LiDAR profiles at different time periods which were measured with sufficiently similar wind conditions.

**Figure 4.**
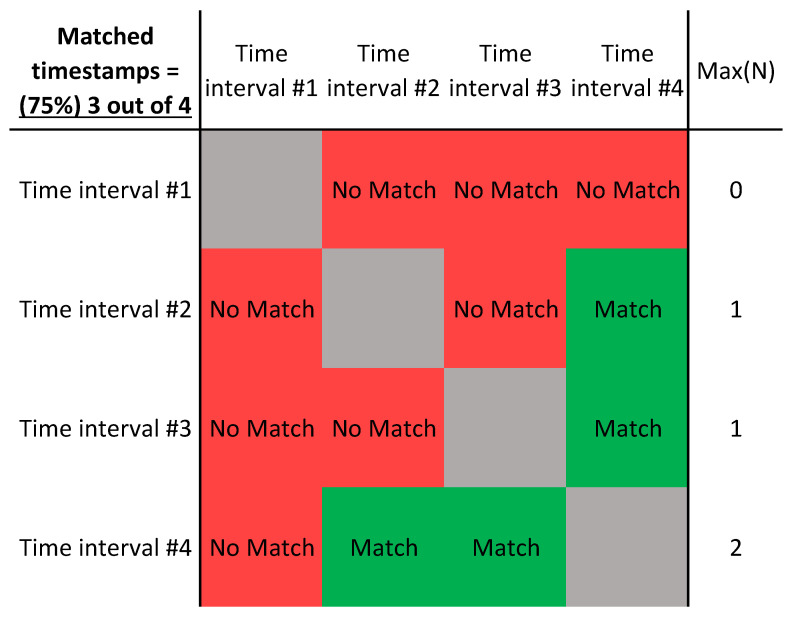
Example table of a matching algorithm where the timestamps are matched.

**Figure 5.**
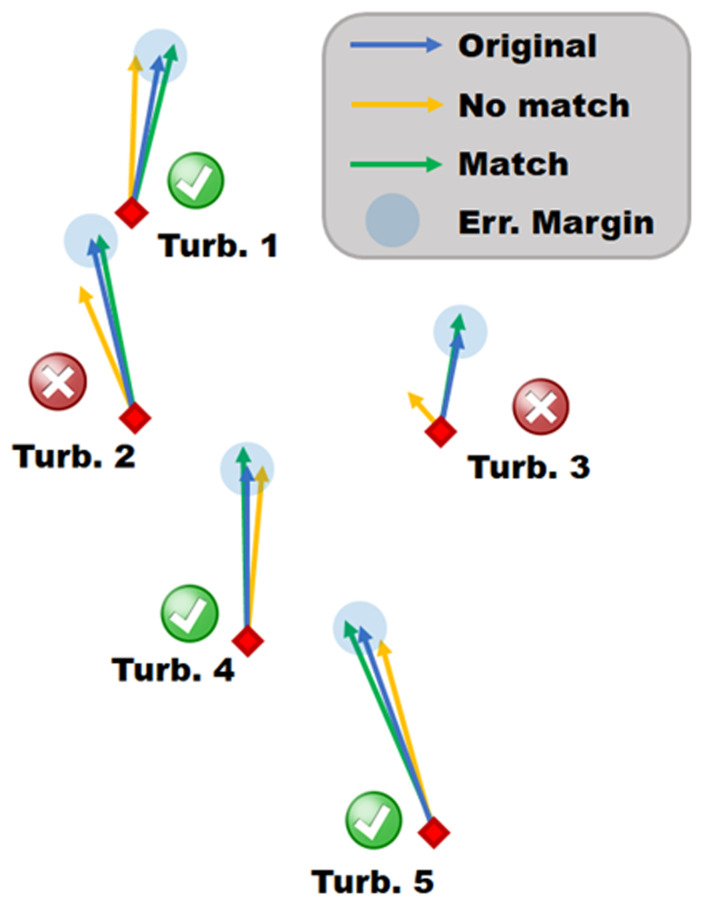
Schematic of the matching algorithm, visualize three different snapshots by coloured vectors.

As an example of the results from a matching algorithm, consider Figure 4. Here, four arbitrary 10-minute time intervals are chosen as timestamps. These timestamps are location-independent and therefore could be taken from anywhere along the sequential LiDAR transect. Note that according to the matching algorithm, any timestamp will always match with itself and is therefore disregarded (grey squares in figure). Time interval #1 does not match with any of the other time intervals, whereas time interval #4 matches with #2 and #3. Note that time intervals #2 and #3 do not necessarily have to match for #4 to have a match with both of them. This can happen if one or more wind vectors from the turbine nacelles are on the opposite sides of the velocity margin as shown in Figure 5 for Turbines 1 and 5 where the green and yellow vectors are within the blue error margin but not within the margin of each other. Finally, this table is symmetric and therefore only half of the computations must be performed to complete the algorithm.

To find the optimal value for *V _margin_*, five different error margins were tested as shown in Table 2, where the resulting matches are shown for several values for *V _margin_*. Based on the mean wind speed observed in the wind park $\bar{V}$ _windpark_ = 4.95 m/s a margin of *V _margin_* = 0.5 m/s ≈ 0.1 $\bar{V}$ *_windpark_* is chosen because of its balance between number of matches and relatively small error margin having identified 6’006 timestamps that hold at least one match. It should be noted that wind speeds ≤ 0.5 m/s are very rare, only observed in 3.6% of the data, and these low wind speed matches are disregarded due to the error margin being too large, creating inaccurate matches. The largest match includes 29 10-minute snapshots over several LiDAR locations. However, this also guarantees that some 10-minute snapshots are in the same LiDAR location, and one of these has to be chosen per LiDAR location in any particular visualisation.

**Table 2.** Comparison of *V _margin_* and the number of matches that are created.

V _margin_	Matched timestamps	max(N)
2.0 m/s	98.6% (13’112)	2’757
1.0 m/s	92.9% (12’346)	444
**0.5 m/s**	**45.2% (6’006)**	**29**
0.2 m/s	<0.01% (84)	3
0.1 m/s	<0.001% (4)	1

*Comparison of five error margins *V _margin_* with their respective number of timestamps involved in a match as well as the largest collection of timestamps in a single match for various values of *V _margin_**.

Using the velocity margin of *V _margin_* = 0.5 m/s, the measured LiDAR profiles can now be inter-compared. Since the matching algorithm described above only requires the wind turbine wind velocity data points, there are several cases where the timestamp does not have a corresponding LiDAR wind profile due to LiDAR data gaps. In fact, 4’040 of the 6’006 matches have LiDAR data available for at least two wind profiles ( ${\vec{v}}_{i}^{L_{x_{1}}}$ & ${\vec{v}}_{j}^{L_{x_{2}}}$ ) from the matched timestamps to make inter-comparison possible ( Equation 3).


$$\exists \;{\vec{v}}_{i}^{L_{x_{1}}},{\vec{v}}_{j}^{L_{x_{2}}}|L_{x_{1}},L_{x_{2}}\in \{L_{1},L_{2},\;...\;,L_{10}\},L_{x_{1}}\neq L_{x_{2}},i\overset{match}{\Leftrightarrow }j\;(3)$$

Where *L*_*x*1_ and *L*_*x*2_ indicate two unique LiDAR locations, for both of which the LiDAR profiler has measured a wind velocity at the matched time interval *i*. It should be noted that Equation 3, describes only the minimum requirement for a match to be formed, in many cases more than two different LiDAR positions are in the same match, and multiple time intervals can exist for the same LiDAR position ( Equation 4). However, such a match for *i* does not necessarily imply that *j* and *k* are also a match with each other, as their wind vectors could potentially be on the other side of the error margin (consider Turbine 1 in Figure 5).


$$match\;({\vec{v}}_{i}^{L_{x_{1}}},{\vec{v}}_{j}^{L_{x_{2}}},{\vec{v}}_{k}^{L_{x_{3}}})\to i\overset{match}{\Leftrightarrow }j,i\overset{match}{\Leftrightarrow }k\;(4)$$

**Figure 6.**
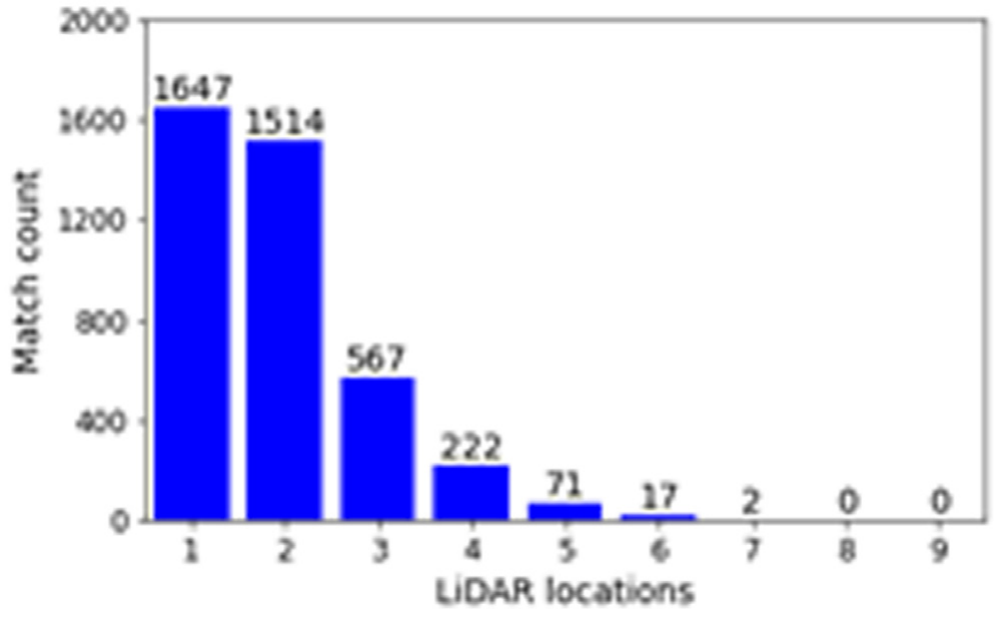
Number of matches in the dataset for *V _margin_* = 0.5 *m/s*.

The distribution of these matched profiles, which include a multitude of LiDAR locations is shown in Figure 6. A large portion of matched timestamps only have one LiDAR location. This can be explained by the fact that many matches tend to happen between two timestamps within 30 minutes of each other, as the weather system has not changed significantly yet. However, there are still many matched wind profiles that are from different locations along the sequential LiDAR transect, with even two matches, which included at least one wind profile for seven different locations along the transect.

### Variability in the LiDAR profile matching

To evaluate the robustness and variability of the proposed matching algorithm, we now consider "auto-matched" profiles, similar to Equation 4, with the only difference that the locations of *L*_*x*1_ and *L*_*x*2_ must now be equal. These profiles are constant in location, and only vary in time. Since these auto-matched profiles are matched, they are assumed to be similar in local weather conditions. As the topographical influences on the wind profile are constant for auto-matched profiles, they will become the subject of a robustness study to verify the validity of the proposed matching algorithm. In total, there are 3’778 auto-matched profiles originating from the ten LiDAR locations in the experiment. Assessing these auto-matched profiles regarding their consistency provides insight into the validity of regularly matched profiles along the transect by defining a reasonable standard deviation to the wind velocities and the turbulence kinetic energy. The matching algorithm described above collects LiDAR profiles for times when all wind turbines have similar wind flow patterns, within a 0.5 m/s error limit, and it can therefore be expected to have similar deviations in wind vectors of the auto-matched LiDAR profiles themselves. For all ten LiDAR locations, the auto-matched profiles are assessed based on their standard deviation in the three-dimensional wind vector as well as their turbulence kinetic energy (TKE). The TKE is calculated based on the LiDAR wind speed components *U*, *V* and *W*, and their respective standard deviations *σ _U_*, *σ _V_* and *σ _W_* at 10-minute intervals in Equation 5. The WindCube v2.1 used in this experiment does not distinguish between *σ _U_* and *σ _V_* however, and opts for a *σ _U _horz__* = *σ _U_* = *σ _V_* instead. Although in this study, only the 10-minute interval LiDAR data is studied, the LiDAR measures a wind profile every 5 seconds, which is later averaged to form a more robust average. With the standard deviation of the line-of-sight wind speeds, a reasonable estimate for the turbulence intensity can be made at 10 minute intervals.


$$\;k=\frac{1}{2}(\sigma_{U}^{2}+\sigma_{V}^{2}+\sigma_{W}^{2})\;(5)$$

**Figure 7.**
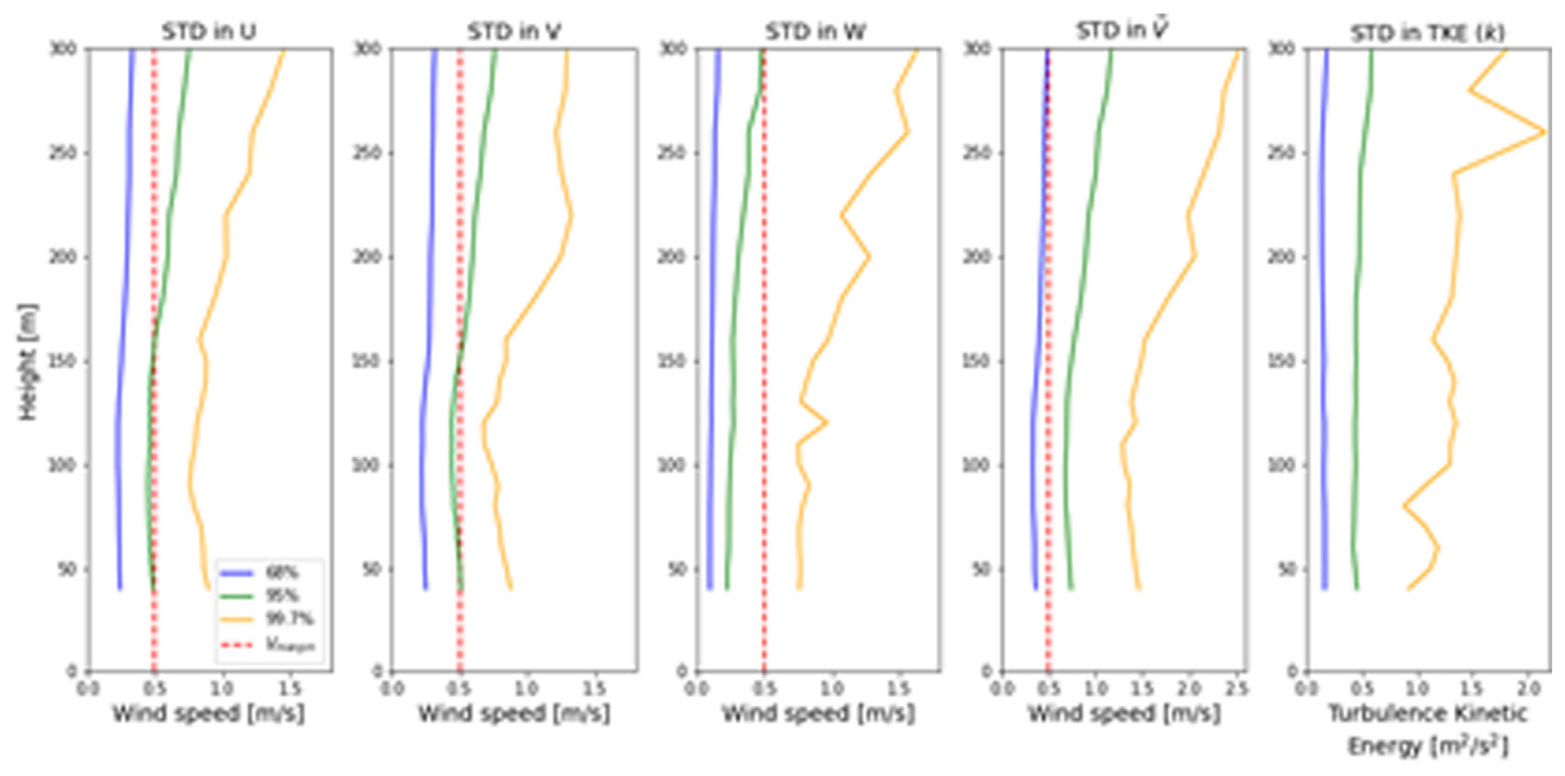
Quantiles of several wind parameters for all 3’778 auto-matched LiDAR profiles.

In Figure 7, the 68%, 95% and 99.7% quantiles of auto-matched LiDAR profiles are shown, which correspond to 1,2 and 3 standard deviations, respectively. From the standard deviation profiles shown in Figure 7, it can be observed that the majority of the auto-matched profiles are contained within the expected 0.5 m/s wind speed margin. However, in a small percentage of auto-matched profiles, the standard deviation in the wind speed does increase above *V _margin_*. This effect is stronger at the lower and upper bounds of the LiDAR profile which are furthest away from the turbine nacelles at 98 m height. This effect is not observed in the turbulence kinetic energy, which is insensitive to height. This can be explained by the gradual reduction in turbulence as we move up in the atmospheric boundary layer, which balances out the continuous increase of turbulence kinetic energy in the range of the LiDAR profiles.

In addition to this, the introduction of vertical wind speed *W* to the wind vector $\bar{V}$ increases the bias towards larger standard deviations in the wind speed; this is particularly visible for the 99.7% quantile. This can be explained by the fact that the nacelle wind sensor only measures horizontal wind speed, and therefore the matching algorithm can not use the vertical wind component to assess whether two time intervals have similar vertical winds. Since this only becomes problematic for less than 1% of the matched profiles, a simple visual inspection of the three-dimensional wind field is sufficient to identify faulty vertical wind speed matches.

On the other hand, the atmospheric variables such as barometric pressure, air temperature and absolute humidity do not correlate well with the auto-matching algorithm and large variations can be observed, which indicates that the proposed matching algorithm is coherent in selecting similar wind flow patterns, but not in selecting similar weather conditions. This directly implies that atmospheric stability is also not necessarily constant throughout these matches. The lack of correlations in these atmospheric parameters can also be observed when using the atmospheric variables measured by the thirteen weather stations around the pass.

Thus, to analyse typical alpine wind events such as Föhn winds, which depend on humidity, air temperature, and pressure differences at both sides of a mountain, a deeper investigation into the atmospheric parameters for all timestamps involved in a match must be performed before such an event can be studied.

## Results

Having established a robust matching algorithm for the sequential LiDAR measurements along the transect in the Gotthard wind park, the matched wind profiles can now be visualised along the transect, with all three wind components along the pass at any height of the measurement range. Previously, such visualisations were only possible using a triple wind-Doppler LiDAR setup with synchronised measurements.

### Gotthard Pass wind characteristics

**Figure 8.**
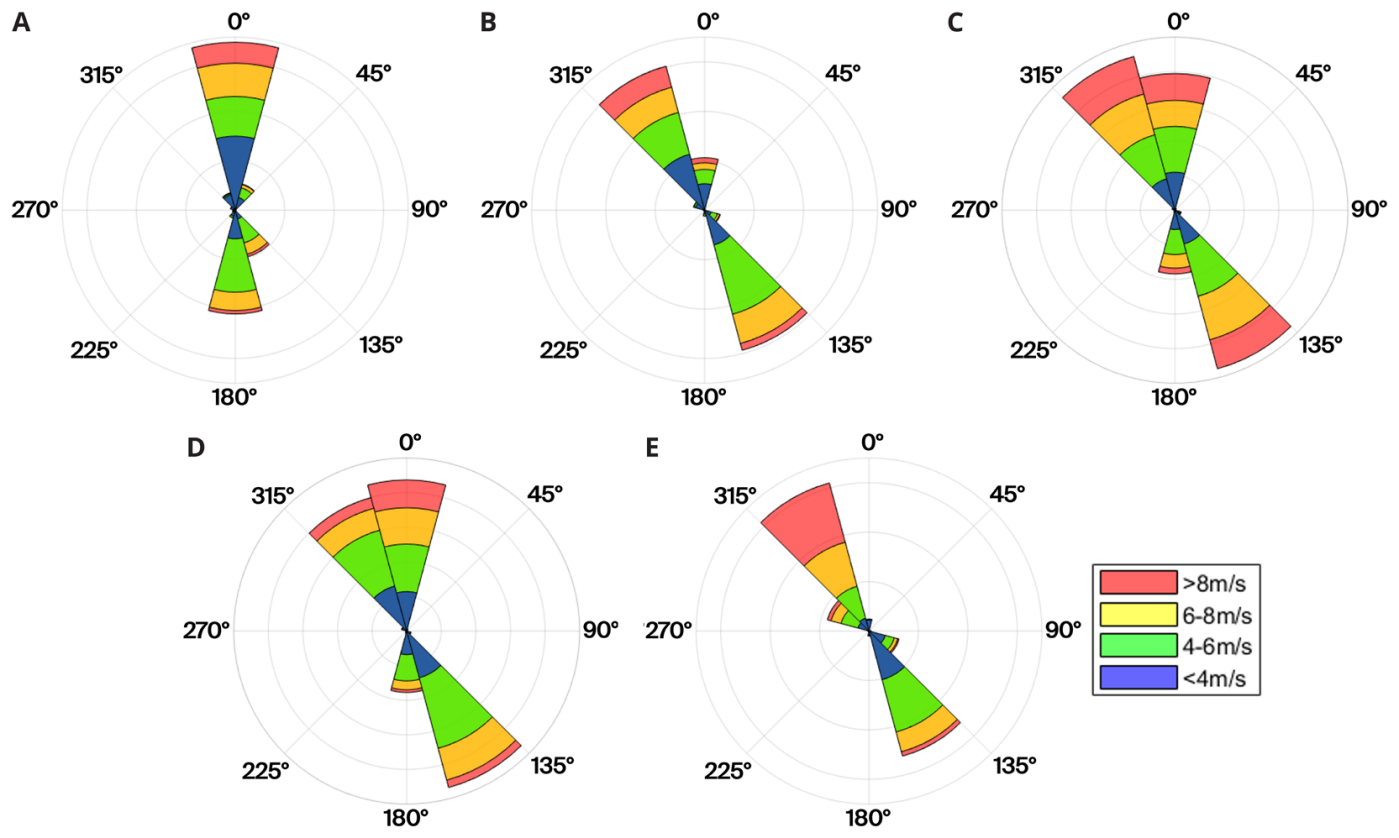
Wind roses of the five turbines in the Gotthard Wind Park.

Firstly, the wind patterns observed on the pass at the height of the turbine nacelles are illustrated by wind roses in Figure 8. Here, northerly winds can be observed to be generally stronger than southerly winds. Particularly, wind speeds above the rated wind speed of the E92 turbine are much more prevalent. We define southerly winds as originating between the azimuths 75° and 255° and northerly wind from the remaining 180° sector. These distinct differences can be recognised in the Weibull distributions ( Equation 6), which are split based on the two prevailing wind directions in Table 3.

**Table 3.** Weibull fit parameters of the Gotthard Wind Park for Northerly and Southerly winds.

Turbine	Northerly wind			Southerly wind		
	*k*	*A*	$\left|\vec{\text{v}}\right|$	*k*	*A*	$\left|\vec{\text{v}}\right|$
**1**	1.31	5.14	4.16	3.53	5.27	4.76
**2**	1.93	5.31	4.67	3.38	5.38	4.84
**3**	2.33	6.65	5.75	2.60	6.29	5.52
**4**	2.54	5.89	5.11	3.03	5.18	4.67
**5**	2.64	7.66	6.59	2.62	4.80	4.31


$$\;f(v)=\frac{k}{A}{\left(\frac{x}{A}\right)}^{k-1}\exp-{\left(x/A\right)}^{k}\;(6)$$

*Wind roses at the nacelle height of each turbine based on 3 years of wind direction data*.

*Weibull parameters scale shape k [-] and A [m/s] parameters, and the measured mean wind speed $\left|\vec{v}\right|$ [m/s], split for northerly and southerly winds (75° < θ ≤ 255°) for the Gotthard wind turbines*.

**Figure 9.**
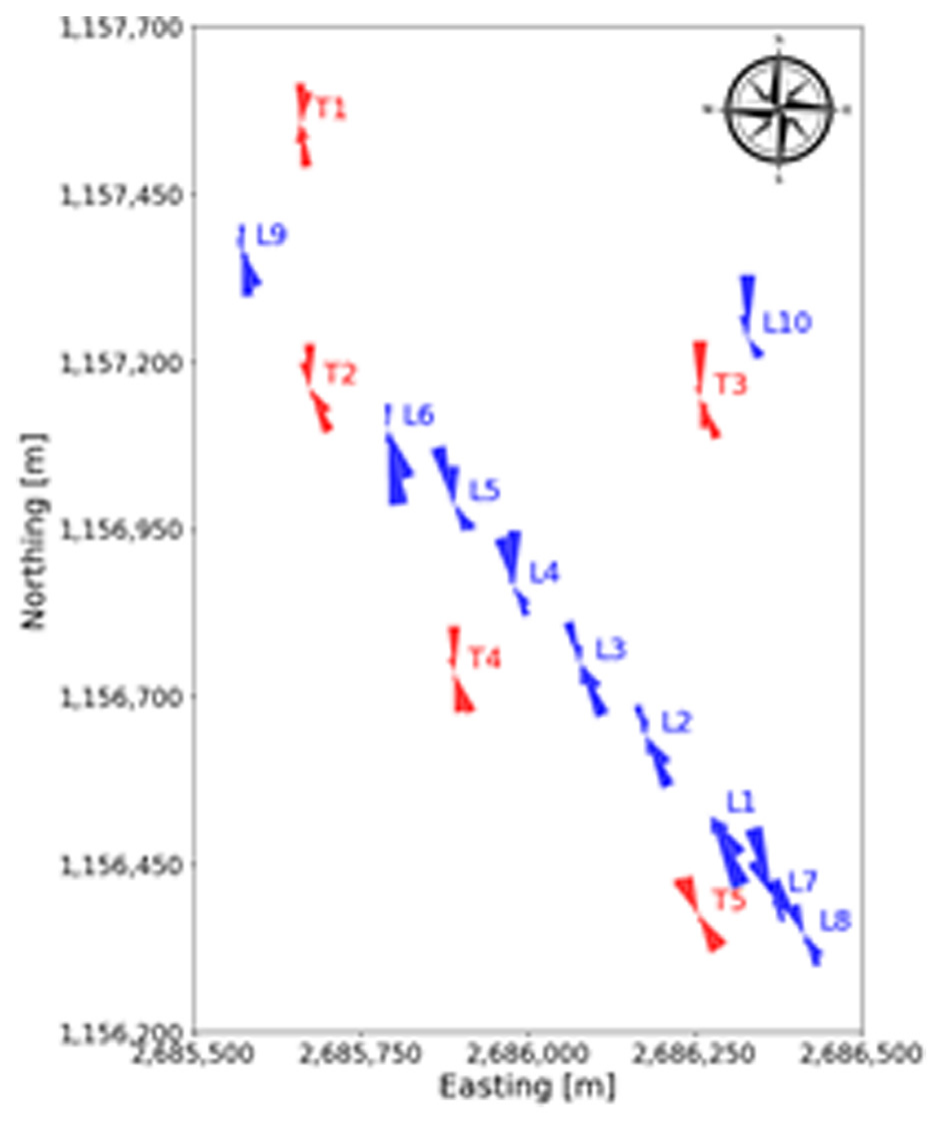
Spatial progression of wind direction over the Gotthard wind park, illustrated by wind roses at around 100 m above ground level.

In Figure 9, the wind roses at approximately 100 m above the ground of all the turbine and LiDAR locations are shown. The blue wind roses that are generated from LiDAR data do not exhibit the correct proportions in the frequency of the wind direction, as often only one week of data is available at a given location. However, the wind roses illustrate the spatial progression of the general wind direction along the Gotthard wind park well. Particularly, the differences for northerly and southerly winds at any given location on the pass can be observed.

### Transect wind profile evolution

**Figure 10.**
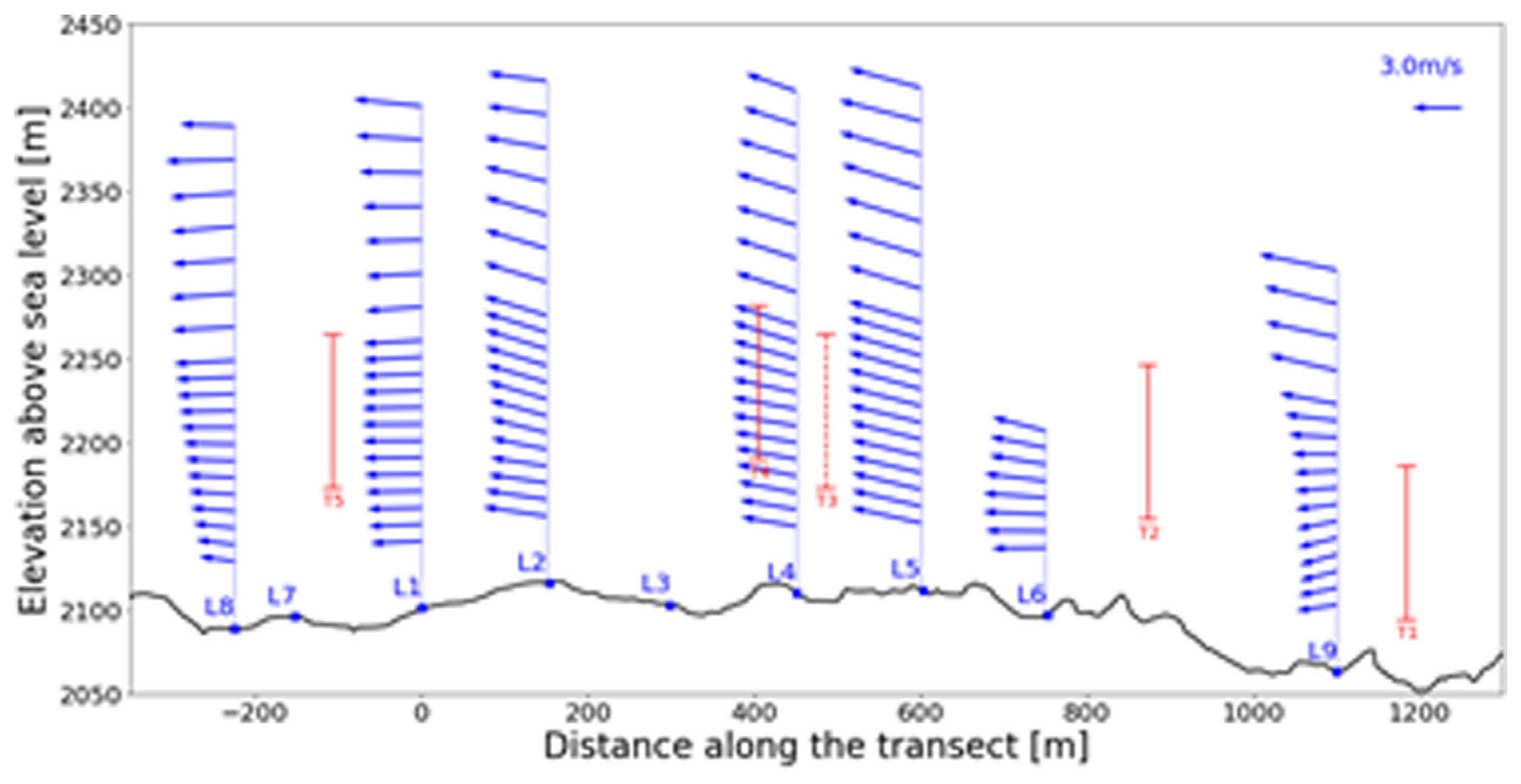
Side view of a Northerly wind on the Gotthard Pass. Swept areas of turbine rotors are shown in red.

**Figure 11.**
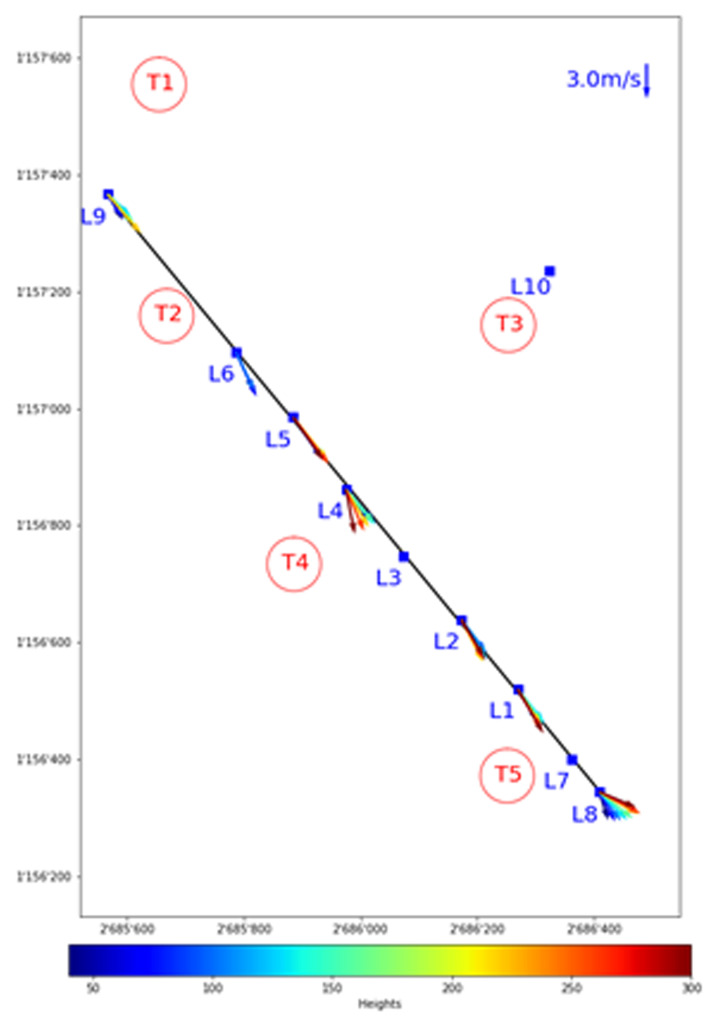
Top view of a Northerly wind on the Gotthard Pass. Height in meters is indicated by colour.

Consider the northerly wind visualisation in Figure 10 and Figure 11, where one of the largest matches is visualised in side and top view, respectively. The seven LiDAR profiles were measured at different dates as well as time of day as indicated in Table 4. In the side view shown in Figure 10, the span of the wind turbine blades is indicated as red line. Since the transect runs through the western side of the Gotthard wind park, Turbines 1, 2, 4, and 5 are most relevant, whereas Turbine 3 is indicated with a dotted line as it is much further away from the transect and to the side. For the top view shown in Figure 11, the span of the turbines is indicated by a red circle with radius 46 m. Additionally, the tenth LiDAR profile is excluded from this side view of the transect as it is not located on the transect itself. For this wind profile, the top view of the Gotthard wind park in Figure 11 can be considered.

**Table 4.** LiDAR transect metadata of the match displayed in Figure 10 and Figure 11.

LiDAR	X_transect [m]	Date	Time [UTC]	Height range [m]
**L8**	-225	02.09.2023	17:50	40–300
**L7**	-150	N/A	N/A	N/A
**L1**	0	19.06.2023	18:30	40–300
**L2**	150	07.07.2023	08:40	40–300
**L3**	300	N/A	N/A	N/A
**L4**	450	23.07.2023	06:00	40–300
**L5**	600	28.07.2023	13:50	40–300
**L6**	750	12.08.2023	06:40	40–110
**L9**	1100	10.09.2023	13:40	40–240
**L10**	Off transect	N/A	N/A	N/A

In Figure 10, the wind can be observed coming from the North with an upward wind component aloft, following the topography of the Reuss valley. As the wind moves over the wind park plateau, the wind closer to the surface and around the turbine height becomes mainly horizontal and accelerates with L9 and L1 experiencing an average wind speed of 2.63 m/s and 3.59 m/s in the turbine blade swept area (approx. 40–160m a.s.l.), respectively. This acceleration of the wind over the pass is generally observed for northerly winds. In L8, we observe a strong shear, and a decrease in average wind speed between 40–160m a.s.l. at 3.09 m/s, but the wind speed at the upper end is 44% higher than at the lower end of the turbine swept area. Furthermore, the wind vectors start curving downward as the flow approaches the Tremola valley, again following the terrain. This acceleration with northerly winds along the wind park can also be observed in the turbine energy output data: the southern Turbines 3 and 5 have a distinct increase in energy production for any medium to high northerly wind speed.

To explore other complex terrain wind phenomena that may impact turbine efficiency, not only the wind acceleration and vertical wind speed components can be studied. Shear and veer evolution along the pass can often be observed, as well as three-dimensional turbulence intensities evolving over the wind park can be identified and visualised with the sequential LiDAR data set, as discussed in the following sections.

### Wind shear and veer

For northerly wind, at the southern end of the transect, close to LiDAR location L8 ( Figure 10), a clear shearing situation is visible. This shear is observed for any northerly wind over the Gotthard Pass, and is attributed to the small hill on which Turbine 5 is located. Similar shear patterns are observed around Turbine 5 for southerly winds, but they are less pronounced due to the sharp escarpment facing North, while it has a smoother South facing slope.

**Figure 12.**
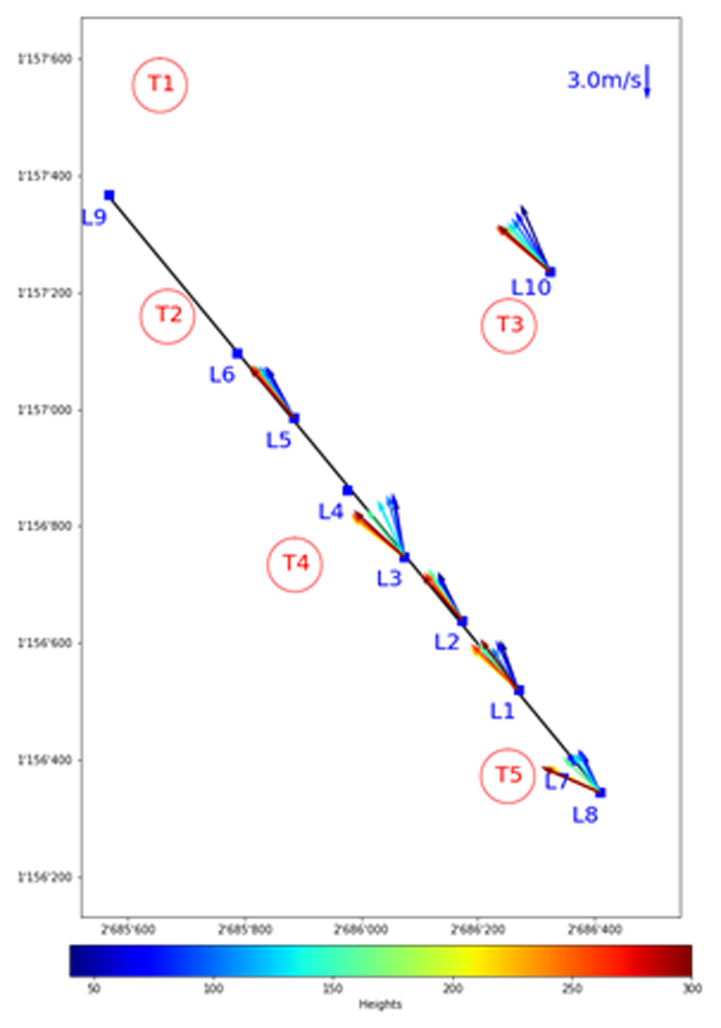
Top view of a southerly wind shear, most likely caused by the Scara Orello mountain.

In fact, for southerly winds a different orographic prominence, the Scara Orello mountain (2242 m) in the Tremola Valley (see Figure 2 and Figure 3), causes a much larger disturbance to the airflow over the Gotthard wind park. Consider Figure 12, where the top view of a southerly wind profile is visualised over the Gotthard Pass. The red vectors, between 250 and 300 m above ground are pointing in a north-westerly direction, whereas the vectors closer to the ground are pointing primarily north. This horizontal veering is attributed to the influence of the Scara Orello prominence, which stands at 140 m above the Gotthard wind park. The winds at higher altitudes, which can pass over the prominence are not affected much, and can therefore follow a straight streamline pointing northwest, as the lower Tremola valley has originally channelled the wind in this direction. The lower airflow is forced around the Scara Orello such that it then is redirected directly North over the pass.

**Figure 13.**
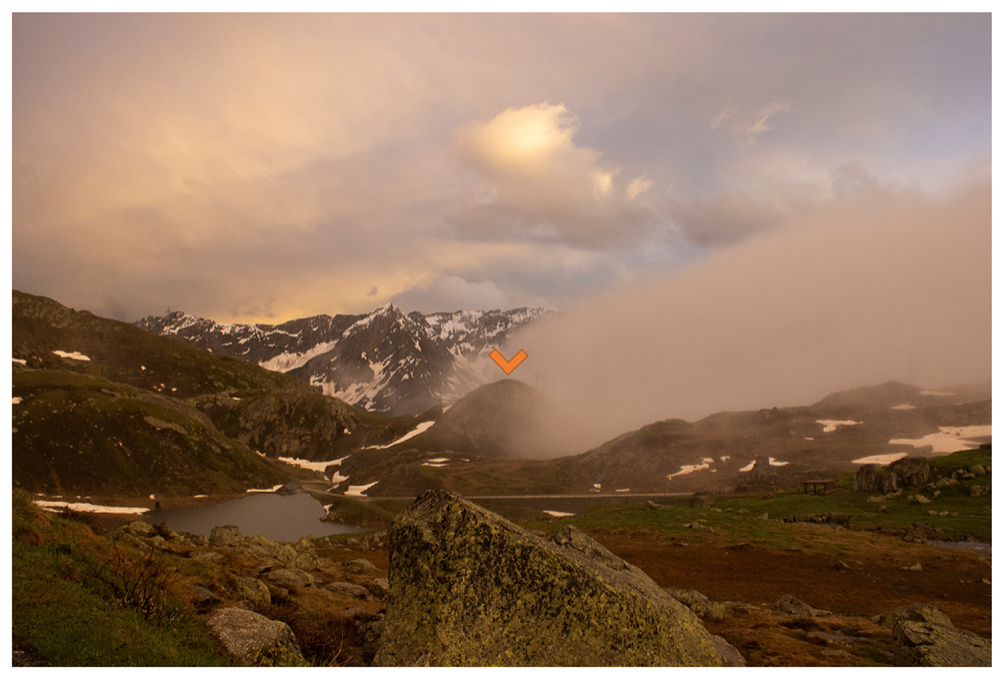
Indication by the cloud layer of the horizontal stratification for southerly winds due to the Scara Orello mountain (orange).

This separation in the air layers around the turbine height at the Gotthard wind park is likely affecting the wind turbine performance of Turbines 5 and 4. Turbine 3 on the other hand, seems to be less affected by this wind veer as this turbine is located higher than the western turbines and reaches the upper air layer primarily. This horizontal stratification of the air can occasionally be observed by the eye such as in Figure 13, where low hanging clouds move around the Scara Orello and flow exclusively on the western side of the upper plateau, whereas the eastern part, where Turbine 3 is located, remains cloud-free.

### Turbulence kinetic energy

**Figure 14.**
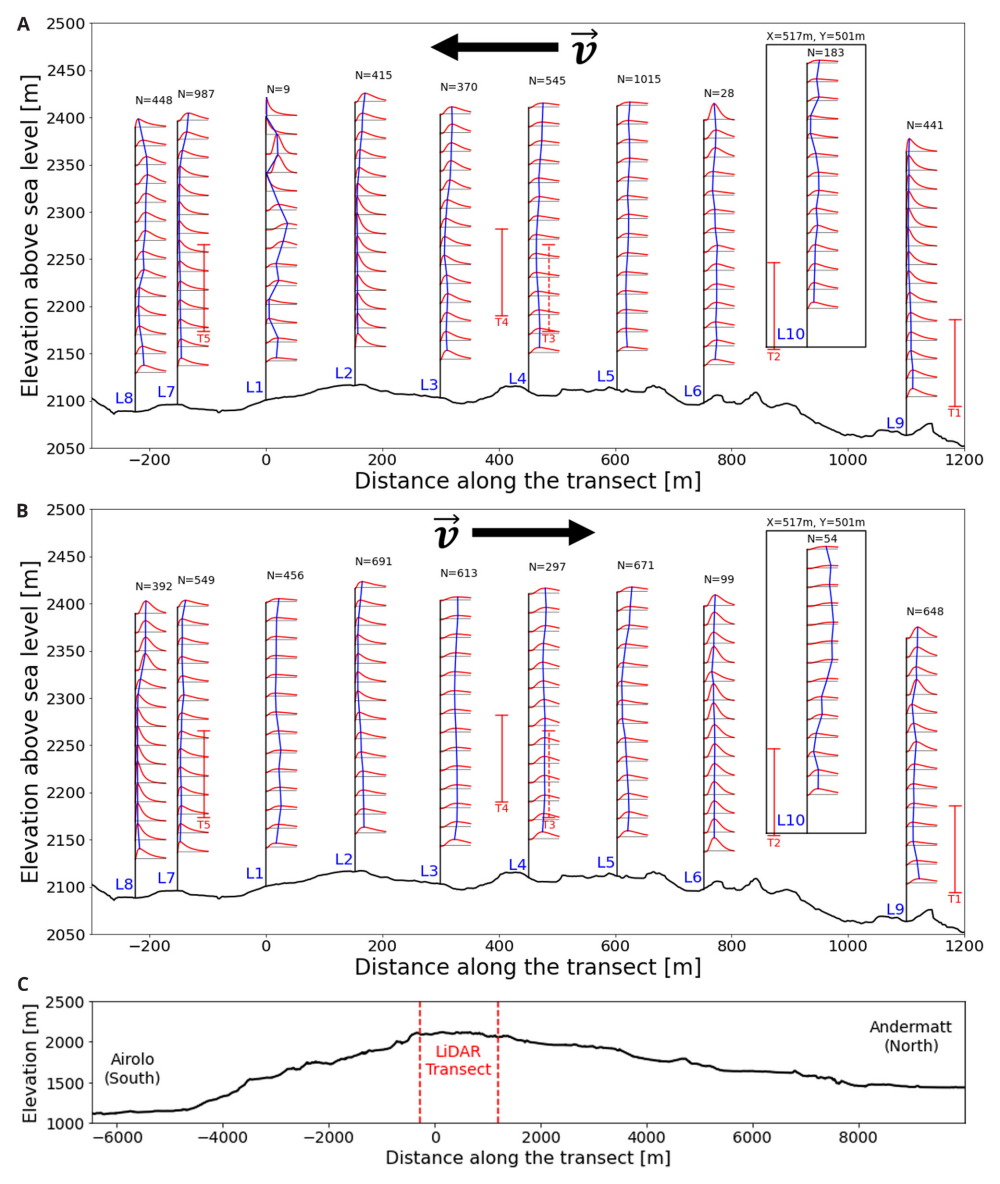
Log-normal fitted TKE distributions along the Gotthard pass. **a**) Northerly winds, **b**) Southerly winds, **c**) extended elevation profile.

Turbulence kinetic energy (TKE) *k*, Equation 5) exhibits bi-modal distributions, similar to the wind speed, along the LiDAR transect as shown in Figure 14.

For both northerly and southerly winds, the air enters the wind park at low turbulence levels. As the air moves further over the pass, the turbulence kinetic energy gradually increases. This is to be expected as the wind tends to accelerate on the upper plateau due to the increased density of streamlines as the air is pushed over the highest point of the pass and the overall kinetic energy of the wind increases, this will also contribute partly to the increased turbulence kinetic energy. Additionally, the surface obstacles, such as boulders, buildings and wind turbines will partly contribute to the increase of turbulence in the flow. For southerly winds in Figure 14(b), the increased turbulence in the lee of Turbine 5 can be observed in L1, L2 and L3 around the swept area of the turbine. This effect is not observed for northerly winds, which can be explained by the fact that these LiDAR locations are not directly in the lee of T2 and T4.

Near Turbine 3 on the eastern side of the pass measured by L10, the greatest turbulence kinetic energy can be observed for southerly winds. This correlates with the increased southerly wind speeds which were observed during the measurement period at this site. Additionally, the upwind terrain on this side of the pass is notably more complex, with the aforementioned Scara Orello mountain (2242m) obstructing the wind for the western side of the pass. This will likely affect the flow on the eastern pass in a similar way.

We illustrate for the case of a symmetric deviation from a mean wind speed with deviation magnitude sigma, which is the average magnitude of deviations by definition, how the effect of non-linearity results in additional power that can be harvested. If we further assume that such a deviation gives the approximate magnitude of the power gain by turbulence, we can estimate the effective energy the turbulence holds for the type E-92 turbine on the Gotthard plateau. Consider the wind turbine power Equation 7:


$$\;P(v)=\frac{1}{2}\rho Av^{3}\;(7)$$

*ρ* = 0.996 ± 0.021[ *kg/m* ^3^] is the air density on the Gotthardpass, *A* = *πr* ^2^ = 6650[ *m* ^2^] the swept area of a wind turbine, *v*[ *m*/ *s*] the incident wind speed. Since the horizontal standard deviation of the wind for the WindCube is defined by *σ _u_* = *σ _v_* = *σ _horz_*, we can consider a deviation *σ _horz_* above and below the regular wind speed *ν*. As the power scales with *ν* ^3^, the positive deviation increases the turbine power more than the negative deviation diminishes it. The difference in power due to this non-linearity is given in 8:


$$P_{turbulence}=\frac{P(v+\sigma_{horz})+P(v-\sigma_{horz})}{2}-P(v)=\frac{3}{2}\rho \;A\;v\;\sigma_{horz}^{2}\;(8)$$

It should be noted that only a part of this turbulence kinetic energy can be transferred to the blades and therefore this value can only be used as an upper-bound. Additionally, this equation assumes that the wind speed deviations to the mean wind speed are symmetrical until the second order (e.g. normal distribution), the turbulence power can be considered only as a rough upper-bound to the influence of turbulence on the wind turbine performance. In the region between the cut-in wind speed and the rated wind speed, these turbulence contributions increase turbine power output. At lower wind speeds the turbine will not generate energy from this turbulence, and at higher wind speeds the positive effect *P*( *v* + *σ _horz_*) → 0, as the turbine does not generate additional energy as the power curve flattens. Finally, this equation assumes that the power is propotionally to the velocity cubed in the entire domain, which holds only between the cut-in and rated wind speeds of the turbine. Outside this domain, deviations will reduce as the power becomes constant with velocity.

Averaging over all locations and heights measured during the LiDAR campaign, combined with the all-time average wind speed in the Gotthard wind park of $\bar{v}$ = 4.95 *m/s*, we find that the northerly and southerly turbulence power upper-bound is 9.9kW and 16.2kW, respectively. Compared to the all-time average wind turbine power of 370.9kW, their respective positive contributions to power production can be estimated to be lower than 2.4% and 4.4%, respectively but it can increase to by multiple factors in exceptionally windy conditions and highly turbulent locations such as Turbine 3. However, it would be incorrect not to discuss that the existence of turbulence, in fact reduces the overall kinetic energy of the wind, thus a non-turbulent airflow would generate more wind energy. This perceived increase in power is only present because the nacelle-mounted anemometer can only sense the average wind speed at 10-min resolution, disregarding the turbulence variations in totality.

### Regional weather correlations

Subsequently, based on the matching timestamps, the IMIS and SLF stations were assessed in an attempt to find correlations and patterns between the wind on the Gotthard Pass and other atmospheric variables in the vicinity of the pass. These correlations can be of interest in generalising the local high-resolution measurements to nearby locations, enabling an extended domain of the wind energy assessment. Additionally, potential correlations can help in future case studies to characterise specific weather systems from weather models to predict wind profiles around the study area itself.

Regarding the mountainside- and mountaintop-located IMIS stations, which measure at 1 hour resolution, no strong correlations (| *r*| < 0.4) can be found in any of the atmospheric variables and wind speed or direction compared to any data collected during the Gotthard 2023 campaign. The mountaintop stations provide moderately better correlations as they better represent the synoptic weather conditions and are less prone to small-scale local topographical effects. A simple model to predict northerly or southerly winds in the Gotthard wind park remains too challenging for simple linear correlation models, however. The mountainside stations generally had worse correlations (| *r*| < 0.3). This is attributed largely to the different slope aspects of the sites where the stations are located, far away from the Gotthard wind park itself.

The MeteoSwiss stations in the towns of Andermatt and Airolo provide a stronger correlation in form of temperature difference between the termini of the Gotthard Pass. This can be interpreted analog to the Föhn index on the northern and southern end of the pass, which is pre-calculated for a small amount of MeteoSwiss stations ^45^. During Föhn events, the temperature in the downwind valley is always observed to be higher than on the windward side at the same elevation, particularly during occurrences of stronger winds in the wind park. We chose to use the temperature difference between Airolo and Andermatt over the Föhn index in an attempt to study Föhn events, as it provides a continuously correlating variable to explain discrepancies in wind turbine efficiency, but also still encompasses northerly and southerly Föhn events. Additionally, since the matching algorithm does not necessarily perform well in combining similar regional weather conditions, the temperature difference remains as the most suitable choice.

Finally, the MeteoSwiss weather station Gütsch installed 11 km NNE of the Gotthard wind park, with an elevation of 180 m above the Gotthard wind park on an east-west ridgeline provides the best data for this analysis. Although the wind components measured at Gütsch still lack correlation with those in the Gotthard wind park, barometric pressure, air temperature and humidity strongly correlate with the variables that were measured during at Gotthard Pass during the 2023 campaign. Particularly, temperature and pressure can be used to approximate their equivalence on Gotthard when our instruments are not present or had data gaps by using a lapse rate of 6.5°C per km (+1.7°C on Gotthard) and the barometric equation (+17.42 ± 0.36 *hPa* on Gotthard), respectively. The relative humidity is less correlating to that measured on Gotthard, however, since it is only used for estimating air density on the Gotthard Pass, for which it has a low sensitivity to errors, we assume the same humidity for both locations. This estimated air density is used to estimate the general turbine efficiency decrease, which can not be attributed to complex terrain interactions in the following section.

### Wind turbine efficiency

**Figure 15.**
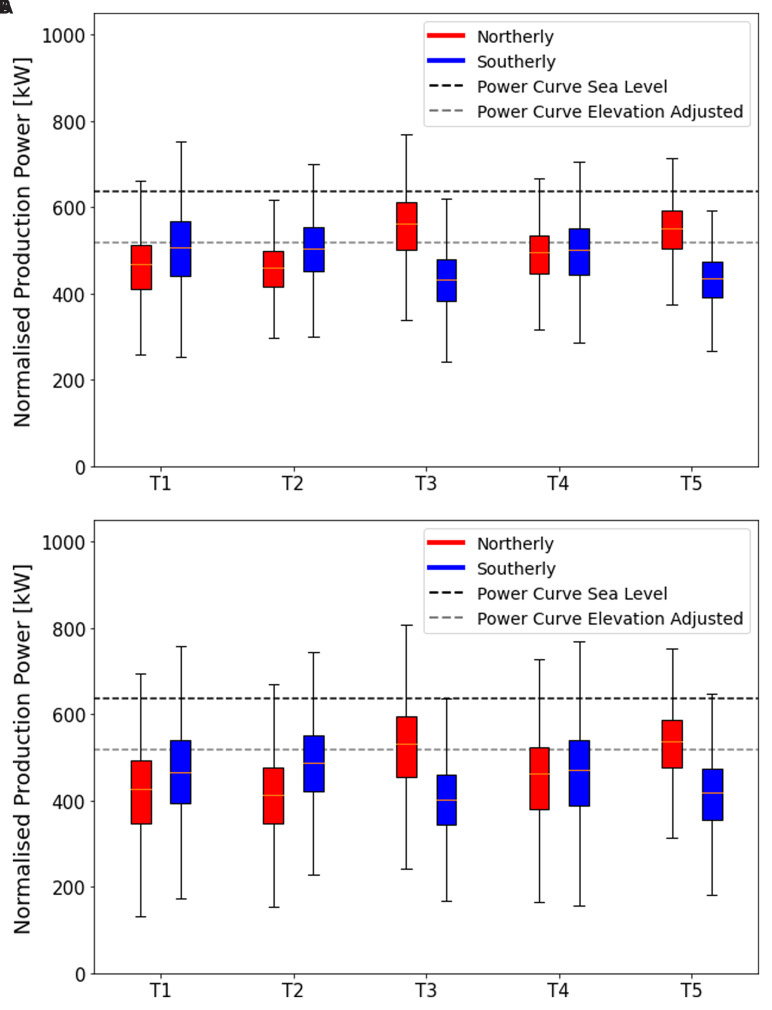
Power generated by turbine and wind directions. ( **a**) summer campaign period (19 June 2023 - 20 September 2023), ( **b**) the entire dataset (October 2020 - September 2023). In Figure **15a** where only the power data during the measurement campaign is shown, consider the northern Turbines 1 and 2. The southerly winds are more efficient at transferring energy to the wind turbine than their northerly counterparts. This discrepancy can be explained partly by the increased turbulence as the wind moves over the upper plateau before it reaches these turbines. For the northerly winds there is still a significant vertical wind speed component incident on the turbines increasing blade stresses and, making the programmed angle of attack of the turbine blades less efficient.

The bi-directional wind system across the Gotthard is prevalent for the wind shear, wind veer and turbulence on the pass. The compound effects of these combine, and occasionally cancel, each other when the air parcels transfer their energy to the wind turbine blades. To study the impact of terrain and flow effects on the efficiency of the five wind turbines, the production power distributions for northerly and southerly winds are shown in Figure 15. Wind speeds are selected between the cut-in speed and the rated wind speed, where the relation *P* ∝ *v* ^3^ holds. In the range of 4 m/s ≤ *v* ≤ 10 m/s, small disturbances and perturbations of the flow have the largest impact on power production. The power production is normalised to 7 m/s by the cubic relation between power and wind speed in this range.

Remaining in Figure 15a, the central Turbine 4, generally experiences the benefits of the northerly and southerly winds equally with very low turbulence for both directions as well as moderate wind shear and veer only slightly reducing the turbine efficiency. Vertical wind components play almost no role for this turbine.

The most interesting sites are Turbines 5 and 3 during the campaign period, shown in Figure 15a. These turbines not only benefit from flow acceleration for northerly winds, which generally bring higher wind speeds to this part of the pass. Remarkably, they can also transfer this energy more efficiently than expected when referring to the manufacturer’s power curve when corrected for the reduced air density on the Gotthard Pass as a result of the elevation. This apparent out performance can be explained by the increased turbulence which reduces the average wind speeds to lower and unrealistic values due to the limitations of the nacelle-mounted anemometer, whilst the kinetic energy of the wind is higher than indicated. These effects are exaggerated most for the strong northerly winds which accelerate to much greater wind speeds before reaching these turbines due to the linear scaling with the average wind speed *v*, as shown by Equation 8. The wind shear and wind veer for these northerly winds are already reduced to a minimum as observed for southerly winds for Turbines 1 and 2, and thus do not decrease the performance, whilst the strong shear and veer for southerly winds, as observed in Figure 12, has a detrimental effect on the power production. We do not observe the same apparent increase in power output due to turbulence for the northern Turbines 1 and 2 as generally, the wind speeds and turbulence are lower, therefore the effects are less pronounced.

During snow-covered periods, which are not included in our LiDAR measurement period, but are partly represented in the longer time domain of Figure 15b, a general decrease in efficiency is observed over all the turbines. This can likely be explained by the multiple meters thick snow layer covering the mountain pass for 7 months per year smoothing out the terrain, reducing the apparent power increase by turbulence. The high performance of for example Turbine 3 and 5 is therefore reduced the greatest amount for northerlies, as it experienced the greatest discrepancies in summer time. There may be additional factors such as the presence of a much more stable atmospheric boundary layer due to the greatly reduced turbulent heat flux over the snow, resulting in more vertically stratified wind profiles that show more shear and veer and less turbulent airflow incident on the turbines. However, there is currently no data available to validate these speculations quantitatively.

### Sequential LiDAR vs Synchronised LiDAR

The sequential LiDAR and synchronised LiDAR methods are both designed to provide three-dimensional wind fields over study areas. But their logistical and financial requirements, as well as data output quality differ.

The sequential LiDAR method requires a much smaller equipment budget. However, personnel costs are increased due to the logistical requirement of relocating the LiDAR instrument at regular intervals. Synchronised LiDAR methods require extensive planning in advance of the study as well as advanced knowledge on operating two or more LiDAR instruments in parallel, as well as post-processing the different datasets to form a three-dimensional wind field time series. The sequential LiDAR method requires less pre-campaign planning, and data post-processing.

Synchronised LiDAR systems can reach greater spatial resolution by employing multiple LiDARs, as well as extending the measured wind field to a greater domain, however it is still limited by the laser stare-time that is required to gain enough return photons to calculate a Doppler wind speed. Since wind energy assessments are generally based on 10-min average wind speeds, the state-of-the-art synchronised LiDAR systems can measure up to 300 unique points during this time interval ^46^. For the twenty range gates used in this study, such a synchronised LiDAR system comprising of three LiDARs could perform the transect measurements with 15 virtual sequential LiDAR profiles, whilst keeping much greater data availability as no matching algorithm is required.

## Conclusion

This work presents a novel method for high-resolution three-dimensional wind profile measurements in complex terrain using a sequential spatially distributed LiDAR deployment. The project took place in the Gotthard wind park during the summer of 2023. The sequential LiDAR deployment is made along a transect on the western side of the upper plateau of the Gotthard Pass at an elevation of around 2100 m a.s.l., using a profiling VAISALA WindCube V2.1 wind-Doppler LiDAR instrument. The transect of nine sequential LiDAR locations and an additional tenth deployment on the eastern side of the pass aims to probe the entire wind park closely following the sites of five Enercon E92 wind turbines, constituting the high alpine Gotthard wind park. The Gotthard Pass is located in the centre of the Alps and features many topographical effects and flow features typical of complex terrain that influence the wind profile that is incident on the wind turbines and which also can affect the performance of the latter. It is one of the few alpine passes that experiences both northerly and southerly Föhn winds, and wind channelling and acceleration over the pass is observed strongly as can be seen from the measured sequential LiDAR wind profiles.

A matching algorithm is proposed (Section 4) to allow for inter-comparison of LiDAR wind profiles measured at different moments in time, but for similar incoming wind conditions, verified by the meteorological observations on the nacelles of the five turbines. This matching algorithm is successful in identifying a large number of similar wind profiles throughout the three-month field campaign, with a maximum of seven wind profiles measured at different locations. The matching algorithm is independent of the location of the LiDAR wind profile, therefore the robustness of the algorithm has been verified by a statistical analysis of matching wind profiles at the same location, eliminating this unknown factor. With the statistical analysis, it can be concluded that the matching algorithm creates significant matches based on the incoming wind profiles within the error margin of 0.5 m/s in the wind velocity as specified, and therefore it can be used reliably to compare with LiDAR profiles from any other location in the sequential LiDAR deployment.

The sequential LiDAR data provides a unique visualisation of wind shear and wind veer along the alpine pass, as well as turbulence profiles clearly illustrating the complex terrain influences that impact wind turbine performance in mountainous applications (Sections 3, 5). We provide a qualitative overview of the complex terrain effects that significantly influence wind turbine performance, both in terms of potential drawbacks and benefits. Particularly, the stronger northerly winds exhibit acceleration over the pass, increasing wind energy production greatly for turbines furthest downwind. For upwind turbines, wind veer and shear decrease turbine performance, but this effect is reduced further downwind, where the channelling of the narrow pass plateau reduces these effects. The turbulence is largest on the eastern side of the pass, independent of the wind direction, which can be partly explained by the local terrain being rougher compared to the western part of the plateau. The presence of turbulence causes an apparent increase in wind turbine efficiency, which can actually be attributed to kinetic energy in the wind being transferred to turbulence instead of the average wind flow. The nacelle mounted anemometer only measures the average wind speed, but misses out on the short-term wind speed deviations which have a non-linear positive effect on the energy production of the turbines. A qualitative assessment of the compound effects of the complex terrain on the incident wind agrees well with the turbine data.

We hypothesize on effects during the typically 7 months lasting snow-covered winter period in the Gotthard wind park, where the thick snow pack smooths the terrain and reduces turbulence, which turbine data seems to indicate. In future work, we plan to explore in more detail the effect of the presence of a smooth snow pack, which covers the wind park for 7 months of the year, as well as more complex interactions between air density and atmospheric stability outside of the summer period, and how this influences wind turbine performance. The current measurements in the region lack consistent and reliable atmospheric variables, requiring improved sensor coverage for this purpose.

## Ethics and consent

Ethical approval and consent were not required.

## Data Availability

All data collected during the Gotthard experimental campaign are publicly available via Zenodo : Sequential Wind-Doppler LiDAR wind profile measurements on the Gotthard pass in Switzerland – Summer 2023, DOI: 10.5281/zenodo.14524723 ^47^. This project contains the following underlying data: Wind-Doppler LiDAR measurements along the transect in the Gotthard Wind Park. This project contains the following underlying data: Gotthard_Transect_Experiment_STA_standard_Final.nc Opening_netCDF_4_formats.docx Data are available under the terms of the Creative Commons Attribution 4.0 International. The authors have extracted weather data from MeteoSwiss and WSL SLF IMIS weather stations which are available upon request to the respective institutions. The wind turbine data made available by Azienda Elettrica Ticinise (AET) was shared with the authors under non-disclosure agreement for this research project. One dataset that was used in our study has time-series data of wind power, wind speed and control algorithms of the wind turbines. This data was disclosed to the laboratory only under NDA. Readers, with interest for this data, can make a special request to the corresponding author after which the company will consider whether they will share this data under similar NDA conditions.

## References

[1] Elgendi M AlMallahi M Abdelkhalig A : A review of wind turbines in complex terrain. *Int J Thermofluids.* 2023;17: 100289. 10.1016/j.ijft.2023.100289

[2] Hyvärinen A Segalini A : Effects from complex terrain on wind-turbine performance. *J Energy Resour Technol.* 2017;139(5): 051205. 10.1115/1.4036048

[3] Swiss Federal Office for Energy: Energy strategy 2050 once the new energy act is in force.2018. Reference Source

[4] Meyer L Koller S Froidevaux P : Windpotenzial Schweiz 2022: Schlussberich zum Windpotenzial Schweiz 2022. Bern, August,2022. Reference Source

[5] Qosja S Rolle R Gebremedhin A : Solving the bottleneck issue of energy supply. Case study of a wind power plant. *Int J Web Eng Technol.* 2022;5(2):874–891. 10.15157/IJITIS.2022.5.2.874-891

[6] Spiess H Lobsiger-Kägi E Carabias-Hütter V : Future acceptance of wind energy production: exploring future local acceptance of wind energy production in a Swiss Alpine Region. *Technol Forecast Soc Change.* 2015;101:263–274. 10.1016/j.techfore.2015.06.042

[7] Vignali S Lörcher F Hegglin D : A predictive flight-altitude model for avoiding future conflicts between an emblematic raptor and wind energy development in the Swiss Alps. *R Soc Open Sci.* 2022;9(2): 211041. 10.1098/rsos.211041 35154790 PMC8826134

[8] Apostol D Palmer J Pasqualetti M : The renewable energy landscape: preserving scenic values in our sustainable future.Routledge,2016. 10.4324/9781315618463

[9] Bradley S Strehz A Emeis S : Remote sensing winds in complex terrain - a review. *Meteorologische Zeitschrift.* 2015;24(6):547–555. 10.1127/metz/2015/0640

[10] Lange J Mann J Berg J : For wind turbines in complex terrain, the devil is in the detail. *Environ Res Lett.* 2017;12(9): 094020. 10.1088/1748-9326/aa81db

[11] Bechmann A Sørensen NN Berg J : The bolund experiment, part II: blind comparison of microscale flow models. *Boundary-Layer Meteorol.* 2011;141(2):245–271. 10.1007/s10546-011-9637-x

[12] Berg J Mann J Bechmann A : The bolund experiment, part I: flow over a steep, three-dimensional hill. *Boundary-Layer Meteorol.* 2011;141(2):219–243. 10.1007/s10546-011-9636-y

[13] Vassberg JC Tinoco EN Mani M : Abridged summary of the third AIAA computational fluid dynamics drag prediction workshop. *J Aircraft.* 2008;45(3):781–798. 10.2514/1.30572

[14] Menke R Vasiljević N Mann J : Characterization of flow recirculation zones at the Perdigão site using multi-lidar measurements. *Atmos Chem Phys.* 2019;19(4):2713–2723. 10.5194/acp-19-2713-2019

[15] Wildmann N Kigle S Gerz T : Coplanar lidar measurement of a single wind energy converter wake in distinct atmospheric stability regimes at the Perdigão 2017 experiment. *J Phys Conf Ser.* 2018;1037(5): 052006. 10.1088/1742-6596/1037/5/052006

[16] Wenz F Langner J Lutz T : Impact of the wind field at the complex-terrain site perdigão on the surface pressure fluctuations of a wind turbine. *Wind Energy Sci.* 2022;7(3):1321–1340. 10.5194/wes-7-1321-2022

[17] Venkatraman K Hågbo TO Buckingham S : Effect of different source terms and inflow direction in atmospheric boundary modeling over the complex terrain site of Perdigão. *Wind Energy Sci.* 2023;8(1):85–108. 10.5194/wes-8-85-2023

[18] Wagner J Gerz T Wildmann N : Long-term simulation of the boundary layer flow over the double-ridge site during the Perdigão 2017 field campaign. *Atmos Chem Phys.* 2019;19(2):1129–1146. 10.5194/acp-19-1129-2019

[19] Barthelmie RJ Pryor SC Wildmann N : Wind turbine wake characterization in complex terrain via integrated Doppler lidar data from the Perdigão experiment. *J Phys Conf Ser.* 2018;1037(5): 052022. 10.1088/1742-6596/1037/5/052022

[20] Barthelmie RJ Pryor SC : Impact of local meteorology on wake characteristics at Perdigão. *J Phys Conf Ser.* 2019;1256(1):012007. 10.1088/1742-6596/1256/1/012007

[21] Volkert H Gutermann T : Inter-domain cooperation for mesoscale atmospheric laboratories: the mesoscale alpine programme as a rich study case. *Quart J Royal Meteorol Soc.* 2007;133(625):949–967. 10.1002/qj.95

[22] Flamant C Richard E Schär C : The wake south of the Alps: dynamics and structure of the lee-side flox and secondary potential vorticity banners. *Quart J Royal Meteorol Soc.* 2004;130(599):1275–1303. 10.1256/qj.03.17

[23] Schär C Sprenger M Lüthi D : Structure and dynamics of an Alpine potential-vorticity banner. *Quart J Royal Meteorol Soc.* 2003;129(588):825–855. 10.1256/qj.02.47

[24] Walser A Schär C : Convection-resolving precipitation forecasting and its predictability in Alpine river catchments. *Journal of Hydrology.* 2004;288(1–2):57–73. 10.1016/j.jhydrol.2003.11.035

[25] Walser A Lüthi D Schär C : Predictability of precipitation in a cloud-resolving model. *Mon Weather Rev.* 2004;132(2):560–577. 10.1175/1520-0493(2004)132<0560:POPIAC>2.0.CO;2

[26] Mackenzie H Dyson J : Short term forecasting of wind power plant generation for system stability and provision of ancillary services. *Wind Integration Forum Proceedings.* 2017;16. Reference Source

[27] Risan A Lund JA Chang CY : Wind in complex terrain—lidar measurements for evaluation of CFD simulations. *Remote Sensing.* 2018;10(1):59. 10.3390/rs10010059

[28] Bingöl F Mann J Foussekis D : Conically scanning lidar error in complex terrain. *Meteorologische Zeitschrift.* 2009;18(2):189–195. 10.1127/0941-2948/2009/0368

[29] Wagner R Bejdic J : WINDCUBE+ FCR test at Hrgud, Bosnia and Herzegovina. 2014. Reference Source

[30] Kristianti F Dujardin J Gerber F : Combining weather station data and short-term LiDAR deployment to estimate wind energy potential with machine learning: a case study from the swiss alps. *Boundary-Layer Meteorol.* 2023;188(1):185–208. 10.1007/s10546-023-00808-y

[31] Dujardin J Lehning M : Wind-topo: downscaling near-surface wind fields to high-resolution topography in highly complex terrain with deep learning. *Q J R Meteorol Soc.* 2022;148(744):1368–1388. 10.1002/qj.4265

[32] Melani PF Di Pietro F Motta M : A critical analysis of the uncertainty in the production estimation of wind parks in complex terrains. *Renew Sustain Energy Rev.* 2023;181: 113339. 10.1016/j.rser.2023.113339

[33] Baker WE Atlas R Cardinali C : Lidar-measured wind profiles: the missing link in the global observing system. *Bull Am Meteorol Soc.* 2014;95(4):543–564. 10.1175/BAMS-D-12-00164.1

[34] Panofsky HA Ming Z : Characteristics of wind profiles over complex terrain. *Journal of Wind Engineering and Industrial Aerodynamics.* 1983;15(1–3):177–183. 10.1016/0167-6105(83)90188-5

[35] Clifton A Clive P Gottschall J : IEA wind task 32: wind lidar identifying and mitigating barriers to the adoption of wind lidar. *Remote Sens.* 2018;10(3):406. 10.3390/rs10030406

[36] Clifton A Barber S Stökl A : Research challenges and needs for the deployment of wind energy in hilly and mountainous regions. *Wind Energ Sci.* 2022;7(6):2231–2254. 10.5194/wes-7-2231-2022

[37] Fuertes FC Iungo GV Porté-Agel F : 3D turbulence measurements using three synchronous wind lidars: validation against sonic anemometry. *J Atmos Ocean Technol.* 2014;31(7):1549–1556. 10.1175/JTECH-D-13-00206.1

[38] Boquet M Thobois L : Wind resource assessment campaign with lidars and met mast in large and complex sites.In: *EGU general assembly conference abstracts.* 2012;14:12718. Reference Source

[39] Lang S McKeogh E : LIDAR and SODAR measurements of wind speed and direction in upland terrain for wind energy purposes. *Remote Sens.* 2011;3(9):1871–1901. 10.3390/rs3091871

[40] Barthelmie RJ Folkerts L Ormel FT : Offshore wind turbine wakes measured by sodar. *J Atmos Ocean Technol.* 2003;20(4):466–477. 10.1175/1520-0426(2003)20<466:OWTWMB>2.0.CO;2

[41] Crescenti GH : A look back on two decades of doppler sodar comparison studies. *Bull Am Meteorol Soc.* 1997;78(4):651–674. 10.1175/1520-0477(1997)078<0651:ALBOTD>2.0.CO;2

[42] SLF: Description of automated stations — slf.ch. 2024. Reference Source

[43] MeteoSwiss: Measurement values and measuring networks - MeteoSwiss — meteoswiss.admin.ch. 2024. Reference Source

[44] Kröpfli D Schlegel T Geissmann M : Windatlas schweiz: Jahresmittel der modellierten windgeschwindigkeit und windrichtung.Bundesamt für Energie BFE Oct.2022. Reference Source

[45] MeteoSwiss: Föhnindex - MeteoSchweiz. 2024. Reference Source

[46] HALO PHOTONICS | StreamLine series - Product — halo-photonics.com. Reference Source

[47] van Schaik B Lehning M Huwald H : Sequential wind-doppler lidar wind profile measurements on the Gotthard pass in Switzerland - Summer 2023. *Open Research Europe, Zenodo.* 2024. 10.5281/zenodo.14524723 PMC13058581 41960566

