# Resolving three-dimensional wind velocity fields in complex terrain using sequential wind-Doppler LiDAR, CFD and wind turbine measurements - Gotthard Pass, Switzerland

**DOI:** 10.12688/openreseurope.19095.3

**Published:** 2026-03-23

**Authors:** Brandon van Schaik, Dylan Reynolds, Hendrik Huwald, Michael Lehning

**Affiliations:** 1 Laboratory of Cryospheric Sciences, Environmental Engineering Institute, Ecole polytechnique federale de Lausanne (EPFL), Sion, Valais, 1950, Switzerland; 2 Snow Processes, Swiss Institute for Snow and Avalanche Research (SLF), Davos, Graubünden, 7260, Switzerland

**Keywords:** wind, energy, complex terrain, lidar, wind turbine, shear, turbulence, turbulence kinetic energy, wind turbine efficiency, wind channeling, stratification, atmosphere, Gotthard, Switzerland

## Abstract

Background Understanding the effects of complex terrain on wind turbines in alpine regions requires high-resolution computational modelling accompanied by detailed wind observations. In technologically advanced measurement campaigns, often multiple synchronised wind-Doppler LiDARs are deployed to overcome the limitation of these instruments to only measure line-of-sight velocity. Methods In this work, a novel deployment method, a sequential wind-Doppler LiDAR deployment is introduced. We present the example of a field campaign on the Gotthard Pass, a narrow north-south permutated high-mountain pass in the central Swiss Alps. We propose a matching algorithm that can robustly group wind profiles, enabling comparable scientific detail to study turbine efficiency as in synchronised triple LiDAR campaigns, whilst only requiring a single LiDAR instrument to be deployed. Results In the three-month study period in the summer of 2023, we successfully used turbulence kinetic energy, wind shear and veer, as well as wind channelling to explain turbine power production discrepancies observed in the five turbines erected on a mountain pass.

## Plain Language Summary

We have developed a new method of using an existing measurement device to improve the information we can gather about the changes in the wind as they pass over a mountain pass. Five wind turbines were constructed in 2020 on top of a prominent Swiss mountain pass in the Gotthard Wind Park. It is known that these mountain winds are much stronger due to an effect called wind channelling, allowing the wind turbines to generate more energy. With our new measurement method, we can visualise the airflow through the wind park with just one sensor by applying an algorithm that can identify similar wind conditions at different times. Our measurement campaign during the Summer of 2023 allowed us to study several different flow patterns and identify some complex flow structures that affect the efficiency of the wind turbine. We show for each turbine, specifically for Northerly and Southerly winds, how efficient their energy production is, and explain with our detailed wind flow measurements, how these can arise due to the surrounding mountains.

## Introduction

Exact and reliable measurements of the three-dimensional wind are paramount for wind energy potential calculations used to design wind farms. Wind measurements in complex terrain are much more challenging than over flat terrain due to the increased complexity of wind patterns both horizontally and vertically. Complex terrain, in a wind energy assessment (WEA) context, refers to an area with varied and intricate orographical features that can significantly impact wind flow patterns. Complex terrain often includes diverse landscape elements such as hills, valleys, ridges, cliffs, boulders, trees, or human infrastructure irregularities such as buildings or wind turbines that can cause complex interactions with the incident wind. ^1^ In WEA, understanding and quantifying the effects of complex terrain is crucial because these features can lead to variations in wind speed, wind direction, and turbulence, influencing the performance of the wind turbine by the non-idealised wind flow into the turbine. ^2^

In Switzerland, the consideration of complex terrain for WEA is particularly pertinent. Policymakers have shown their intentions to phase out nuclear energy sources and transition to a fully renewable energy strategy by 2050, ^3^ which was approved by the public referendum on 21 May 2017. In a 2022 report by the Swiss Federal Office of Energy, it was concluded that nearly half of Swiss wind energy potential is located in the alpine region. ^4^ This amount becomes even more impressive when the issues related to alpine wind energy are highlighted: 1) the logistics of wind turbine construction in the mountains is more challenging and costly, ^5^ ^,^ ^6^ 2) the endangerment of wildlife, and especially bird species can be unacceptable in some regions, ^7^ 3) social acceptance in the form of nature and wilderness zoning, including natural horizon objections can limit wind turbine projects to several kilometres away from settlements and mountain roads or trails. ^6^ ^,^ ^8^ The recent construction of several wind parks in the complex terrain of the Alps emphasises the imperative for further detailed studies in this domain. The present study focuses only on the physical aspects of wind potential assessment, not on ecological and social acceptance issues.

### LiDAR studies in complex terrain

Several extensive field experiments addressing flows in complex terrain have been conducted in the past twenty years. In 2007–2008, the Bolund experiment, ∼25 km East of Copenhagen, Denmark, set the benchmark for experimental complex terrain studies using remote sensing applications such as wind-Doppler Light Detection and Ranging (LiDAR). This small 300 m wide mesa-shaped island with slopes on all aspects exceeding 30° surrounded by a large body of water serves as an idealised example for showcasing the effects of terrain features on the wind. ^9^ The hill was covered with 10 meteorological masts (MET-mast) ranging between 20 and 150 m in height, with a LiDAR on the far end of the peninsula and a LiDAR 300 m away on the leeward side of the hill. Based on the 60-day experimental campaign, flow field simulations were validated to understand the exact flow over the terrain feature up to 150 m above ground level (a.g.l.). Although the Bolund experiment provides the benchmark for simulations ^10^ and data of high-resolution wind profiles ^11^ for WEA, the peninsula does not accurately represent more complex terrain such as alpine environments.

A more advanced complex terrain study in 2015–2017, specifically aimed at WEA, was carried out in Perdigão, ∼150 km northeast of Lisbon, Portugal. ^12^ Here, two parallel hill ridge lines, separated by approx. 1500 m are covered with wind turbines. Over the three years, various measurement campaigns were held including multiple wind-Doppler LiDARs, which were used in conjunction to resolve three-dimensional wind velocity fields throughout the entire valley. ^13^ ^,^ ^14^ Long-term studies of the meso-scale wind profile for WEA have been validated with LiDAR data, ^15^ ^,^ ^16^ as well as much smaller-scale turbine wake simulations, ^17^ ^,^ ^18^ establishing the use of LiDAR measurements as a valid wind assessment tool over a wide range of spatial resolutions.

The largest instances of complex terrain wind studies, such as the Meso-scale Alpine Project (MAP), ^19^ have defined the state-of-the-art for the past 20 years. Although the MAP lacks specific wind energy analyses, the project serves as the basis for the validation of numerical weather prediction models in highly complex terrain. ^20^ ^–^ ^23^

Such large-scale wind energy projects provide a wealth of detailed observational data available for high-resolution simulations of their respective study areas, however not all WEA studies merit and require such large investments. WEAs rarely operate more than one LiDAR to perform a wind energy assessment.

Single LiDAR experiments have proven to be capable of forecasting short-term wind fluctuations, ^24^ as well as suitable for CFD model validation in complex terrain. ^25^ ^–^ ^27^ Kristianti *et al.* ^28^ investigated the complex alpine sites of Les Diablerets and the Lukmanier Pass, both representative of the Swiss Alps, in a comparative study to estimate the wind energy potential at these locations. Here a novel approach pioneered the use of artificial neural networks to combine weather station data with limited wind-Doppler LiDAR measurements to improve WEA. Additionally, the use of a machine learning model, called Wind-Topo, ^29^ which forecasts 10 m a.g.l. wind speeds at 53 m resolution, was coupled to 100 m a.g.l. wind speeds measured by the LiDAR system using a specific transfer function. Although this transfer function could not yet be validated in a general alpine context, it proves the potential for the combination of different data sources to improve complex terrain WEA without increasing investment costs massively.

### State-of-the-art in the industry

The prevalence of terrestrial remote sensing techniques, such as LiDAR experiments, has significantly advanced the ability of WEA in scientific communities. Despite the popularity of LiDAR, conventional MET-masts equipped with anemometers extending up to at least two-thirds of the projected turbine hub height remain the industry standard. This preference is attributed in part to the advantage of obtaining a single-point three-dimensional wind measurement, ^30^ a feature challenging for LiDAR systems, especially in complex terrains where horizontal wind continuity and uniformity cannot be guaranteed within the scanning cone. ^31^ Conversely, the assumption that is made to extrapolate MET-mast measurements from two-thirds of the turbine hub height to a complete profile over the swept area by the turbine blades, usually by logarithmic wind profile theory, cannot be applied to extreme complex terrain locations. ^32^

While the concept of employing multiple LiDARs appears promising in addressing this challenge, practical issues persist in installation, maintenance, data analysis, cost and instrument availability. ^33^ ^–^ ^35^ These complexities demand an advanced understanding of the instruments and the environmental conditions, consequently contributing to elevated operational costs. Moreover, the construction expense, impracticality of installing MET-masts in mountainous regions combined with severe weather and increased risk of natural hazards further underscore the need for alternative approaches. ^36^

In response to these challenges, remote sensing methodologies employing a single wind-Doppler LiDAR have gained traction as a cost-effective and efficient alternative. In certain scenarios, sound-wave-based alternatives like Sonic Detection and Ranging (SoDAR) have been explored, ^37^ ^,^ ^38^ albeit infrequently. This is primarily due to the limitations in range and concerns about noise pollution associated with SoDARs, positioning single LiDAR WEA methodologies as the most logical step forward for prospective wind parks. ^39^

Additionally, an argument can be made for the use of LiDAR profilers instead of free-scanning LiDARs. A LiDAR profiler is designed for measuring vertical atmospheric profiles, emitting laser pulses in fixed directions, often in a conical shape, to capture wind data at a range of altitudes after which it combines the line-of-sight velocities captured in different directions to provide a volume-averaged three-dimensional wind speed of the cone swept by the laser. In contrast, a free-scanning LiDAR can point its laser beam in any direction of a hemisphere following predefined scan patterns, providing more freedom in choosing the line-of-sight wind speeds that are to be measured by the laser beam. This freedom does come at the cost that an experienced user is required to program the suitable scanning patterns and to analyse the data, particularly in complex terrain. The cost of free-scanning LiDARs is nearly three times higher than that of a LiDAR profiler further limiting the incentive for prospective investors to choose this option. Additionally, the free-scanning LiDARs are heavier, more delicate, and have higher power demands, which drastically limits their range of deployment in complex terrain.

### The Gotthard sequential LiDAR transect experiment

It is for these reasons that in this study, we introduce a novel approach employing a *sequential LiDAR* transect experiment utilising a single wind-Doppler LiDAR profiler. The applicability of this methodology is demonstrated in a specific case study focused on a wind farm located on the Gotthard Pass (46.55°N, 8.56°E, 2106 m a.s.l.) connecting the northern and southern alpine regions in Switzerland. Since October 2020, the upper plateau of the pass has been home to five wind turbines, forming the basis of our investigation and providing wind energy data for validation. The Gotthard Wind Park can be considered as the highest degree of complex terrain for wind energy development, characterised by large elevation changes, up- and downwind valleys and steep escarpments within the wind park itself.

This work serves as a proof of concept and as a preliminary showcase of the unique data sets and analyses that are unlocked by the sequential LiDAR experimental methodology, enabling a high-resolution study of the three-dimensional wind field over the entire wind park domain. In addition, we introduce simulated flow fields from the High-resolution Intermediate Complexity Atmospheric Research (HICAR v1.1) model ^40^ which can resolve terrain-induced flow features at the hectometre scale.

This work intends to demonstrate a novel and resource-efficient method of capturing high-resolution wind field data in complex alpine terrain using a sequential deployment of a single wind-Doppler LiDAR. We supplement this by synoptic weather data, flow field simulations and the continuous wind data from the five wind turbines in the Gotthard wind park. Lastly, we eliminate the spatio-temporal discontinuity of the deployment method by matching different time intervals where the flow over the pass can be considered the same.

In section 2, we provide a detailed description of the Gotthard Pass environment and the wind park site, emphasising the existing and additionally installed study-specific instrumentation used. Section 3 outlines the methodology of the sequential LiDAR transect experiment, and proposes a novel method to analyse sequential LiDAR experiments supplemented by simulations. The results, in section 4, describes the unique wind flow over the sequential LiDAR transect including the site-specific and temporal evolution of wind profiles over complex terrain for the Gotthard wind park, exploring the effects of complex terrain on the airflow in the wind park and how this affects turbine performance. Section 5 discusses key findings and the efficacy of the sequential LiDAR method in view of logistical challenges, providing an outlook into the implications for future WEA studies.

## Field site, instrumentation and modelling

**Figure 1.**
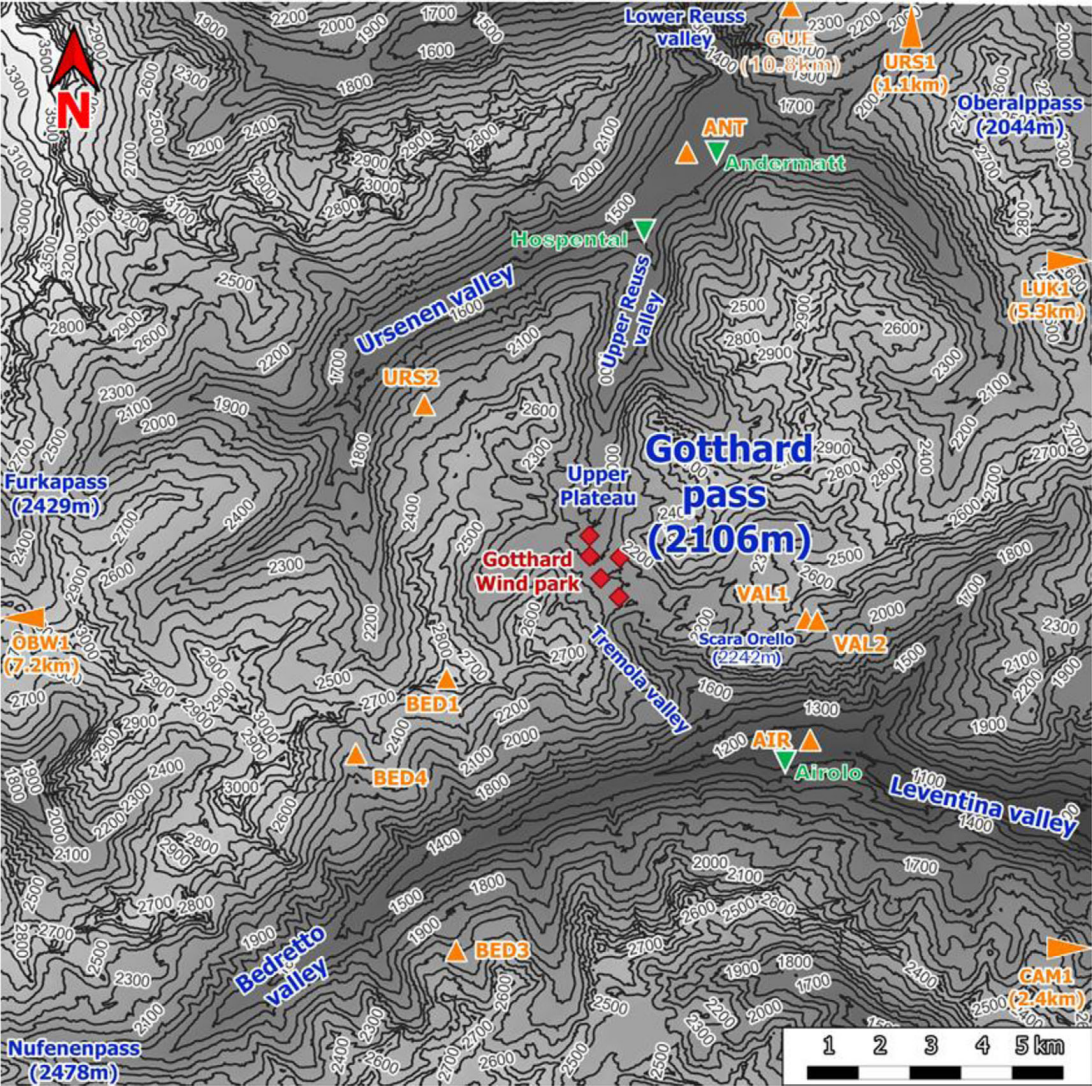
Topographical overview of the Gotthard region.

The Gotthard wind park is situated atop the Gotthard Pass at an elevation of 2106 m above mean sea level (a.m.s.l.) in the Swiss Alps, at approximately 46.55°N latitude and 8.56°E longitude. Figure 1 shows the Gotthard region with 100 m elevation contour lines, the Gotthard wind park turbines (red diamonds), the topographical points of interest (blue), towns (green triangles) and the surrounding weather stations (orange triangles). This pass is a saddle point between the Northern Urseren valley from the town of Andermatt, canton Uri (approximately 1440 m a.s.l.) and the Southern Bedretto valley at the town of Airolo, canton Ticino (approximately 1210 m a.s.l.). From the Urseren valley, the pass ascends over a distance of 6.8 km along a near-north facing slope with an aspect of 7° up to the summit of the Gotthard Pass. This Upper Reuss valley is characterised by an average slope of 5.6° with minimal deviation and few obstacles to obstruct the wind flow. Subsequently, the pass descends over a distance of 4.3 km along a South-East facing slope with an aspect of 147° called the Tremola valley, featuring an average slope of 12.1°. From the village of Hospental to Airolo, the pass curves gradually towards the East with a radius of curvature of 7.5 km.

The central location of the Gotthard Pass is most frequently a major weather divide for the Northern and Southern Alps. Its north-south orientation provides its distinctive and characteristic feature of exhibiting both North and South Föhn winds, thus often experiencing increased wind flow on the respective lee side of the pass and increased wind energy potential. ^28^

**Figure 2.**
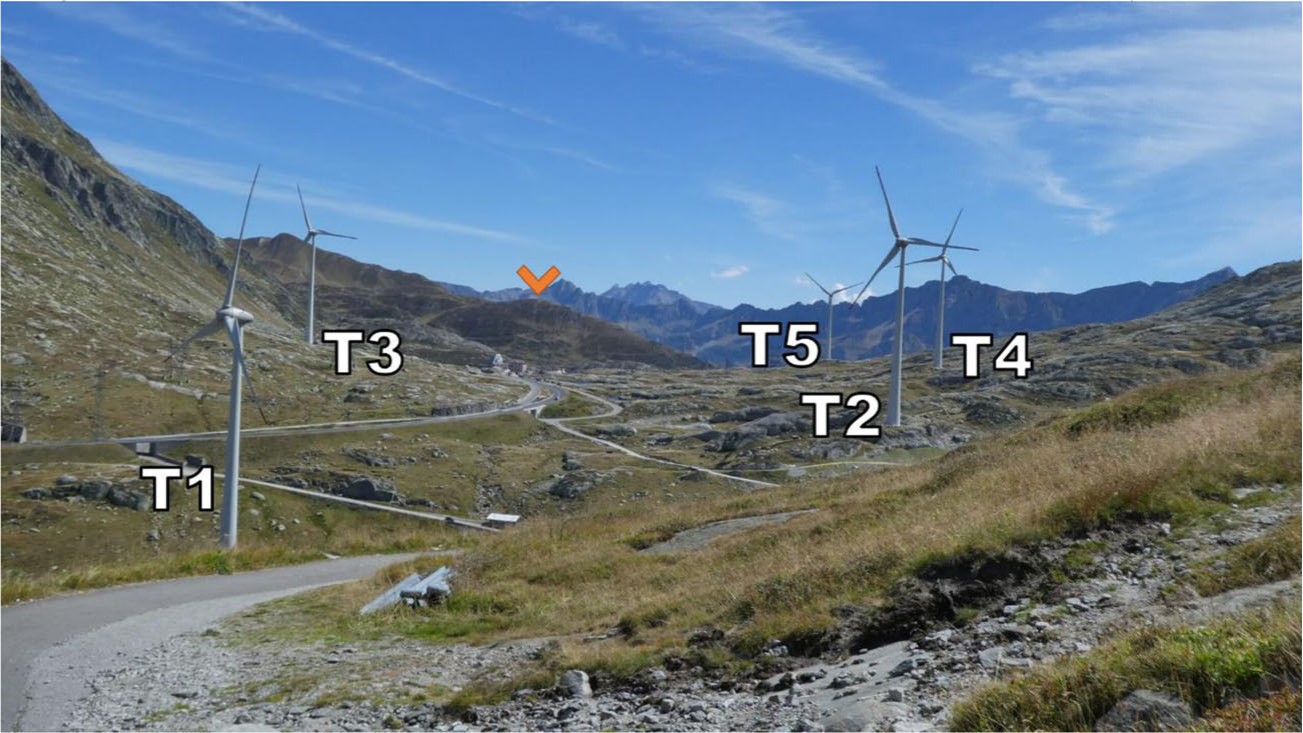
The Gotthard Pass in summer seen from the north-western side of the Upper Plateau.

Continuing with Figure 1, the contours of the mountain pass are indicated. The southern valley, colloquially known as the Tremola valley, is notably narrower compared to its northern counterpart, with a transverse distance of approximately 300 m at an elevation of 100 m above the valley floor. In contrast, the Northern valley is wider with a valley width of 500–750 m at the 100 m above the valley floor. Moreover, the Tremola valley showcases an orographic prominence, indicated on Figure 2 (orange marker), the Scara Orello mountain (2242 m a.m.s.l.), located 2 km southeast of the upper plateau, blocking the free-flowing streamline of air over the eastern side of the pass in southerly winds. This situation will be discussed in more detail later. To the East and the West of the saddle point, prominent mountains reaching up several hundred meters above the upper plateau shelter the wind park from easterly and westerly winds, in fact, they are responsible for the flow channelling customarily observed in the Gotthard wind park.

**Figure 3.**
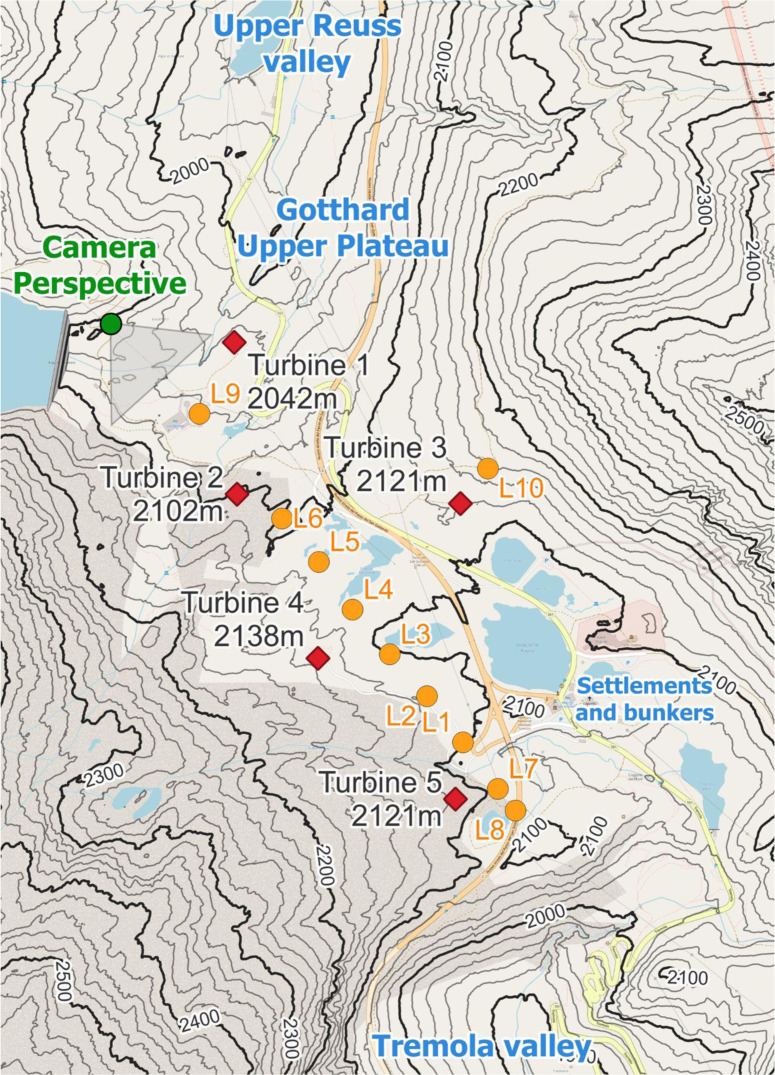
The Gotthard Upper Plateau, featuring wind turbines (red diamonds), LiDAR locations (orange dots), and the camera perspective of Figure 2.

Zooming in on the Gotthard wind park itself ( Figure 3), the Gotthard wind park comprises five turbines positioned on the upper plateau. These turbines are numbered sequentially from North to South, with Turbines 1, 2, 4, and 5 situated on the western side, while Turbine 3 occupies the eastern side. The surrounding landscape features boulders of up to 15 m tall, several small lakes, and grass patches as visible in Figure 2. Notably, the treeline is located far below the upper plateau and thus does not significantly influence the wind flow in the wind park itself. To the Southeast of Turbine 3, a small settlement consisting of low buildings and shelters is observed, exhibiting a similar surface roughness to the boulders present elsewhere on the upper plateau by estimate.

### Instrumentation overview

The five wind turbines in the Gotthard wind park are Enercon E92 turbines with a rotor diameter of 92 m mounted upon a 98 m mast. They are specified for 2.35MW rated power, and a cut-in, rated, and cut-out wind speed of 2 *ms* ^–1^, 11 *ms* ^–1^ and 25 *ms* ^–1^, respectively. Table 1 provides details regarding the geographical coordinates, elevation, and characteristics of these turbines, as well as all instrumentation employed in this study. Firstly, the sequential LiDAR data set includes wind profiles at twenty range gates focused around the turbine height of the wind park, starting with range gates at 40 m up to 160 m increasing by steps of 10 m. After which, the range gates increase by steps of 20 m up to 300 m, providing a comprehensive profile of the wind park by including wind speeds well above the turbines. A range gate is a specific range interval from the LiDAR instrument within which it measures the wind speed and direction by analysing the Doppler shift of the returned signal within this interval.

**Table 1.** Instrumentation and data availability. Easting and Northing coordinates in the Swiss Coordinate system LV95 (EPSG:2056).

Dataset	Station	Data access	Easting [m]	Northing [m]	Altitude [m]	Period [ISO 8601, UTC]	Description
**LiDAR transect**	L1	Available via Envidat	2686269	1156520	2101	2023-06-19T14:20Z - 2023-06-21T20:50Z	Origin of transect
	L2		2686173	1156369	2116	2023-07-14T11:10Z - 2023-07-14T11:00Z	+150m along transect
	L3		2686074	1156747	2103	2023-07-14T11:20Z - 2023-07-21T08:50Z	+300m along transect
	L4		2685975	1156862	2110	2023-07-21T13:00Z - 2023-07-27T09:40Z	+450m along transect
	L5		2685885	1156869	2112	2023-07-27T15:50Z - 2023-08-08T11:00Z	+600m along transect
	L6		2685787	1157097	2097	2023-08-11T12:10Z - 2023-08-12T09:10Z	+750m along transect
	L7		2686362	1157400	2096	2023-08-18T14:20Z - 2023-08-29T09:10Z	-150m along transect
	L8		2686409	1156444	2089	2023-08-29T10:40Z - 2023-09-04T07:00Z	-225m along transect
	L9		2685567	1157235	2063	2023-09-04T15:50Z - 2023-09-12T06:30Z	+1100m along transect
	L10		2686324	1157325	2157	2023-09-12T11:00Z - 2023-09-20T00:00Z	Off transect near Turbine 3
**Wind Turbines (AET)**	T1	N/A	2685655	1157555	2140	2020-10-29T09:50Z - 2023-09-20T00:00Z	
	T2		2685668	1157159	2200	2020-10-08T16:00Z - 2023-09-20T00:00Z	
	T3		2686253	1157143	2219	2020-10-19T17:30Z - 2023-09-20T00:00Z	On west-facing slope
	T4		2685886	1156734	2236	2020-11-23T11:30Z - 2023-09-20T00:00Z	
	T5		2686251	1156372	2219	2020-11-16T17:40Z - 2023-09-20T00:00Z	
**Weather stations (SLF)**	BED1	Publicly available by request to WSL SLF IMIS	2682908	1154748	2963	2023-06-19T00:00Z - 2023-09-20T00:00Z	Mountain top (West)
	BED3		2683169	1149445	2101	2023-06-19T00:00Z - 2023-09-20T00:00Z	Bedretto valley (North facing)
	BED4		2681160	1153250	2305	2023-06-19T00:00Z - 2023-09-20T00:00Z	Bedretto valley (South facing)
	CAM1		2696885	1148709	2668	2023-06-19T00:00Z - 2023-09-20T00:00Z	Leventina valley (North facing)
	GOS3		2689425	1167908	2209	2023-06-19T00:00Z - 2023-09-20T00:00Z	Mountain ridge (North)
	LUK1		2703254	1163181	3040	2023-06-19T00:00Z - 2023-09-20T00:00Z	Mountain top (East, far)
	OBW1		2666706	1155765	2733	2023-06-19T00:00Z - 2023-09-20T00:00Z	Mountain top (West, far)
	URS1		2691804	1169246	2784	2023-06-19T00:00Z - 2023-09-20T00:00Z	Mountain top (North)
	URS2		2682405	1160066	2169	2023-06-19T00:00Z - 2023-09-20T00:00Z	Urseren valley (North facing)
	VAL1		2689900	1156001	2450	2023-06-19T00:00Z - 2023-09-20T00:00Z	Tremola valley (Eastern mountain top)
	VAL2		2690126	1155980	2628	2023-06-19T00:00Z - 2023-09-20T00:00Z	Tremola valley (South facing)
**Weather stations (MeteoSwiss)**	ANT	Pubicly available by request to MeteoSwiss	2687445	1165044	1435	2023-06-19T00:00Z - 2023-09-20T00:00Z	North exit of Gotthard pass (Andermatt)
	AIR		2690019	1153645	1157	2023-06-19T00:00Z - 2023-09-20T00:00Z	South exit of Gotthard pass (Airolo)
	GUE		2690087	1167480	2286	2023-06-19T00:00Z - 2023-09-20T00:00Z	Mountain ridge Gütsch (North, 11km)
**HICAR v1.1**		Data available upon request	Availabe throughout the Gotthard domain 50m horizontal resolution following the LV95 (EPSG:2056) coordinate system.			2023-06-01T00:00Z - 2023-09-22T00:00Z	50m spatial resolution, 32 levels from at [10, 33, 61, 95, 135, 1885, 246, 328m a.g.l., etc.]

This large array of wind profiles is complemented by both wind speed and power metrics from individual turbines, which are made available by the turbine operators, Azienda Elettrica Ticinese. Data from eleven mountain weather stations operated by the Swiss Institute for Snow and Avalanche Research. ^41^ Additionally, three weather stations in the vicinity of the Gotthard Pass and one at a nearby wind park of similar elevation are used, all of which are operated by the Swiss Federal Office for Meteorology and Climatology. ^42^ Finally, the HICAR v1.1 model is employed at a resolution of 50 m for the duration of the campaign and throughout the entire Gotthard domain as indicated in Figure 1.

### LiDAR details

The VAISALA WindCube v2.1 WLS7-1726 profiler was the sole remote-sensing wind measurement instrument used for this campaign, sequentially situated between the five turbines in the Gotthard wind park. The LiDAR profiler makes use of five pulsed laser beams, which are magnetically controlled by the instrument with 0.8 s of exposure in a vertical stare position after the instrument moves the laser in 0.2 s to its next positions: North, East, South, and West with 28° angle from the vertical axis with the same exposure time. The return signal of the 1543 nm, 30 mW mean power laser is recaptured, and based on the Doppler shift of the five line-of-sight laser beams a three-dimensional wind vector is determined for each range gate in the profile.

This specific instrument logs data at two temporal resolutions: one-second, individual beam data, as well as 10-min averaged data, which can be used for both relatively high-level wind profile and shear structure analysis as well as turbulence intensity calculations for WEA purposes. Both time resolution data sets are made available via the instructions in Table 1.

### Sequential LiDAR transect

To analyse the evolution of wind patterns across the complex terrain of the Gotthard wind park, a sequential LiDAR transect was executed. This transect, conducted over three months from 19 June to 20 September 2023, focuses on the upper plateau where the mountains slopes channel the prevailing wind in the downwind direction. It is noted that, despite the overall complex terrain of the Gotthard Pass, the turbines and LiDAR transect are aligned along a topographically channeled plateau. As a consequence, the flow conditions encountered here are less complex than at exposed hilltop or ridge locations, where flow inclination and flow separation may violate the horizontal homogeneity assumption required for ground-based Doppler LiDAR wind retrievals. This represents a limitation of the present study.

The transect includes nine distinct LiDAR locations, positioned at regular increments, plus one additional deployment near Turbine 3 on the eastern side of the plateau. This LiDAR transect aims to provide an extensive collection of wind profiles along the prevailing wind direction axis of the wind park, emphasising high-resolution, three-dimensional wind velocity fields unattainable through conventional single LiDAR deployments.

The LiDAR transect logistics involved the transport of the LiDAR by vehicle over the various wind turbine servicing roads. When the closest point to the sequential LiDAR position was reached, the LiDAR was carried up to 200 m away from infrastructure over rough terrain by two individuals. It was placed on a levelled wooden palette to minimize movement in soft soil and to protect against flooding. Power was accessed through extension cables up to 330 m long, connected to the grid via different wind turbines. The two extremes of the transect were defined by distances of −225 m and +1’100 m from the origin defined at L1, with the former constrained by a highway crossing and the latter avoiding potential wake interference from Turbines 1 and 2.

A radiation-shielded combined pressure, temperature, and humidity sensor was placed near the LiDAR 1 m above the ground which is solely used to calculate the local air density. This sensor provides 10-minute averaged atmospheric variables at the LiDAR site. The LiDAR wind profiles could be monitored remotely via a 4G connection using the LTE network available on the Gotthard Pass, and the data was extracted on a near-weekly basis when the LiDAR was moved to its new position.

In some cases, data gaps are observed within the LiDAR transect which occurred due to disconnection or power loss from the grid from which the LiDAR was powered. In particular, heavy thunderstorms and hail storms which are common in the Swiss high-alpine region were prone to shutting down the LiDAR measurements. Due to long access time to the study area, the LiDAR sometimes could not be serviced promptly after shutdown further extending the data gaps.

The nine sequential LiDAR locations, spaced at 150 m increments, mostly covered the western part of the wind park. The orientation of the transect was based on the prevailing wind direction extracted from the Swiss Wind Atlas providing wind rose information at 100 m above the ground level, at 100 m horizontal and 25 m vertical resolution. ^43^ Additionally, a tenth LiDAR location near Turbine 3 on the eastern side aimed to capture representative wind profiles from the eastern sector.

### Diagnostic wind model HICAR v1.1

To provide a simulated 3D wind field for the spatial and temporal extent of the study, the High-resolution Intermediate Complexity Atmospheric Research (HICAR) model was used. ^40^ HICAR was chosen over an LES model due to its fast runtime, which is orders of magnitude faster than a conventional atmospheric model. This enables simulation of the entire observational period, instead of only a few events. Key to HICAR’s computational efficiency is its use of a diagnostic, incompressible mass-conserving wind solver in place of the full Navier-Stokes equations. The wind solver, first introduced by, ^44^ has seen widespread use and development in the pollutant transport community in the decades since. When solving for the flow field, effects of terrain blocking or channelling are considered depending on the ambient atmospheric stability and obstacle height. In addition to the diagnostic solver, HICAR uses a parameterization for leeside recirculation and slow down. The resultant flow fields have been validated in complex terrain using a wind LiDAR, where ^45^ found that HICAR can resolve flow features induced by terrain, but notably not buoyancy-driven turbulent features.

The HICAR simulations utilized two nests, one at a 1km resolution extending roughly 100km beyond the boundaries of the study domain, and a second at a 50m resolution covering the study domain. The outer domain was forced hourly with reanalysis data from the COSMO1 model, ^46^ which provided operational forecasts over Switzerland until 2024. HICAR was run with the Morrison microphysics scheme, the RRTMG radiation scheme with terrain shading, NoahMP land surface model, and a 3rd order accurate advection scheme. The physics parameterizations affect both the density of the atmosphere and the atmospheric stability, ultimately influencing the diagnostic wind solver.

## Methodology

The primary challenge in analysing sequential LiDAR measurements lies in addressing the inherent lack of spatio-temporal continuity within the data set. As the weather is dynamic throughout the three-month measurement campaign, a method of inter-comparison between the different LiDAR deployments along the transect is developed that evaluates the similarity of atmospheric conditions across time. We expect that similar synoptic wind conditions across the pass when similar wind vectors are measured at all five nacelles in the Gotthard wind park. Firstly, nacelle anemometer data from the turbines is assessed based on vectorial wind speed differences, after which eight characteristic features of the atmosphere and flow features based on the HICAR simulations are considered.

### Nacelle wind data

Firstly, wind velocity is measured on the nacelles of the five turbines situated on the upper plateau. By considering the wind vectors measured at the nacelles of each turbine, similar inflow characteristics can be deduced from which these common time frames can be compared over the entire sequential LiDAR study to show the evolution of wind profiles along the upper plateau.

Nacelle wind velocities are collected by anemometers located at 98 m above ground level. These stations measure average wind speed and direction at 10-minute intervals, but do not include a measure for turbulence intensity. Within the time domain of the sequential LiDAR campaign, the horizontal wind speed components U and V are calculated for the total of 13’295 10-minute intervals covering the entire campaign period.

**Figure 4.**
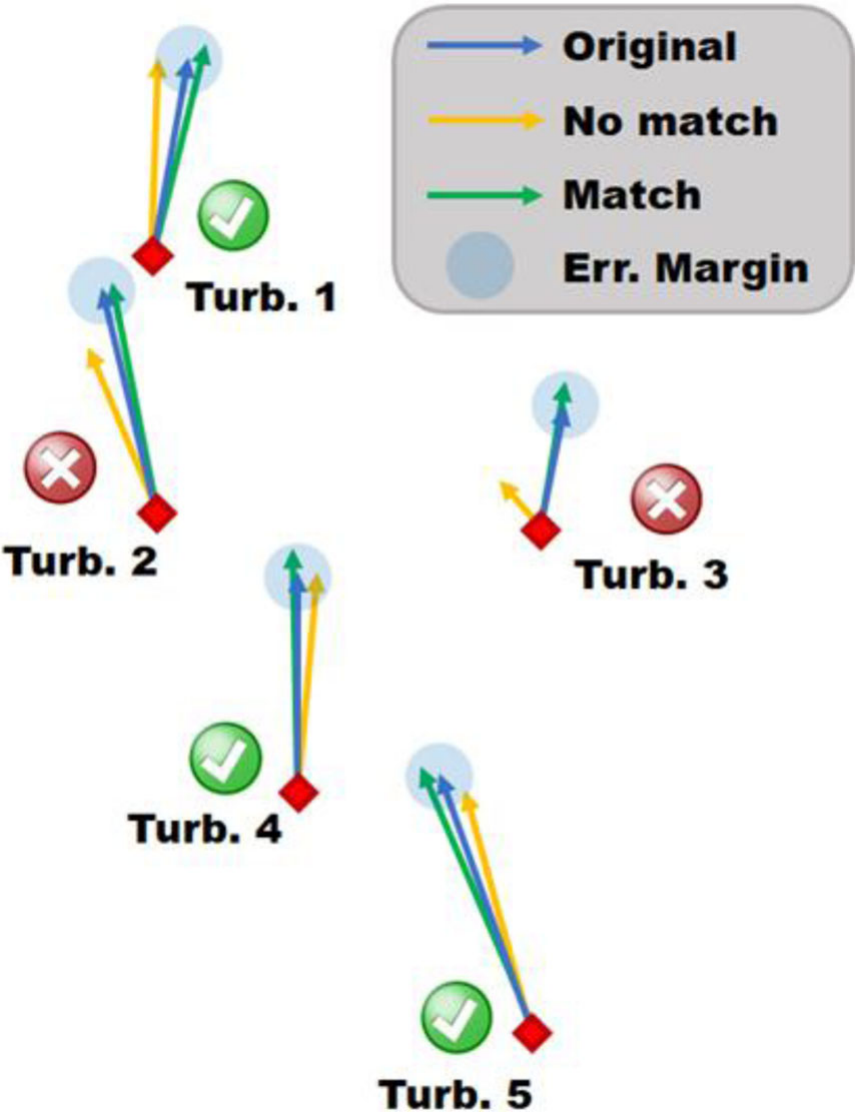
Schematic of the matching algorithm, visualize three different snapshots by coloured vectors.

As shown in Figure 4, the vectorial difference in wind speed for each turbine is evaluated for each pair of 10-minute intervals (i.e., 5 · 13'295 ^2^/2 ≈ 4.4 · 10 ^8^ pairs are evaluated) in the data set (Equation 1).

$$\left[\begin{array}{c} \Delta V_{ij}^{T_{1}}\\ \Delta V_{ij}^{T_{2}}\\ \Delta V_{ij}^{T_{3}}\\ \Delta V_{ij}^{T_{4}}\\ \Delta V_{ij}^{T_{5}} \end{array}\right]=\left[\begin{array}{c} \left|{\vec{v}}_{i}^{T_{1}}-{\vec{v}}_{j}^{T_{1}}\right|\\ \left|{\vec{v}}_{i}^{T_{2}}-{\vec{v}}_{j}^{T_{2}}\right|\\ \left|{\vec{v}}_{i}^{T_{3}}-{\vec{v}}_{j}^{T_{3}}\right|\\ \left|{\vec{v}}_{i}^{T_{4}}-{\vec{v}}_{j}^{T_{4}}\right|\\ \left|{\vec{v}}_{i}^{T_{5}}-{\vec{v}}_{j}^{T_{5}}\right| \end{array}\right]$$ (1)

$$match\;({\vec{v}}_{i}^{L_{x_{1}}},{\vec{v}}_{j}^{L_{x_{2}}})=\max_{T_{x}\in \{T_{1},T_{2},T_{3},T_{4},T_{5}\}}\Delta V_{ij}^{T_{x}}\leq V_{margin}=0.5\;m/s$$ (2)

Where ${\vec{v}}_{i}^{T_{x}}$ and ${\vec{v}}_{j}^{T_{x}}$ [ *ms* ^–1^] are turbine nacelle wind velocities at time interval *i* and *j*, respectively, for turbine *T*_*x*∈{1,2,3,4,5}_. $\Delta V_{ij}^{T_{x}}$ [ *ms* ^–1^] is the vectorial difference in the wind speed. If all five turbines exhibit a wind velocity within the given error margin *V _margin_* = 0.5 *ms* ^–1^ (Equation 2), the pair is considered to be a match. This error margin is chosen to be approximately 10% of the mean wind speed of the wind park, whilst remaining higher than the error specification of the anemometer.

An example, shown in Figure 4, shows that for Turbines 1 and 5, the green and yellow vectors are within the blue error margin but not within the margin of each other. Resulting in the green vectors forming a match with blue, whilst the yellow vectors do not.

Since the matching algorithm described above only requires the wind turbine wind velocity data points, there are several cases where the timestamp does not have a corresponding LiDAR wind profile due to LiDAR data gaps. Approximately 4’000 out of 6’000 matches have LiDAR data available for at least two wind profiles ( ${\vec{v}}_{i}^{L_{x_{1}}}$ & ${\vec{v}}_{j}^{L_{x_{2}}}$ ) from the matched timestamps to make inter-comparison possible (Equation 3).

$$\exists {\vec{v}}_{i}^{L_{x_{1}}},{\vec{v}}_{j}^{L_{x_{2}}}|L_{x_{1}},L_{x_{2}}\in \{L_{1},L_{2},\;...\;,L_{10}\},L_{x_{1}}\neq L_{x_{2}},match({\vec{v}}_{i}^{L_{x_{1}}},{\vec{v}}_{j}^{L_{x_{2}}})$$ (3)

Where *L*_*x*_1__ and *L*_*x*_2__ indicate two unique LiDAR locations, for both of which the LiDAR profiler has measured a wind velocity at the matched time interval *i.* It should be noted that Equation 3 describes only the minimum requirement for a match to be formed, in many cases more than two different LiDAR positions are in the same match, and multiple time intervals can exist for the same LiDAR position (Equation 4). However, such a match for *i* does not necessarily imply that *j* and *k* are also a match with each other, as their wind vectors could potentially be on the other side of the error margin (consider Turbine 1 in Figure 4).

$$match\;({\vec{v}}_{i}^{L_{x_{1}}},{\vec{v}}_{j}^{L_{x_{2}}},{\vec{v}}_{k}^{L_{x_{3}}})\to match\;({\vec{v}}_{i}^{L_{x_{1}}},{\vec{v}}_{j}^{L_{x_{2}}}),match\;({\vec{v}}_{i}^{L_{x_{1}}},{\vec{v}}_{k}^{L_{x_{3}}})$$ (4)

### HICAR characteristic features

Building on the nacelle-based matching, we refine the inter-comparison by introducing eight characteristic parameters derived from the HICAR v1.1 mesoscale model. The model provides wind fields on a 50 m horizontal grid with 32 vertical levels, which were interpolated to the nacelle positions (98 m a.g.l.), and to the ten LiDAR locations with their respective range gates.

From these interpolated fields, along with local measurements, we define eight parameters that characterise the inflow conditions from the Gotthard wind park domain, which is chosen to be around Turbine 4 as it is the most central turbine:

(1) Global horizontal irradiation ( *GHI* [ *Wm* ^–2^]), a direct output of HICAR from the grid cell of Turbine 4.

(2) Atmospheric stability $(\frac{\partial \theta }{\partial z}[Km^{-1}]),$ calculated from the potential temperature gradient from 10 m to 247 m a.g.l. (7 levels) at the Turbine 4 grid cell.

(3) Vertical shear magnitude $\left(\left|\frac{\partial \vec{v}}{\partial z}\right|[ms^{-1}]\right),$ derived from the horizontal wind vectors from 10 m to 247 m a.g.l., spatially averaged over the transect spanning 2 km.

(4) Variance of the vertical turbulence ( *W*' ^2^ [ *m* ^2^ *s* ^–2^]), derived the variance of the vertical wind from 10 m to 247 m a.g.l., spatially averaged over the transect spanning 2 km.

(5–6) Synoptic wind components ( *U _synop_* & *V _synop_* [ *ms* ^–1^]) derived from the 5 km level of HICAR (level 20) from the grid cell of Turbine 4.

(7–8) Transect-averaged wind components ( *U _transect_* & *V _transect_* [ *ms* ^–1^]) derived from level 3–5 (61–136m a.g.l.) of HICAR.

Each parameter is subsequently standardised by z-scaling to zero mean and unit variance. We are unable to compare the HICAR wind vectors at the wind turbine nacelles with the in-situ data measured by the anemometers due to the flow distortion of the nacelle which is not simulated.

Analogous to the method applied for matching turbine nacelle wind data, we proceed with filtering the matches further using these eight characteristic features by examining their coherence across multiple wind profiles. As this filtering will reduce in size the majority of nacelle data, we only consider combinations of at least 5 different LiDAR wind profiles from distinct locations (90 matches in total).

For each combination, we calculate the standard deviation across all eight characteristic features simultaneously. As a baseline, random selections of 5 to 27 profiles yield standard deviations that are consistently high, with standard deviation values typically between 0.926 ± 0.053 and 0.983 ± 0.015 for 5 to 27 randomly selected wind profiles, respectively.

To identify profiles that are meaningfully related, we define a threshold standard deviation of 0.6 across all eight features. Combinations where the standard deviation for all features falls below this threshold are considered a match, indicating strong coherence between the selected profiles. This approach is analogous to the wind turbine analysis, where a deviation below 0.5 m s ^–1^ in nacelle wind measurements is used to define a valid match.

This standard deviation threshold of *σ* < 0.6 is chosen based on the combined likelihood of a random selection fulfilling these criteria, with the exception of atmospheric stability for which all timestamps should exhibit the same class (stable, neutral, unstable). All matching criteria are summarised in Table 2. By testing over a large number of combinations (10 ^7^ random selections for 5 and 27 wind profiles) we estimate that the probability of a random selection satisfying this strict requirement corresponds to between 3.0 *σ* (< 0.28%) and 5.3 *σ* (<10 ^–7^) for the matches made with the nacelle wind data. Which is statistically unlikely to occur randomly, thus concluding that different previously matched wind profiles passing these strict criteria, are indeed sufficiently related in both wind pattern as well as atmospheric conditions.

**Table 2.** Threshold criteria that are required for the matching algorithm.

Feature	Threshold
Turbine nacelle wind speed (98m a.g.l.)	$\Delta V_{ij}^{T_{x}}$ ≤ 0.5 *ms* ^–1^
Global horizontal Irradiation	*σGHI* ≤ 186 *Wm* ^–2^
Atmospheric stability (10-247m a.g.l.)	Same class: Stable ( $\frac{\partial \theta }{\partial z}$ > 0.002 *Km* ^–1^), Unstable ( $\frac{\partial \theta }{\partial z}$ ≤ –0.002 *Km* ^–1^), and Neutral (–0.002 < $\frac{\partial \theta }{\partial z}$ ≤ 0.002 *Km* ^–1^).
Vertical shear magnitude (10-247m a.g.l.)	σ $\sigma \left|\frac{\partial \vec{v}}{\partial z}\right|$ ≤ 0.005 *s* ^–1^
Variance of the vertical turbulence (10-247m a.g.l.)	*σw* ^*'*2^ ≤ 0.15 *m* ^2^ *s* ^–2^
Synoptic wind (5km a.m.s.l.)	*σU _synop_* ≤ 5.16 *ms* ^–1^ *σV _synop_* ≤ 4.55 *ms* ^–1^
Transect-averaged wind (10-247m a.g.l.)	*σU*_*transect*_ ≤ 1.45 *ms* ^–1^ *σV _transect_* ≤ 0.52 *ms* ^–1^

To avoid the brute-force exhaustive computation, we implement a greedy “drop-until-threshold” algorithm. For each existing match of at least 5 wind profiles, we iteratively remove profiles that contribute most to exceeding the standard deviation threshold. Starting with the worst offending profile, we drop one at a time, recalculating the combined standard deviation after each removal, until the match satisfies the criteria or there are no wind profiles left to drop. While the algorithm ensures that the overall standard deviation is reduced below the threshold, individual values may still fall outside the specified limits.

**Figure 5.**
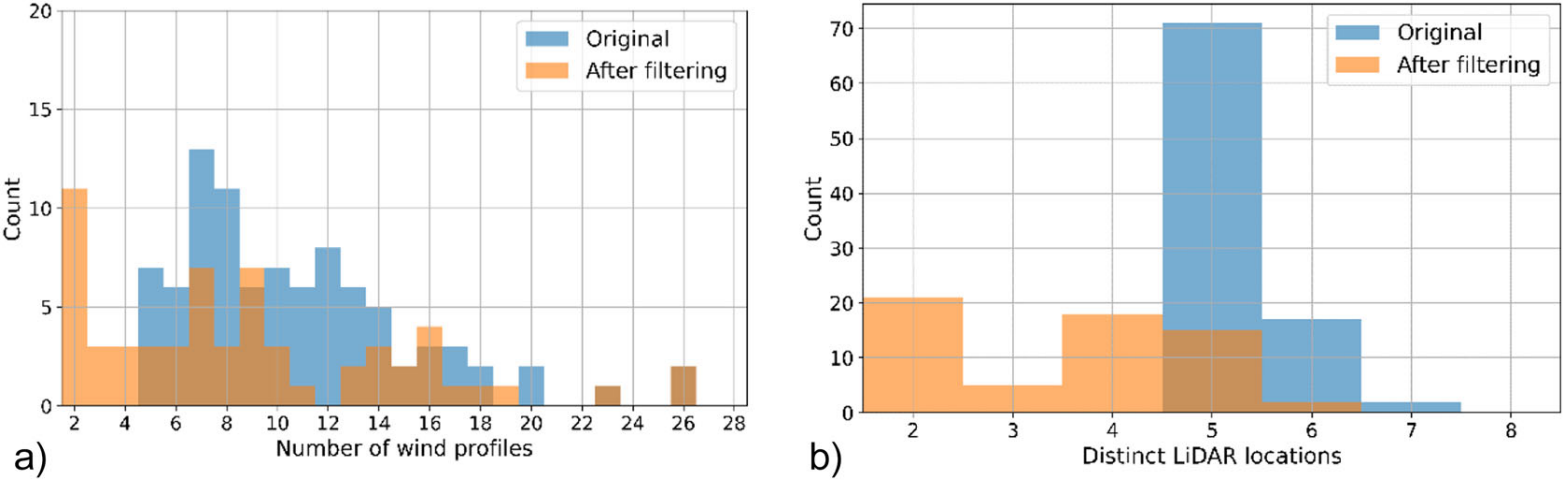
a) Match size and b) LiDAR locations before and after HICAR characteristic feature filtering.

Applying the stricter matching criteria to the nacelle-based matches reduces not only the total number of matches identified but also the composition of each match. For example, a nacelle-based match of 20 different wind profiles over 6 distinct LiDAR locations, that is filtered by the stricter HICAR criteria, could be reduced to only 10 profiles over 3 LiDAR locations. This filtering step is summarised in Figure 5, which shows the number of wind profiles, and distinct LiDAR locations for these matches before and after filtering. However, the resulting match can be considered significantly more robust as it not only has similar wind vectors at the five turbines, but also similar global radiation, atmospheric stability, shear, vertical turbulence, transect wind vectors and synoptic winds.

**Figure 6.**
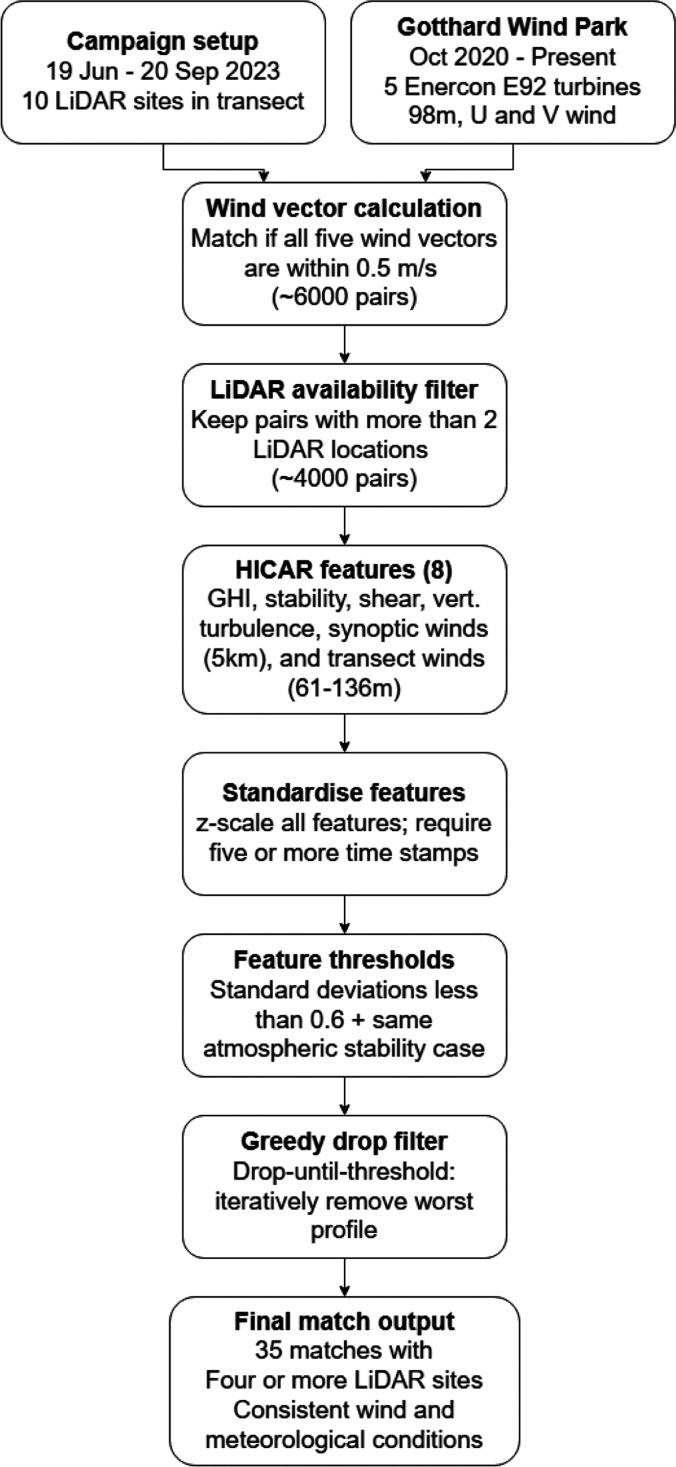
Flowchart of the matching algorithm methodology.

In total 35 unique matches with at least 4 different LiDAR locations are identified. These matches exhibit consistent wind and atmospheric conditions along the Gotthard Wind Park with LiDAR wind profile measurements along the pass. The 35 matches differ from another in general wind patterns and atmospheric conditions, which allows for a comprehensive analysis of the typical wind patterns that are observed on the Gotthard pass, which can help in explaining the turbine efficiency. The matching algorithm methodology is summarised in a flowchart shown in Figure 6.

## Results

Having established a robust matching algorithm for the sequential LiDAR measurements along the transect in the Gotthard wind park, the matched wind profiles can now be visualised along the transect, with all three wind components along the pass at any height of the measurement range. Previously, such visualisations were only possible using a triple wind-Doppler LiDAR setup with synchronised measurements.

### LiDAR horizontal flow homogeneity assumption

In complex terrain, concerns may arise regarding the applicability of Doppler wind LiDARs, as horizontal flow inhomogeneities can violate the homogeneity assumption underlying the retrieval of vertical wind profiles. The WindCube V2.1 profiler employs four beams inclined 28° from the vertical, corresponding to a horizontal separation of approximately 100 m between opposing beams at 100 m height. To evaluate potential impacts of horizontal inhomogeneity, we analysed flow field simulations across representative distances of 100 m (at 100 m height) and 300 m (at 300 m height) in all four cardinal directions. This shows that the mean biases in all wind components remain below 0.1 *ms* ^–1^, which is negligible relative to the intrinsic measurement uncertainty of the LiDAR. Standard deviations are generally below 0.5 *ms* ^–1^, with slightly larger values in the north–south direction, attributable to valley channelling, and in the east–west direction near the surface, likely related to surface effects. Although the flow field simulations do not represent thermally driven flows such as katabatic or anabatic winds, which may increase variability in reality, the observed deviations are on the order of the intrinsic error of the LiDAR, thus supporting the validity of the horizontal homogeneity assumption. Consequently, LiDAR-derived vertical profiles can be interpreted without additional corrections. Additionally, at the horizontal scales relevant for the LiDAR measurements, the HiCAR v1.1 model resolution (50 m) does not resolve sub-grid flow variability within the LiDAR sampling volume. Consequently, model-derived mean flow fields appear horizontally smooth over the transect length and are therefore not well suited to visually illustrate the LiDAR horizontal homogeneity assumption.

### Gotthard pass wind characteristics

**Figure 7.**
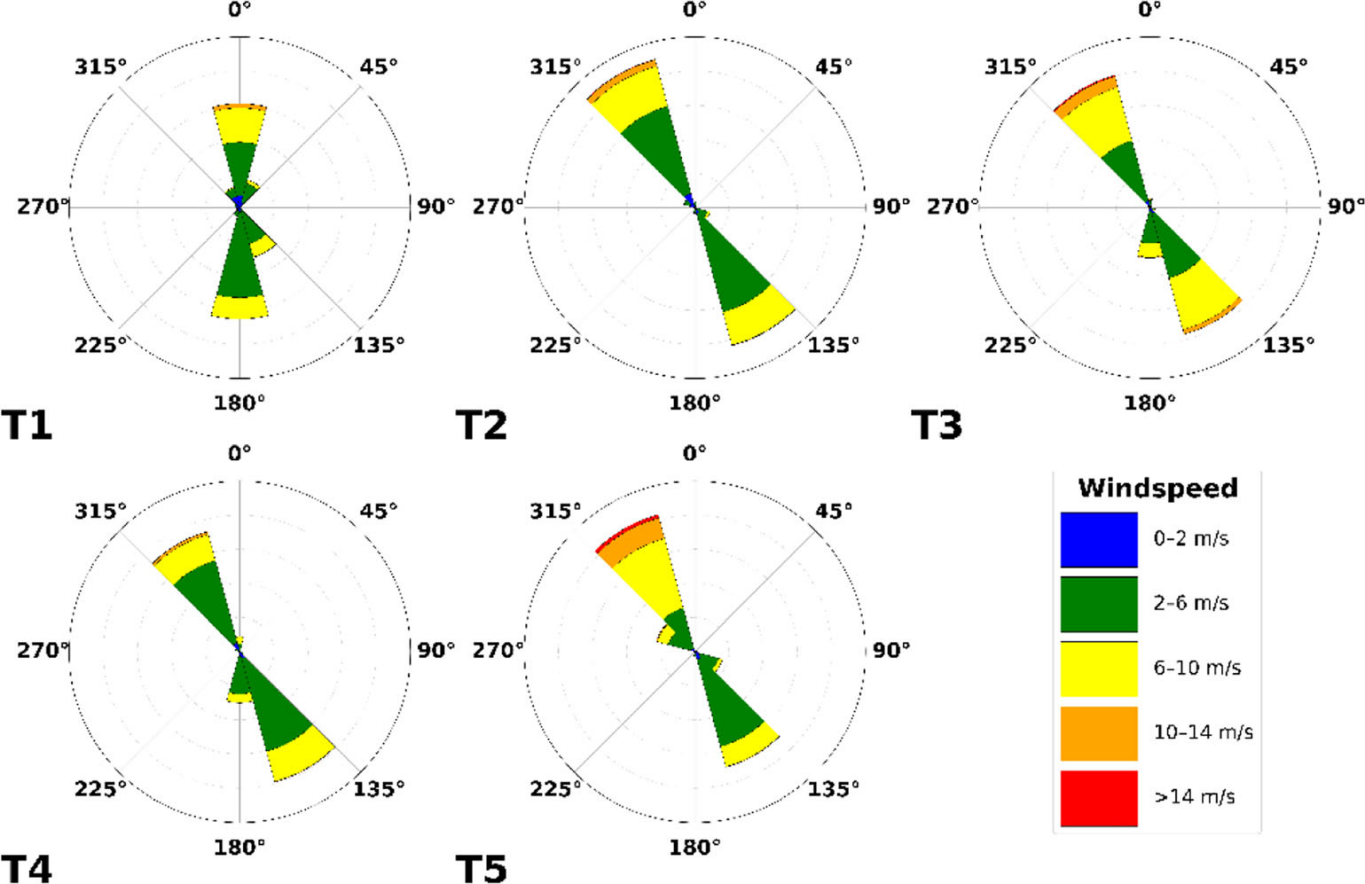
Wind roses of the five turbines in the Gotthard Wind Park based on all three years of data since its construction.

Firstly, the wind patterns observed on the pass at the height of the turbine nacelles are illustrated by wind roses in Figure 7. Here, northerly winds can be observed to be generally stronger than southerly winds. Particularly, wind speeds above the rated wind speed of the E92 turbine are much more prevalent. We define southerly winds as originating between the azimuths 75° and 255° and northerly wind from the remaining 180° sector. These distinct differences can be recognised in the Weibull distributions (Equation 5), which are split based on the two prevailing wind directions in Figure 7.

$$f(v)=\frac{k}{\lambda }{\left(\frac{v}{\lambda }\right)}^{k-1}\;\exp\;-\;{\left(v/\lambda \right)}^{k}$$ (5)

Weibull parameters scale shape *k* [-] and *λ* [m/s] parameters, and the measured mean wind speed $\left|\vec{v}\right|$ [m/s], split for northerly and southerly winds (75° < *θ* ≤ 255°) for the Gotthard wind turbines.

**Figure 8.**
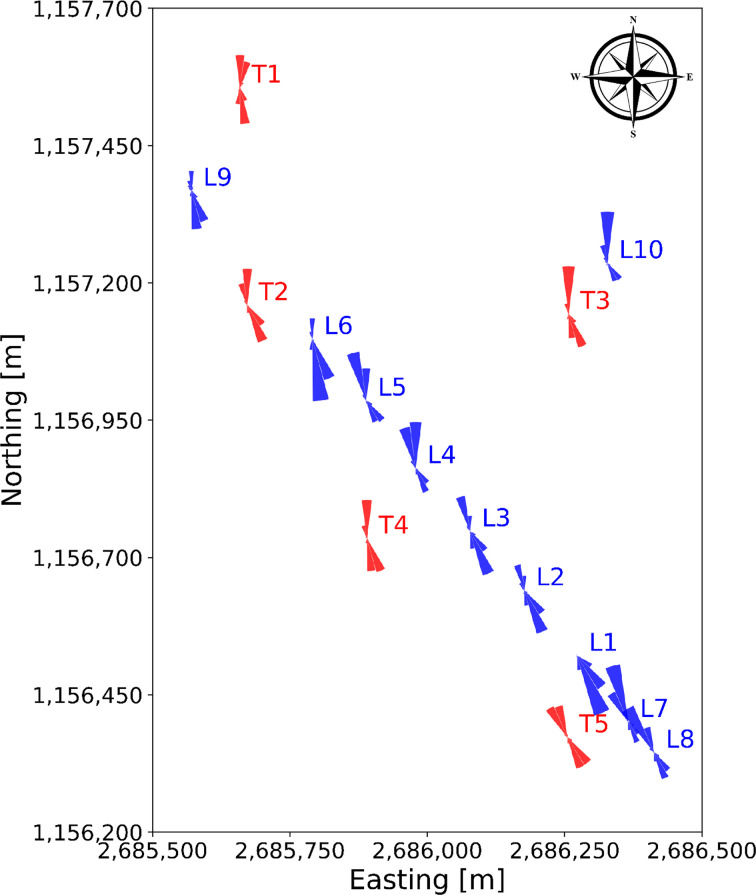
Spatial progression of wind direction over the Gotthard wind park, illustrated by wind roses at around 100 m above ground level.

In Figure 8, the wind roses at approximately 100 m above the ground of all the turbine and LiDAR locations are shown. The blue wind roses that are generated from LiDAR data do not exhibit the correct proportions in the frequency of the wind direction, as often only one week of data is available at a given location. However, the wind roses illustrate the spatial progression of the general wind direction along the Gotthard wind park well. Particularly, the differences for northerly and southerly winds at any given location on the pass can be observed.

### Matching algorithm statistics

**Figure 9.**
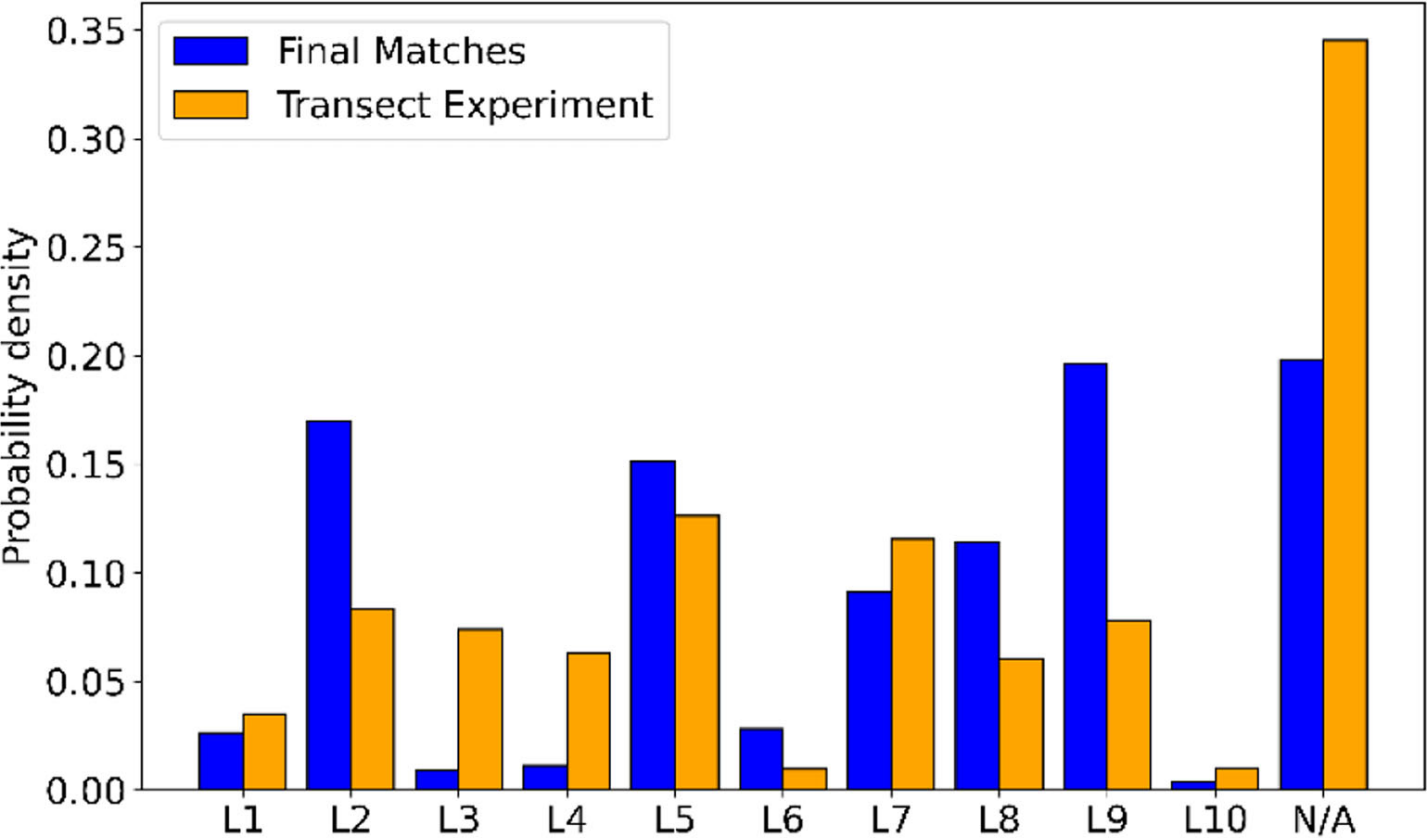
Frequency of LiDAR locations, matches vs. full dataset.

Figure 9 shows the distribution of LiDAR locations throughout the Gotthard Transect Experiment in orange. In the majority of the campaign period, there were no LiDAR wind profiles available (N/A column). The distribution across the 10 LiDAR sites varies considerably due to the weekly relocation of the instrument, which often included periods of missing data as the experiment was not tended to on a daily basis. As a result, some sites are more present in the final matched dataset than others. In particular, L2, L5 and L9 dominate the distribution because these sites happened to sample a large number of northerly winds. As discussed in more detail in the following subsection, the northerly winds are more likely to be matched as this wind regime seems to be more consistent in wind speed as well as the steadiness of the streamlines, exhibiting less shear and veer than the Southerly winds.

**Figure 10.**
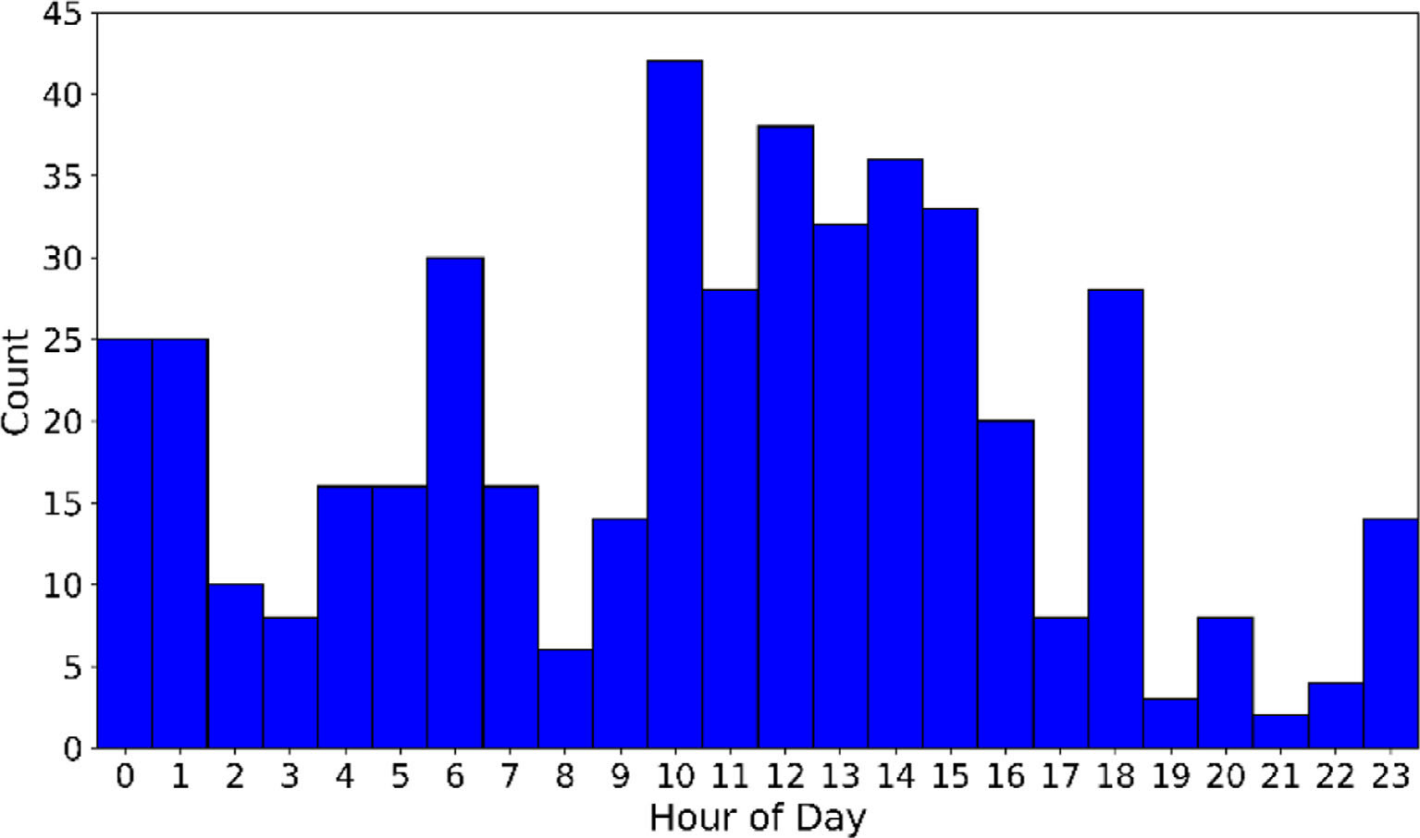
Distribution of time in final matches.

Figure 10 presents the distribution of matched timestamps as a function of the hour of day. The matches are not uniformly spread, like the original transect experiment dataset. Distinct diurnal peaks are observed where a majority of matches occurring during daytime hours, when solar heating promotes boundary-layer turbulence and characteristic vertical wind shear patterns which are repeating often. A second cluster can be observed during night hours (23-01 UTC), likely reflecting stable nocturnal conditions that are also well represented in the HICAR characteristic parameters. Finally, peaks occur near the end of the night (06 UTC) and near the end of the afternoon (18 UTC), capturing transient stability transitions. Although the hour of day itself was not an explicit matching variable, these results demonstrate that the matching algorithm does also distinguish different parts of the diurnal cycle, based on the forcing data in the HICAR simulations alone.

### Transect wind profile evolution

**Figure 11.**
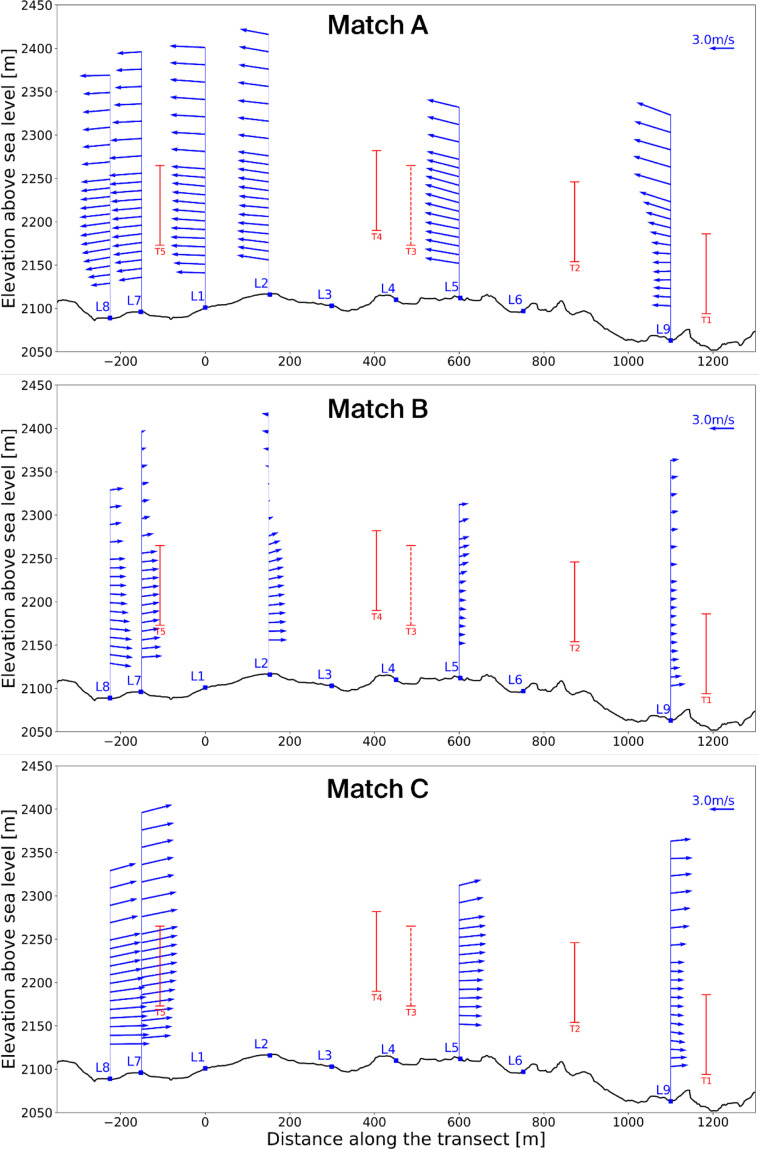
Side view of three characteristic matches on the Gotthard Pass. Turbines in red.

**Figure 12.**
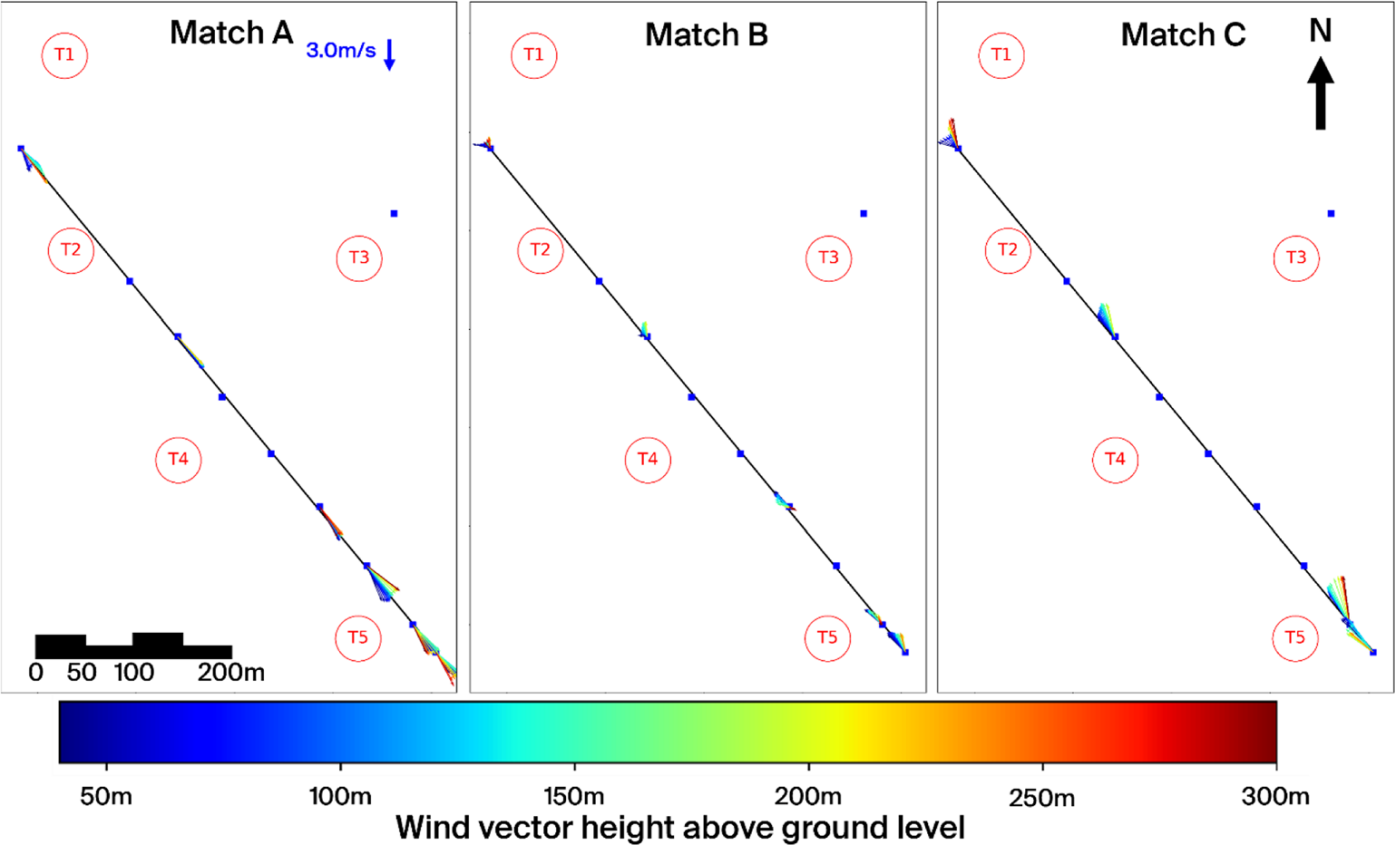
Top view of three characteristic matches on the Gotthard Pass. Turbines in red.

To illustrate the variability of flow conditions across the transect, we highlight three representative matched wind profiles (A–C). Of the 35 matches with at least four lidar profiles, 26 (74%) correspond to northerly winds, 5 (14%) to southerly winds, and 4 (12%) to low-wind cases where no dominant wind direction could be determined. Figure 11 and Figure 12 present these three selected matches in side and top view, respectively, while Table 4 summarises the associated timestamps and characteristic parameters from the matching algorithm.

**Table 3.** Weibull fit parameters of the Gotthard Wind Park for Northerly and Southerly winds.

Turbine	Northerly wind			Southerly wind		
	*k*	*λ*	$\left|\vec{\text{v}}\right|$	*k*	*λ*	$\left|\vec{\text{v}}\right|$
**1**	1.31	5.14	4.16	3.53	5.27	4.76
**2**	1.93	5.31	4.67	3.38	5.38	4.84
**3**	2.33	6.65	5.75	2.60	6.29	5.52
**4**	2.54	5.89	5.11	3.03	5.18	4.67
**5**	2.64	7.66	6.59	2.62	4.80	4.31

**Table 4.** LiDAR transect metadata of the matches displayed in Figure 11 and Figure 12.

LiDAR location	Match A	Match B	Match C
**L1**	18:40 19-06-23	-	-
**L2**	11:50 07-07-23	01:00 11-07-23	-
**L3**	-	-	-
**L4**	-	-	-
**L5**	14:30 31-07-23	06:10 30-07-23	05:30 31-07-23
**L6**	-	-	-
**L7**	11:50 24-08-23	01:20 24-08-23	23:00 19-08-23
**L8**	16:50 02-09-23	02:40 03-09-23	03:20 03-09-23
**L9**	13:50 10-09-23	02:20 11-09-23	06:00 11-09-23
**L10**	-	-	-
**Wind park wind direction**	Northerly	Southerly	Southerly
**Global Horizontal Irradiation [Wm** ^**−2**^ **]**	624	13	47
**Atmospheric stability [class]**	Unstable	Stable	Stable
**Vertical shear magnitude [s** ^**−1**^ **]**	0.0068	0.0070	0.0055
**Variance of vertical turbulence [m** ^**2**^ **s** ^**−2**^ **]**	0.092	0.048	0.029
**Synoptic wind speed [ms** ^**−1**^ **]**	8.0	10.2	7.7
**Synoptic wind direction [°]**	245	277	292
**Transect wind speed [ms** ^**−1**^ **]**	9.45	1.48	1.30

**Match A (northerly winds):** This case represents the dominant flow regime during the experiment: daytime northerly winds under unstable conditions, with relatively strong incoming solar radiation ( *GHI* ≈ 624 *Wm* ^–2^). The wind speed is relatively strong and shows little vertical shear due the strong vertical mixing as reflected by the HICAR simulations. At L9, the wake of Turbine 1 can be identified in the lower part of the profile, while further downstream the flow is slightly deflected upward before curving downward again near L7–L8. Only minor veering (a few degrees) is visible throughout the profile, which can be considered negligible. And near L8 a shearing effect can be consistently observed in the lower 100m of the wind profile, which can be attributed to the small north facing escarpment on which T5 is located. Overall, this northerly pattern is representative of the majority of final matches. The most consistent effect is a modest channelling that accelerates the flow towards the southern turbines (T3 and T5), which in turn produces relatively more power compared to the northern turbines (T1, T2, T4). Because the structure of northerly flows is fairly uniform across the matches, we only show one representative example here.

**Matches B and C (southerly winds).** In contrast, the two southerly cases were both matched under weak solar input ( *GHI* ≈ 50 *Wm* ^–2^), occurring around 06 UTC or during nocturnal hours. Given summer daylight savings in Switzerland (UTC+2), these periods still correspond to pre-sunrise conditions. Both cases are characterised by stable atmospheric stratification, which enhances vertical. In Match B, a particularly interesting feature appears: a reversal in flow direction around L2, with signs of shear already emerging upstream at L7–L8. The cause of this localized reversal is not entirely clear but may be linked to terrain-induced flow separation or local recirculation in stable conditions. This flow reversal is observed regularly in other wind profiles with slow southerlies. The wind also decelerates near the end of the transect. Match C shows a similar downstream deceleration but without reversal; instead, strong localised shear is evident near Turbine 5 (and, in other cases, also at Turbine 3), which likely impacts turbine efficiency. These two cases highlight the more complex flow behaviour under southerly winds, where we examine these shear effects and their implications for turbine performance in more detail.

### Wind shear and veer

For northerly winds, shear is consistently observed near L8 at the southern end of the transect, largely caused by the small hill where Turbine 5 is located. Under southerly winds, however, the more dominant influence is the Scara Orello mountain (2242 m) in the Tremola Valley ( Figure 12). This prominence rises about 140 m above the wind park and creates a clear separation between air layers. Higher-altitude winds pass over the obstacle and continue northwest along the Tremola Valley, while the lower flow is diverted around the mountain and redirected northward over the pass.

Under idealized conditions in the planetary boundary layer, wind direction is expected to veer with height in the Northern Hemisphere as a result of the Ekman balance between surface friction and the Coriolis force. This Ekman-induced veering typically develops gradually over several hundred meters in the vertical. At L8, however, the observed wind direction changes with height strongly deviate from this classical framework and are instead dominated by local topographic forcing, which overwhelms and distorts the background Ekman veering.

This terrain-induced channelling produces a distinct horizontal veering with height that far exceeds what could be explained by the Coriolis force. Over the 1.5 km length of the Gotthard upper plateau, the maximum deflection by Coriolis would be only about 0.5°, whereas the observed turning is an order of magnitude larger. The separation of air layers around turbine hub height has direct implications for wind turbine performance: Turbines 4 and 5, which interact more with the deflected lower layer, are more strongly affected by veering and shear, while Turbine 3, which is located somewhat higher on the plateau but in the line of topographic prominence, is exposed to strong turbulent flow.

### Turbulence kinetic energy

Turbulence kinetic energy (TKE) *k* [ *m* ^2^ *s* ^–2^], described by Equation 6 where *σ _v_* [ *ms* ^–1^] is the standard deviation of the wind component, exhibits bi-modal distributions where northerly and southerly winds exhibit very different TKE characteristics. This bi-model distribution is similar to the wind speed along the LiDAR transect as shown in Figure 14. In these figures, the red lines represent the probability density functions of the TKE, while the blue vertical lines indicate the mean TKE, allowing for a visual identification of where turbulence is strongest along the transect.

$$k=\frac{1}{2}(\sigma_{U}^{2}+\sigma_{V}^{2}+\sigma_{W}^{2})$$ (6)

**Figure 13.**
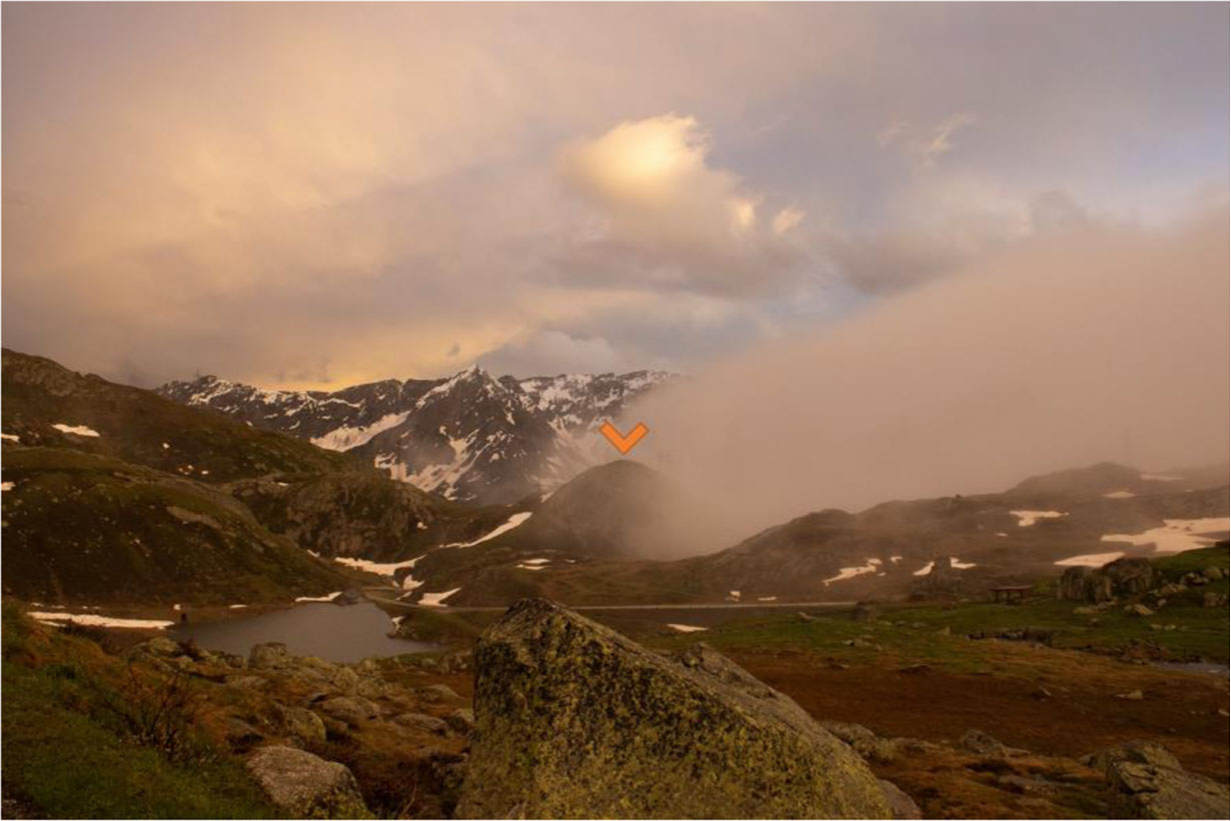
Indication by the cloud layer of the horizontal stratification for southerly winds due to the Scara Orello mountain (orange).

**Figure 14.**
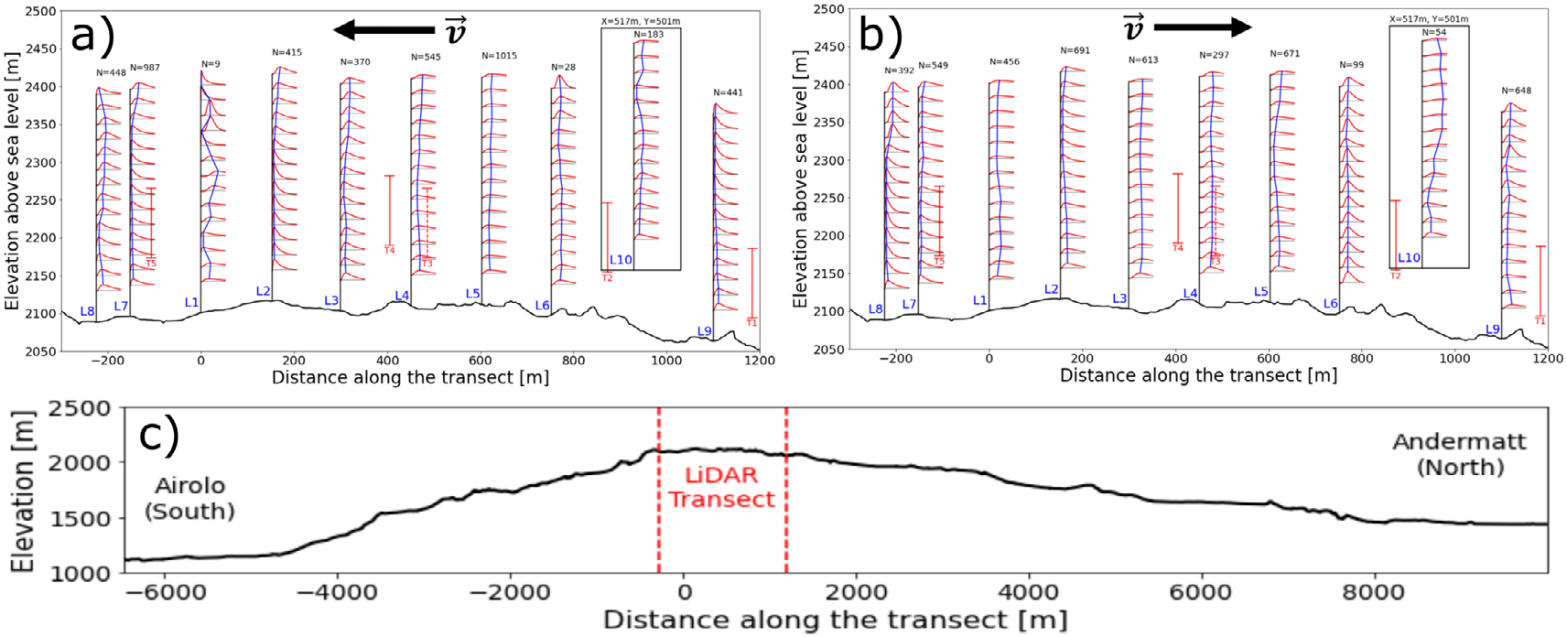
Log-normal fitted TKE PDFs along the Gotthard pass: a) Northerly winds, b) Southerly winds, c) extended elevation profile.

For both northerly and southerly winds, the air enters the wind park at low turbulence levels. As the air moves further over the pass, the turbulence kinetic energy gradually increases. This is to be expected as the wind tends to accelerate on the upper plateau due to the increased density of streamlines as the air is pushed over the highest point of the pass and the overall kinetic energy of the wind increases, this will also contribute partly to the increased turbulence kinetic energy. Additionally, the surface obstacles, such as boulders, buildings and wind turbines will partly contribute to the increase of turbulence in the flow. For southerly winds Figure 14b, the increased turbulence in the lee of Turbine 5 can be observed in L1, L2 and L3 around the swept area of the turbine. This effect is not observed for northerly winds, which can be explained by the fact that these LiDAR locations are not directly in the lee of T2 and T4. This is confirmed by the lack of wake effects, typically showing a substantial decrease in wind speed at the elevation of the turbine rotor, observed in these LiDAR profiles.

At measurement L10 the greatest turbulence kinetic energy can be observed for southerly winds. This correlates with the increased southerly wind speeds which were observed during the measurement period at this site. Additionally, the upwind terrain on this side of the pass is notably more complex, with the aforementioned Scara Orello mountain (2242m) obstructing the wind for the western side of the pass. This will likely affect the flow on the eastern pass in a similar way.

We illustrate for the case of a symmetric deviation from a mean wind speed with standard deviation how the effect of non-linearity results in additional power that can be harvested. If we further assume that such a deviation gives the approximate magnitude of the power gain by turbulence, we can estimate the effective energy the turbulence holds for the type E-92 turbine on the Gotthard plateau. Consider the wind turbine power *P*( *v*) [ *W*] described by Equation 7:

$$P(v)=\frac{1}{2}\rho Av^{3}$$ (7)

*ρ* = 0.996 ± 0.021 *kgm* ^–3^ is the air density on the Gotthardpass, *A* = *πr* ^2^ = 6650 *m* ^2^ the swept area of a wind turbine, *v*[ *m*/ *s*] the incident wind speed. Since the horizontal standard deviation of the wind for the WindCube is defined by $\sqrt{\sigma_{u}^{2}+\sigma_{v}^{2}}=\sigma_{horz}[ms^{-1}],$ we can consider a deviation *σ _horz_* above and below the regular wind speed *v.* Equation 8 provides a first-order estimate of how turbulence around the mean affects turbine power output and can reach up to around 80kW of deviation from the typical wind power curve:

$$P_{turbulence}=\frac{P(v+\sigma_{horz})+P(v-\sigma_{horz})}{2}-P(v)=\frac{3}{2}\rho \;A\;v\;\sigma_{horz}^{2}>0$$ (8)

The actual effect of turbulence is governed by the curvature of the turbine power curve: In convex sections of the power curve, positive and negative fluctuations are weighted asymmetrically, leading to a net increase in power relative to the mean wind speed. Whereas in concave sections, the reverse occurs and fluctuations produce a net decrease in power. Therefore Equation 8 represents only a rough upper-bound to the influence of turbulence on power production. It does not account for the changing curvature of the real power curve, nor for higher-order asymmetries in the wind speed distribution. For a full quantification, we refer the reader to a work that integrates the measured wind speed probability density function and other vertical variations with the actual turbine power curve as referred to in international standards. ^47^

Furthermore, we have validated the assumption of second-order symmetry in the wind speed variations measured the Doppler wind LiDAR. The 1-second wind speed data from the WindCube during the campaign shows that the deviations from the mean are not perfectly normally distributed. They rather remain symmetric up to second order and can be fitted optimally with a Student-t distribution ( *ν* = 2.88 ± 0.02, *R* ^2^ = 0.996), which captures the heavy tails observed. From this the mean horizontal deviation can be expressed as $\sigma_{horz}=\sqrt{v/(v-2)}=1.81,$ Which seems to be independent of height. Which consequently validates that the rough upper-bound still holds.

### Regional weather correlations

Subsequently, based on the matching timestamps, the IMIS and SLF stations were assessed in an attempt to find correlations and patterns between the wind on the Gotthard Pass and other atmospheric variables in the vicinity of the pass. These correlations can be of interest in generalising the local high-resolution measurements to nearby locations, enabling an extended domain of the wind energy assessment. Additionally, potential correlations can help in future case studies to characterise specific weather systems from weather models to predict wind profiles around the study area itself.

Regarding the mountainside- and mountaintop-located IMIS stations, which record data at a 1 hour resolution, no strong correlations (| *r*| < 0.4) can be found in any of the atmospheric variables relative to any data collected during the Gotthard 2023 campaign. The mountaintop stations provide moderately better correlations as they better represent the synoptic weather conditions and are less prone to small-scale local topographical effects. A simple model to predict northerly or southerly winds in the Gotthard wind park remains too challenging for simple linear correlation models, however. The mountainside stations generally had worse correlations (| *r*|< 0.3). This is attributed largely to the different slope aspects of the sites where the stations are located, far away from the Gotthard wind park itself.

Finally, the MeteoSwiss weather station Gütsch installed 11 km NNE of the Gotthard wind park, with an elevation of 180 m above the Gotthard wind park on an east-west ridgeline provides the best data for this analysis. Although the wind components measured at Gütsch still lack correlation with those in the Gotthard wind park, barometric pressure, air temperature and humidity strongly correlate with the variables that were measured at Gotthard Pass during the 2023 campaign. Particularly, temperature and pressure can be used to approximate their equivalence on Gotthard when our instruments are not present or had data gaps by using a lapse rate of 6.5°C per km and the barometric equation (+17.42 ± 0.36 *hPa* on Gotthard), respectively. The relative humidity is less correlated to that measured on Gotthard, however, since it is only used for estimating air density on the Gotthard Pass, which is weakly sensitive to relative humidity, we assume the same humidity for both locations. This estimated air density is used to estimate the general turbine efficiency decrease, which can not be attributed to complex terrain interactions in the following section.

### Wind turbine efficiency

**Figure 15.**
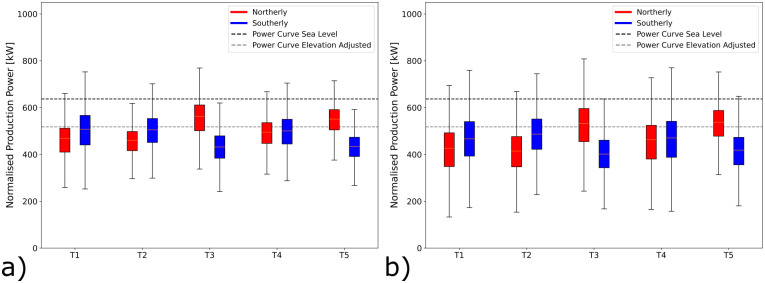
Power generated by turbine and wind directions. ( **a**) summer campaign period (19 June 2023 – 20 September 2023), ( **b**) the entire dataset (October 2020 – September 2023).

The bi-directional wind system across the Gotthard is prevalent for the wind shear, wind veer and turbulence on the pass. The compound effects of these combine, and occasionally cancel each other when the air parcels transfer their energy to the wind turbine blades. To study the impact of terrain and flow effects on the efficiency of the five wind turbines, the production power distributions for northerly and southerly winds are shown in Figure 15. Wind speeds are selected between the cut-in speed and the rated wind speed, where the relation *P* ∝ *v ^3^* holds. In the range of 4 *ms* ^–1^ ≤ *v* ≤ 10 *ms* ^–1^, small disturbances and perturbations of the flow have the largest impact on power production. The power production is normalised to 7 *ms* ^–1^ by the cubic relation between power and wind speed in this range with Equation 9.

In Figure 15a where only the power data during the measurement campaign is shown, consider the northern Turbines 1 and 2. The southerly winds are more efficient at transferring energy to the wind turbine than their northerly counterparts. This discrepancy can be explained partly by the increased turbulence intensity as the wind moves over the upper plateau before it reaches these turbines. For the northerly winds there is still a significant vertical wind speed component incident on the turbines increasing blade stresses and, making the programmed angle of attack of the turbine blades less efficient.

$$P_{normalised}=P(v)\frac{C_{p}(7ms^{-1}){\left(7ms^{-1}\right)}^{3}}{C_{p}(v)v^{3}},4\;ms^{-1}\leq v\leq 10\;ms^{-1}$$ (9)

Remaining in Figure 15a, the central Turbine 4 generally experiences the benefits of the northerly and southerly winds equally with very low turbulence for both directions as well as moderate wind shear and veer only slightly reducing the turbine efficiency. Vertical wind components play almost no role for this turbine.

The most interesting sites are Turbines 5 and 3 during the campaign period, shown in Figure 15a. These turbines not only benefit from flow acceleration for northerly winds, which generally bring higher wind speeds to this part of the pass. Remarkably, they can also transfer this energy more efficiently than expected when referring to the manufacturer’s power curve when corrected for the reduced air density on the Gotthard Pass as a result of the elevation. This apparent out-performance can be explained by the increased turbulence which reduces the average wind speeds to lower and unrealistic values due to the limitations of the nacelle-mounted anemometer, whilst the kinetic energy of the wind is higher than indicated. These effects are exaggerated most for the strong northerly winds which accelerate to much greater wind speeds before reaching these turbines due to the linear scaling with the average wind speed *v*, as shown by Equation 8. The wind shear and wind veer for these northerly winds are already reduced to a minimum as observed for southerly winds for Turbines 1 and 2, and thus do not decrease the performance. Meanwhile, the strong shear and veer for southerly winds, as observed in Match B and C, which has a detrimental effect on the power production. We do not observe the same apparent increase in power output due to turbulence for the northern Turbines 1 and 2, as the wind speeds and turbulence are generally lower.

During snow-covered periods, which are not included in our LiDAR measurement period, but are partly represented in the longer time domain of Figure 15b, a general decrease in efficiency is observed over all the turbines. In addition to surface-related and atmospheric effects, blade icing during snow-covered periods may further reduce aerodynamic efficiency and contribute to the observed performance decrease. While icing is not explicitly quantified in this study, it is a known factor affecting turbine performance in high-alpine winter conditions. This can likely be explained by the multiple meters thick snow layer covering the mountain pass for 7 months per year smoothing out the terrain, reducing the apparent power increase by turbulence. The effect of the smoother surface which increases wind speeds is filtered out by the power normalisation in Equation 9. The high performance of Turbines 3 and 5 is therefore reduced the greatest amount for northerlies, as they experienced the greatest discrepancies in power production in summer time. There may be additional factors such as the presence of a stable atmospheric boundary layer due to the greatly reduced turbulent heat flux over the snow, resulting in more vertically stratified wind profiles that show more shear and veer and less turbulent airflow incident on the turbines. We refer the reader to continuation work on this topic. ^49^

### Sequential LiDAR vs synchronised LiDAR

The sequential and synchronised LiDAR methods are both designed to reconstruct three-dimensional wind fields, but they differ considerably in logistics, costs, and data quality. The sequential method requires only a single LiDAR, making equipment costs relatively low. However, this approach demands regular relocation of the instrument, which increases personnel time and logistical complexity. By contrast, synchronised methods use two or more LiDARs operating in parallel. These require extensive pre-campaign planning, advanced expertise in operating multiple instruments, and more involved post-processing to merge datasets into a consistent wind field.

The sequential approach is therefore simpler to deploy and requires less upfront planning, though it relies more heavily on numerical modelling (e.g. flow field simulations) to reconstruct missing flow structures. Such expertise is increasingly common in the commercial wind energy sector, making sequential methods more accessible. Synchronised LiDARs, on the other hand, can achieve higher spatial resolution and cover larger domains by combining measurements from multiple instruments. Their performance is still constrained by the laser stare-time needed to collect enough return photons, but modern systems can capture up to 300 unique points in a 10-min averaging period, the standard in wind energy assessment. For the 20 range gates used in this study, a three-LiDAR synchronised system could effectively reproduce a transect equivalent to 15 sequential profiles, while providing much higher data availability since no matching algorithm is required. ^50^

## Conclusion

This work presents a novel method for high-resolution three-dimensional wind profile measurements in channelized flow conditions within complex terrain using a sequential spatially distributed LiDAR deployment. The project took place in the Gotthard wind park during the summer of 2023. The sequential LiDAR deployment is made along a transect on the western side of the upper plateau of the Gotthard Pass at an elevation of around 2100 m a.s.l., using a profiling VAISALA WindCube V2.1 wind-Doppler LiDAR instrument. The transect of nine sequential LiDAR locations and an additional tenth deployment on the eastern side of the pass aims to probe the entire wind park closely following the sites of five Enercon E92 wind turbines, constituting the high alpine Gotthard wind park. The Gotthard Pass is located in the centre of the Alps and features many topographical effects and flow features typical of complex terrain that influence the wind profile that is incident on the wind turbines and which also can affect the performance of the latter. It is one of the few alpine passes that experiences both northerly and southerly Föhn winds, and wind channelling and acceleration over the pass can be seen from the measured sequential LiDAR wind profiles.

A matching algorithm is proposed to allow for inter-comparison of LiDAR wind profiles measured at different moments in time, but for similar incoming wind conditions, verified by the meteorological observations on the nacelles of the five turbines and typical atmospheric parameters derived from simulations of the atmosphere. Using eight characteristic features from the HICAR v1.1 model and applying strict standard deviation thresholds, we substantially refine the nacelle-based matching approach. This filtering reduces the total number and size of matches but ensures that the retained combinations represent wind profiles that are coherent not only in terms of turbine nacelle wind speed, but also in radiation, stability, shear, turbulence, and both transect-scale and synoptic flow. Although matches are fewer after filtering, their robustness is greatly enhanced, providing higher confidence that the selected profiles capture consistent inflow and atmospheric conditions across the Gotthard wind park. This matching algorithm is successful in identifying a substantial number of similar wind profiles throughout the three-month field campaign, with a maximum of six wind profiles measured at different locations, which are capable of describing the typical winds observed on the Gotthard pass.

The sequential LiDAR data provides a unique visualisation of wind shear and wind veer along the alpine pass, as well as turbulence profiles clearly illustrating the complex terrain influences that impact wind turbine performance in mountainous applications (Sections 3, 5). We provide a qualitative overview of the complex terrain effects that significantly influence wind turbine performance, both in terms of potential drawbacks and benefits. Particularly, the stronger northerly winds exhibit acceleration over the pass, increasing wind energy production greatly for turbines furthest downwind. For upwind turbines, wind veer and shear decrease turbine performance, but this effect is reduced further downwind, where the channelling of the narrow pass plateau reduces these effects. The turbulence is largest on the eastern side of the pass, independent of the wind direction, which can be partly explained by the local terrain being rougher compared to the western part of the plateau. The presence of turbulence causes an apparent increase in wind turbine efficiency, which can actually be attributed to kinetic energy in the wind being transferred to turbulence instead of the average wind flow. The nacelle mounted anemometer only measures the average wind speed, but misses out on the short-term wind speed deviations which have a non-linear positive effect on the energy production of the turbines. A qualitative assessment of the compound effects of complex terrain on the incident wind agrees well with the turbine data. The applicability of the presented approach may be reduced at exposed hilltop or ridge locations, where strong flow inclination and separation can violate the homogeneity assumptions required for ground-based Doppler LiDAR measurements.

We hypothesise that a terrain surface smooth by snow during the 7 month long snow-covered winter period reduces turbulence, which is supported by observations from nacelle-mounted anemometers. In future work, we plan to explore in more detail the effect of the presence of a smooth snow pack, which covers the wind park for 7 months of the year. Secondly, more complex interactions between air density and atmospheric stability outside of the summer period, and how this influences wind turbine performance will be tested. The current measurements in the region lack consistent and reliable atmospheric variables, requiring improved sensor coverage for this purpose. The sequential LiDAR measurement technique is an excellent candidate to improve wind profile characterization in channelized wind sites within complex terrain, where flow alignment and limited separation support the underlying LiDAR measurement assumptions.

### Ethics and consent

Ethical approval and consent were not required.

## Data Availability

All data collected during the Gotthard experimental campaign are publicly available via Zenodo: Sequential Wind-Doppler LiDAR wind profile measurements on the Gotthard pass in Switzerland – Summer 2023, DOI: 10.5281/zenodo.14524723. ^52^ This project contains the following underlying data: Wind-Doppler LiDAR measurements along the transect in the Gotthard Wind Park. This project contains the following underlying data:
•
Gotthard_Transect_Experiment_STA_standard_Final.nc •
Opening_netCDF_4_formats.docx Data are available under the terms of theCreative Commons Attribution 4.0 International. The authors have extracted weather data from MeteoSwiss and WSL SLF IMIS weather stations which are available upon request to the respective institutions. The wind turbine data made available by Azienda Elettrica Ticinise ^51^ was shared with the authors under non-disclosure agreement for this research project. One dataset that was used in our study has time-series data of wind power, wind speed and control algorithms of the wind turbines. This data was disclosed to the laboratory only under NDA. Readers, with interest for this data, can make a special request to the corresponding author after which the company will consider whether they will share this data under similar NDA conditions. The HICAR v1.1 simulations made to complement the measurements is too large to share and are made available upon request to the corresponding author.

## References

[1] Elgendi M AlMallahi M Abdelkhalig A : A review of wind turbines in complex terrain. *Int J Thermofluids.* 2023;17:100289. 10.1016/j.ijft.2023.100289

[2] Hyvärinen A Segalini A : Effects from complex terrain on wind-turbine performance. *J Energy Resour Technol.* 2017;139(5):051205. 10.1115/1.4036048

[3] Swiss Federal Office for Energy : Energy strategy 2050 once the new energy act is in force.2018. Reference Source

[4] Meyer L Koller S Froidevaux P : Windpotenzial Schweiz2022: Schlussberich zum Windpotenzial Schweiz 2022. Bern,August, 2022. Reference Source

[5] Qosja S Rolle R Gebremedhin A : Solving the bottleneck issue of energy supply. Case study of a wind power plant. *Int J Web Eng Technol.* 2022;5(2):874–891. 10.15157/IJITIS.2022.5.2.874-891

[6] Spiess H Lobsiger-Kägi E Carabias-Hütter V : Future acceptance of wind energy production: exploring future local acceptance of wind energy production in a Swiss Alpine Region. *Technol Forecast Soc Change.* 2015;101:263–274. 10.1016/j.techfore.2015.06.042

[7] Vignali S Lörcher F Hegglin D : A predictive flight-altitude model for avoiding future conflicts between an emblematic raptor and wind energy development in the Swiss Alps. *R Soc Open Sci.* 2022;9(2):211041. 10.1098/rsos.211041 35154790 PMC8826134

[8] Apostol D Palmer J Pasqualetti M : The renewable energy landscape: preserving scenic values in our sustainable future. Routledge;2016. 10.4324/9781315618463

[9] Bradley S Strehz A Emeis S : Remote sensing winds in complex terrain - a review. *Meteorol Z.* 2015;24(6):547–555. 10.1127/metz/2015/0640

[10] Bechmann A Sørensen NN Berg J : The bolund experiment, part II: blind comparison of microscale flow models. *Boundary-Layer Meteorol.* 2011;141(2):245–271. 10.1007/s10546-011-9637-x

[11] Berg J Mann J Bechmann A : The bolund experiment, part I: flow over a steep, three-dimensional hill. *Boundary-Layer Meteorol.* 2011;141(2):219–243. 10.1007/s10546-011-9636-y

[12] Vassberg JC Tinoco EN Mani M : Abridged summary of the third AIAA computational fluid dynamics drag prediction workshop. *J Aircr.* 2008;45(3):781–798. 10.2514/1.30572

[13] Menke R Vasiljević N Mann J : Characterization of flow recirculation zones at the Perdigão site using multi-lidar measurements. *Atmos Chem Phys.* 2019;19(4):2713–2723. 10.5194/acp-19-2713-2019

[14] Wildmann N Kigle S Gerz T : Coplanar lidar measurement of a single wind energy converter wake in distinct atmospheric stability regimes at the Perdigão 2017 experiment. *J Phys Conf Ser.* 2018;1037(5):052006. 10.1088/1742-6596/1037/5/052006

[15] Venkatraman K Hågbo TO- Buckingham S : Effect of different source terms and inflow direction in atmospheric boundary modeling over the complex terrain site of Perdigão. *Wind Energy Sci.* 2023;8(1):85–108. 10.5194/wes-8-85-2023

[16] Wagner J Gerz T Wildmann N : Long-term simulation of the boundary layer flow over the double-ridge site during the Perdigão 2017 field campaign. *Atmos Chem Phys.* 2019;19(2):1129–1146. 10.5194/acp-19-1129-2019

[17] Barthelmie RJ Pryor SC Wildmann N : Wind turbine wake characterization in complex terrain via integrated Doppler lidar data from the Perdigão experiment. *J Phys Conf Ser.* 2018;1037(5):052022. 10.1088/1742-6596/1037/5/052022

[18] Barthelmie RJ Pryor SC : Impact of local meteorology on wake characteristics at Perdigão. *J Phys Conf Ser.* 2019;1256(1):012007. 10.1088/1742-6596/1256/1/012007

[19] Volkert H Gutermann T : Inter-domain cooperation for mesoscale atmospheric laboratories: the mesoscale Alpine programme as a rich study case. *Quart J Royal Meteorol Soc.* 2007;133(625):949–967. 10.1002/qj.95

[20] Flamant C Richard E Schär C : The wake south of the Alps: dynamics and structure of the lee-side flox and secondary potential vorticity banners. *Quart J Royal Meteorol Soc.* 2004;130(599):1275–1303. 10.1256/qj.03.17

[21] Schär C Sprenger M Lüthi D : Structure and dynamics of an Alpine potential-vorticity banner. *Quart J Royal Meteorol Soc.* 2003;129(588):825–855. 10.1256/qj.02.47

[22] Walser A Schär C : Convection-resolving precipitation forecasting and its predictability in Alpine river catchments. *J Hydrol.* 2004;288(1–2):57–73. 10.1016/j.jhydrol.2003.11.035

[23] Walser A Lüthi D Schär C : Predictability of precipitation in a cloud-resolving model. *Mon Weather Rev.* 2004;132(2):560–577. 10.1175/1520-0493(2004)132<0560:POPIAC>2.0.CO;2

[24] Mackenzie H Dyson J : Short term forecasting of wind power plant generation for system stability and provision of ancillary services. *Wind Integration Forum Proceedings.* 2017:16. Reference Source .

[25] Risan A Lund JA Chang CY : Wind in complex terrain—lidar measurements for evaluation of CFD simulations. *Remote Sens.* 2018;10(1):59. 10.3390/rs10010059

[26] Bingöl F Mann J Foussekis D : Conically scanning lidar error in complex terrain. *Meteorologische Zeitschrift.* 2009;18(2):189–195. 10.1127/0941-2948/2009/0368

[27] Wagner R, Bejdic J : WINDCUBE+ FCR test at Hrgud, Bosnia and Herzegovina.2014. Reference Source

[28] Kristianti F Dujardin J Gerber F : Combining weather station data and short-term LiDAR deployment to estimate wind energy potential with machine learning: a case study from the swiss alps. *Boundary-Layer Meteorol.* 2023;188(1):185–208. 10.1007/s10546-023-00808-y

[29] Dujardin J Lehning M : Wind-topo: downscaling near-surface wind fields to high-resolution topography in highly complex terrain with deep learning. *Q J R Meteorol Soc.* 2022;148(744):1368–1388. 10.1002/qj.4265

[30] Melani PF Di Pietro F Motta M : A critical analysis of the uncertainty in the production estimation of wind parks in complex terrains. *Renew Sustain Energy Rev.* 2023;181:113339. 10.1016/j.rser.2023.113339

[31] Baker WE Atlas R Cardinali C : Lidar-measured wind profiles: the missing link in the global observing system. *Bull Am Meteorol Soc.* 2014;95(4):543–564. 10.1175/BAMS-D-12-00164.1

[32] Panofsky M Zhao HA : Characteristics of wind profiles over complex terrain.In: *Wind Engineering 1983 3C: Proceedings of the Sixth international Conference on Wind Engineering.* Elsevier;1983;15(1–3):177–179.

[33] Clifton A Clive P Gottschall J : IEA wind task 32: wind lidar identifying and mitigating barriers to the adoption of wind lidar. *Remote Sens.* 2018;10(3):406. 10.3390/rs10030406

[34] Clifton A Barber S Stökl A : Research challenges and needs for the deployment of wind energy in hilly and mountainous regions. *Wind Energ Sci.* 2022;7(6):2231–2254. 10.5194/wes-7-2231-2022

[35] Fuertes FC Iungo GV Porté-Agel F : 3D turbulence measurements using three synchronous wind lidars: validation against sonic anemometry. *J Atmos Ocean Technol.* 2014;31(7):1549–1556. 10.1175/JTECH-D-13-00206.1

[36] Boquet M Thobois L : Wind resource assessment campaign with lidars and met mast in large and complex sites.2012;14. Reference Source

[37] Lang S McKeogh E : LIDAR and SODAR measurements of wind speed and direction in upland terrain for wind energy purposes. *Remote Sens.* 2011;3(9):1871–1901. 10.3390/rs3091871

[38] Barthelmie RJ Folkerts L Ormel FT : Offshore wind turbine wakes measured by sodar. *J Atmos Ocean Technol.* 2003;20(4):466–477. 10.1175/1520-0426(2003)20<466:OWTWMB>2.0.CO;2

[39] Crescenti GH Gutmann E Kruyt B : A look back on two decades of doppler sodar comparison studies. *Bull Am Meteorol Soc.* 1997;78(4):651–673. 10.1175/1520-0477(1997)078<0651:ALBOTD>2.0.CO;2

[40] Reynolds D : The High-resolution Intermediate Complexity Atmospheric Research (HICAR v1.1) model enables fast dynamic downscaling to the hectometer scale. *Geosci Model Dev.* 2023;16(17):5049–5068. 10.5194/gmd-16-5049-2023

[41] SLF : Description of automated stations. Accessed: Aug. 21,2025. Reference Source

[42] MeteoSwiss : Measurement values and measuring networks. Accessed:Aug. 21, 2025. Reference Source

[43] Kröpfli D Schlegel T Geissmann M : Jahresmittel der modellierten windgeschwindigkeit und windrichtung.Bern,October, 2022. Reference Source .

[44] Sherman CA : A mass-consistent model for wind fields over complex Terrain. *J. Appl. Meteorol. Climatol.* 1978;17(3):312–319. 10.1175/1520-0450(1978)017>0312:AMCMFW>2.0.CO;2

[45] Reynolds D Haugeneder M Lehning M : Intermediate complexity atmospheric modeling in complex terrain: is it right? *Front.* *Earth Sci.* 2024;12:12. 10.3389/feart.2024.1388416

[46] COSMO : Consortium for small sclae modelling. Accessed: Aug. 21,2025. Reference Source

[47] Schaik B van Baruzier A Loyer C :2025; *A novel analytical tool to capture wind profile variability for wind energy assessment: fast, simple, and beyond state-of-the-art in complex Terrain* Submitted: Elsevier Wind Energy Eng Res.

[48] MeteoSwiss : Föhnindex - MeteoSchweiz. Accessed: Aug. 21,2025. Reference Source

[49] Gasser J Schaik BJA van Huwald H : Wind turbine power performance influenced by the high-alpine atmospheric turbulence, temperature, and snow cover. *Manuscr Submitt.* 2025. 10.2139/ssrn.5332855

[50] HaloPhotonics : HALO PHOTONICS|streamLine series - product. halo-photonics. Accessed: Aug. 21,2025. Reference Source

[51] AET : Azienda Elettrica Ticinese. Accessed: Aug. 21,2025. Reference Source

[52] Schaik B Huwald H Lehning M : Resolving three-dimensional wind velocity fields with sequential wind-doppler LiDAR for wind energy in the complex terrain - Gotthard Pass, Switzerland [version 1; peer review: 1 approved with reservations, 2 not approved]. *Open Res Eur.* 2025;5:9. 10.12688/openreseurope.19095.1 41960566 PMC13058581

