# Supplementary material for: Resolving three-dimensional wind velocity fields in complex terrain using sequential wind-Doppler LiDAR, CFD and wind turbine measurements - Gotthard Pass, Switzerland
Source: Open Res Eur. 2026 Mar 23;5:9. Originally published 2025 Jan 15. [Version 3] doi: 10.12688/openreseurope.19095.3 (PMC13058581; doi:10.12688/openreseurope.19095.3)
Supplement: Peer review history

# Reviewer report: Reviewer response for version 3

**DOI:** 10.21956/openreseurope.24750.r71851

**Published:** 2026-04-16

**Authors:** Baoyuan Deng

**Affiliations:** 1 Hunan University, Changsha, China

**I’m leaning toward accepting this paper.**

 Honestly, I think the authors have done a solid job addressing the big concerns from earlier rounds. The idea itself is pretty cool — instead of needing three expensive LiDARs running at the same time, they show you can get similar results with just one, moved around strategically. That’s a big deal for remote alpine sites.

**Why I’m okay with this paper in its present format:**

**1** **）They fixed the matching logic** **. T**hey’ve added eight different checks using their CFD model (HICAR). It’s not perfect, but it’s way more rigorous than before.

**2** **）They’re honest about the limits.** They’re not claiming this works everywhere. They clearly say it’s best for “channelized” valleys and won’t work on super exposed ridges.

**3** **）Real‑world relevance.**The Gotthard Pass case study actually tells us something useful: how northerly vs. southerly winds mess with turbine efficiency in different ways.

**What still bugs me a tiny bit (but not enough to reject):**

**1** **）Winter is missing.** Seven months of the year, that place is buried in snow, which totally changes the wind. no dataset is ever perfect, and they admit this gap openly. Besides, the collected data is open-sourced in zenodo.

**2** **）HICAR could use more checking.** They didn’t do a deep dive comparing the model directly to measurements, but for the purpose of filtering matches, it seems to do the job.

 This paper isn’t a flawless, ready-for-prime-time industrial tool yet. But as a smart, creative approach to studying complex terrain? Definitely worth indexing.

  I recommend to accept this paper.

Is the study design appropriate and does the work have academic merit?

Yes

Is the work clearly and accurately presented and does it cite the current literature?

Yes

If applicable, is the statistical analysis and its interpretation appropriate?

Yes

Are all the source data underlying the results available to ensure full reproducibility?

Yes

Are the conclusions drawn adequately supported by the results?

Yes

Are sufficient details of methods and analysis provided to allow replication by others?

Partly

Reviewer Expertise:

lidar, wind turbine, data processing

I confirm that I have read this submission and believe that I have an appropriate level of expertise to confirm that it is of an acceptable scientific standard.

# Reviewer report: Reviewer response for version 3

**DOI:** 10.21956/openreseurope.24750.r71458

**Published:** 2026-04-07

**Authors:** Feng Guo

**Affiliations:** 1 Shanghai Jiao Tong University, Dongchuan Road, Shanghai, China

The authors have adequately addressed my review concerns, and I now support the manuscript for indexing.

Is the study design appropriate and does the work have academic merit?

Partly

Is the work clearly and accurately presented and does it cite the current literature?

Partly

If applicable, is the statistical analysis and its interpretation appropriate?

Partly

Are all the source data underlying the results available to ensure full reproducibility?

Partly

Are the conclusions drawn adequately supported by the results?

Partly

Are sufficient details of methods and analysis provided to allow replication by others?

Yes

Reviewer Expertise:

Wind resource assessment, wind lidar, wind turbine aero-elastic

I confirm that I have read this submission and believe that I have an appropriate level of expertise to confirm that it is of an acceptable scientific standard.

# Reviewer report: Reviewer response for version 3

**DOI:** 10.21956/openreseurope.24750.r71459

**Published:** 2026-03-31

**Authors:** Shengming Tang

**Affiliations:** 1 Shanghai Typhoon Institute of China Meteorological Administration, Shanghai, China

I am satisfied with the author responses and have no further questions or comments.

Is the study design appropriate and does the work have academic merit?

Yes

Is the work clearly and accurately presented and does it cite the current literature?

Yes

If applicable, is the statistical analysis and its interpretation appropriate?

Yes

Are all the source data underlying the results available to ensure full reproducibility?

Yes

Are the conclusions drawn adequately supported by the results?

Yes

Are sufficient details of methods and analysis provided to allow replication by others?

Yes

Reviewer Expertise:

Boundary layer meteorology

I confirm that I have read this submission and believe that I have an appropriate level of expertise to confirm that it is of an acceptable scientific standard.

# Reviewer report: Reviewer response for version 2

**DOI:** 10.21956/openreseurope.23094.r64910

**Published:** 2026-02-10

**Authors:** Norman Wildmann

**Affiliations:** 1 Institut für Physik der Atmosphäre, Oberpfaffenhofen, Germany

Van Schaik et al. present an experimental study which utilizes one lidar of type Windcube v2.1 to measure wind profiles at an Alpine pass at different locations. The topic is relevant, since complex terrain flow effects are critical for wind energy siting and many uncertainties remain. In the revised manuscript the authors have included long-term CFD simulations, which I think is absolutely crucial to understand the flow in such complex terrain and interpret the lidar measurements. Filtering the matching situations further with more criteria is a step in the right direction, but I still do not believe that the matching of sequential liar periods is appropriate. I agree with reviewer Nicolas Angelou that there are too many uncertainties in the flow complexity and the lidar measurements in complex terrain to trust the matched data. The manuscript gives a wrong impression that it could be a sound method to combine spatially and temporally separated measurements in complex terrain for site assessment. A few more general comments are given below. The focus of the analyses would have to be significantly changed in my opinion to justify indexing. I recommend to reject the manuscript.

 General comments:

 - I cannot find a response to my initial review. I only find responses to Nicolas Angelou and Shengming Tang. I can thus not fully comprehend if my initial review was incorporated into the revision.

 - I miss an evaluation of the HICAR simulations. How do the statistics from those simulations compare to measurements on site? Which features can be captured and which cannot with the given resolution? The lidar and in situ data could maybe be used to evaluate this, which would make a much more relevant and scientifically sound study.

 - The general research questions and approaches remain very questionable. In the end 35 matches remain for a multi-lidar flow evaluation under different conditions. For site assessment, this is not really suitable.

 - I thus disagree with the last statement: "The sequential LiDAR measurement technique is an excellent candidate to improve wind profile data in the complex terrain without a large amount of meteorological equipment." I think the dataset is a very good candidate for case-study based evaluation of models and development of lidar wind retrievals in complex terrain and should be used and advertised as such.

Is the study design appropriate and does the work have academic merit?

No

Is the work clearly and accurately presented and does it cite the current literature?

No

If applicable, is the statistical analysis and its interpretation appropriate?

Partly

Are all the source data underlying the results available to ensure full reproducibility?

Partly

Are the conclusions drawn adequately supported by the results?

No

Are sufficient details of methods and analysis provided to allow replication by others?

Yes

Reviewer Expertise:

boundary-layer meteorology, lidar measurements, complex terrain, wind energy

I confirm that I have read this submission and believe that I have an appropriate level of expertise to state that I do not consider it to be of an acceptable scientific standard, for reasons outlined above.

# Response

**Published:** 2026-02-13

**Authors:** Brandon van Schaik

We regret to hear that the reviewer was unable to read our responses to the previous comments. Please find the comments below for your convience:   Norman Wildmann - It should be made more clear in the introduction what the actual goal is of the measurements. You could describe what is the standard procedure for WEA, how long the requirements are for instrument deployment and statistics of wind sectors t benchmark your results with. If wind resource assessment is the goal, than I get the impression that the database is much too small and the sequential lidar transects fails to provide the necessary information. If the goal is to study specific flow conditions, I would expect more of a focus on specific situations and some numerical simulations to accompany the measurements. The section title of Methods was misplaced in this draft which meant that a large part of the introduction was actually displayed as the start of the introduction. We have indicated this to be changed to the correct position in the next iteration. Regarding the clearity of the goals, it is **addressed in the manuscript as such:** “This work serves as a proof of concept and as a preliminary showcase of the unique data sets and analyses that are unlocked by the sequential LiDAR experimental methodology, enabling the high-resolution study of the three-dimensional wind over the entire wind park domain.”. In response to this comment, and those made by another reviewer, we have complemented the LiDAR observations with additional CFD simulations covering the full 3-month campaign period and the entire mountain pass. These simulations provide spatially and temporally continuous wind fields that support the short-term measurements. Specifically, we have identified 8 characteristic features including, global horizontal irradiance, atmospheric stability, vertical shear magnitude, variance of the vertical turbulence, an average of the transect wind field, and the synoptic wind at 5km. To this end, the Methodology section is completely overhauled and Results updated accordingly. We believe this addresses the reviewer’s concern by providing a more complete picture of the wind conditions across all sites.

 - The experimental setup is flawed from my perspective because there is no reference to benchmark the results with. I think it would have been important to have at least one lidar continuously at one position. A CFD model to simulate specific wind conditions and check if matching different situations is appropriate could also be applied, but is not provided. Atmospheric stability is not measured at all, but is crucial. Thanks to your suggestions we have opted to provide additional CFD simulations to help contextualize the turbine wind vector matching algorithm. We have updated the matching algorithm as elaborated in the previous comments. We refer to the Methodology section of the manuscript for the detailed overview of the 8 characteristic features that are now implemented.

 - The method section is not well introduced and the purpose for the whole wind speed matching remains very unclear when it is first introduced and is not well explained. The equations are unprecise and variable names are not well introduced. To make this transition to the Methodology more clear, as well as to introduce the idea of the wind speed matching immediately, we have moved the LiDAR details to the instrumentation section. We have improved equations and variables and completely overhauled the Methodology section to include the 8 CFD characteristic features. We believe this overhaul has greatly improved the flow of this section.

 - The way I understand the matching algorithm, two situations are considered the same if wind vectors are within a certain margin, but that is not a full description of the flow. We have updated the matching algorithm as elaborated in the previous comments. We refer to the Methodology section of the manuscript for the detailed overview of the 8 characteristic features that are now implemented. - Figures are of bad quality (low resolution). The captions are not describing the figures well. Figure quality has been addressed. We are limited to 15 words per caption by the journal, we have optimised captions within this limit.

 - Equations are not well explained and variables not described. We have checked all equations and verified that variables are introduced and described.

 - The structure of the manuscript is not consistent jumping back and forth between methods, results, definition of goals. There is no clear story-line. We have updated the matching algorithm as elaborated in the previous comments. We refer to the Methodology section of the manuscript for the detailed overview of the 8 characteristic features that are now implemented.

 - Many statements and references are unprecise and sometimes even wrong. The authors should carefully revise the text. We assume said statements and references are described in the reviewer’s specific comments. We have addressed those below.

 - Drawing conclusions from wind profiles that are put together from different days and conditions, just matched by wind speed at hub height can be extremely misleading. We have updated the matching algorithm as elaborated in the previous comments. We refer to the Methodology section of the manuscript for the detailed overview of the 8 characteristic features that are now implemented.

 - The manuscript is provided in a format which is hard to review (2 columns, no line numbers, etc.). That is maybe not the authors fault, but editors should consider to change the format.  We thank the reviewer for pointing this out. The manuscript format is determined by the journal’s submission system, and unfortunately we do not have direct control over line numbering or layout. We trust that the editorial office will take this into consideration.

 Specific comments:

   p.4, paragraph 1: There are clear definitions for terrain complexity in the IEC 61400. It is probably good to elude a bit on that in the introduction and make clear that the complexity in this study is of the highest complexity (I assume)

 We appreciate the reviewer’s suggestion. While the IEC 61400 provides clear definitions for terrain complexity, it is not an open-access document, and therefore we have chosen not to cite it directly. Nonetheless, we agree that the terrain in our study area qualifies as highly complex according to commonly used classifications. We have clarified this in the revised introduction to better frame the context of our work, without relying on proprietary standards. **Addressed in the manuscript as such:** “The Gotthard Wind Park can be categorised as the highest degree of complex terrain for wind energy development, characterised by large elevation changes, up- and downwind valleys and steep escarpments within the wind park itself.” p.4, paragraph 1: "influencing the performance of wind turbines by reducing or augmenting their efficiency". That sentence is not giving much information

**Addressed in the manuscript as such:** “influencing the performance of the wind turbine by the non-idealised wind flow into the turbine.” p.4, 2nd column 2nd paragraph: "airborne lidar" - that is not correct. There was no airborne lidar measuring wind transects across the valley at Perdigao. We thank the reviewer for catching this mistake. The reference to “airborne lidar” was incorrect and has been removed. It was a confusion with the airborne surface scanning LiDAR used at Perdigão. p.4, 2nd column 2nd paragraph: simulations cannot prove the validity of lidar measurements and were not used for that purpose in the referenced studies. **Addressed in the manuscript as such:** “establishing the use of LiDAR measurements as a valid wind assessment tool over a wide range of spatial resolutions.” p.4, 2nd column 3rd paragraph: WEAs operate lidars to perform WEA? that is a weird construction

 “WEAs rarely operate more than one LiDAR to perform a wind energy assessment.” **Replaced by:** “WEA studies rarely operate more than one LiDAR” p.7, 2nd column "Lidar details": The range gate increments was already introduced in the chapter before. It may make sense to move this section before the explanation of the sequential Instrumentation overview. 

 Thank you for the helpful suggestion, we have adopted this in the revision. p.9, 1st column, 2nd paragraph: why is the temperature sensor at 1m? Usually 2m is a standardized height for such measurements.

 Out of practical reasons this was done. A one metre rebar pole could be easily hammered into the soil. Longer poles to mount the sensor at 2m would require guywires which was not always possible in the terrain. Since the LiDAR was moved every week, this was not practical. Since the surface topography is free of brushes and rocks, this difference should not impact the variances in the temperature readings much. Particularly since we average over 10min intervals. Since the temperature is only used for air density calculations in which the temperature only has a small sensitivity, we do not see a reason to distrust the accuracy of this data. **Addressed in the manuscript as such:** “A radiation-shielded combined pressure, temperature, and humidity sensor was placed near the LiDAR 1 m above the ground which is solely used to calculate the local air density.” p.9, Eq.1 etc.: At this point in the manuscript it is unclear why you want to calculate the difference of all 10-minute wind speeds for the five turbines

 With the additional CFD simulations methods in the manuscript, this has been addressed accordingly. We have also introduced the goals more clearly, and earlier in the manuscript. p.10, Fig.6: the resolution of this figure is very low and texts are hard to read.

 All figures have been replaced with higher-resolution versions in the revised manuscript. In addition, Figure 6 is no longer included, as it became irrelevant following the revisions to the Methodology section and has been removed. p.11, right column, 2nd paragraph: pressure, temperature and humidity are not shown in this manuscript though, right? Which instrumentation is used to check it? Are there any measures for stability? How the flow develops along the transect is surely dependent on stability.

 We only use pressure temperature and humidity to calculate the average air density over the whole campaign. We mention the existence of this sensor as it is included in the data set that is co-published with this paper. The sensor in use is the standard PTH sensor provided by VAISALA for the WindCube v2.1. More details are now given in the instrumentation table (Table 1). Atmospheric stability is now addressed with CFD simulations. We refer the reviewer to our reply of his first comment as well as the Methodology section of the revised manuscript for the full details. p.11, right column, "Results": are Fig.6&7 not already results?

 These figure have become irrelevant following the revisions to the Methodology section and have since been removed. p.11, right column, last paragraphs: why are these paragraphs italic? where do they belong to?

 We thank the reviewer for noting this editing error. The paragraphs are now placed correctly in the revised manuscript. p.13, Table 3: why not show the distributions?

 In our view, the distributions are not directly relevant to the goals of the study. Presenting the results in tabular form provides a more space-efficient and quantitative summary, allowing readers to readily compare the two wind directions as well as reproduce/refer to the data immediately. p.11, "above rated wind speed": the wind roses do not distinguish between above and below rated wind speed, the upper bin is >8m/s, but rated wind speed is 11m/s.

 Excellent suggestion, the wind roses are updated to reflect the different stages in the E-92 power curve, and the figure has been updated. p.12, Figure 8: What are turbines A-E? So far they were referenced with numbers 1-5 or T1-T5. Please be consistent or describe in the caption.

 Combined with the previous comment, we have adapted this as well in the figure. p.13, Figure 9: The caption should be more descriptive about what the colors and arrows mean.

 We are limited to 15 words per figure/table caption by the journal regulations. We have described this in more detail in the text. We have also added a legend to explain the difference in colours. Besides, this figure has been replaced due to the improved methodology as suggested in your earlier comments. We refer the reviewer to the Transect wind profile evolution and Wind shear and veer subsections in the results for our improved results. p.13, right column, first paragraph: If you see a flow acceleration between the position, how can you be sure that acceleration effects are not also present at hub height and would this not be a problem for the wind matching? You actually match conditions which are very different. We have updated the matching algorithm as elaborated in the previous comments. We refer to the Methodology section of the manuscript for the detailed overview of the 8 characteristic features that are now implemented. This flow acceleration can now be identified using by CFD simulation characteristic features: “Vertical shear magnitude”, “Variance of the vertical turbulence”, and “Transect-averaged wind”. We refer the reviewer to the Transect wind profile evolution and Wind shear and veer subsections in the results for our improved results.

 p.14, Figure 11: The figure clearly shows that for positions L4 and L8 there was strong shear and veer, thus a very different flow condition than for the other positions. How can you put these conditions together? It does not make sense.

 We have updated the matching algorithm as elaborated in the previous comments. We refer to the Methodology section of the manuscript for the detailed overview of the 8 characteristic features that are now implemented. This shear/veer concern can now be identified using by CFD simulation characteristic features: “Vertical shear magnitude”, “Variance of the vertical turbulence”, “Global Horizontal Irradiance”, “Atmospheric stability”, and “Synoptic wind at 5km”. We refer the reviewer to the Transect wind profile evolution and Wind shear and veer subsections in the results for our improved results. p.14, right column, last paragraph: I cannot believe that the shear and veer at a specific location is independent of atmospheric stability. 

 We have updated the matching algorithm as elaborated in the previous comments. We refer to the Methodology section of the manuscript for the detailed overview of the 8 characteristic features that are now implemented. This shear/veer and atmospheric stability concern can now be identified using by CFD simulation characteristic features: “Vertical shear magnitude”, “Variance of the vertical turbulence”, and “Atmospheric stability”. We refer the reviewer to the Transect wind profile evolution and Wind shear and veer subsections in the results for our improved results. p.15, Table 4: the table shows that wind profiles of early morning, early afternoon and late afternoon are mixed.

 We have updated the matching algorithm as elaborated in the previous comments. We refer to the Methodology section of the manuscript for the detailed overview of the 8 characteristic features that are now implemented. This time of day mixing can now be identified using by CFD simulation characteristic features: “Global Horizontal Irradiance”, and “Atmospheric Stability” We have consequently updated the table to address the stricter matching protocol discussed in the overhauled Methodology section. p.17, Fig.14: what are the axis of those TKE distribution plots? 

 We have revised the captions to clarify that the plots represent probability density functions of the TKE. The as the exact values of TKE are not directly relevant for our conclusions, and for clarity we therefore choose to not include x ticks or y ticks. Since our caption size is limited, we have updated the manuscript section in which the figure is first referred to as shown below. The plots are intended to highlight relative differences, and since all TKE distributions share identical x-limits, they can be visually intercompared as discussed in the manuscript. **Addressed in the manuscript as such:** “This bi-model distribution is similar to the wind speed, along the LiDAR transect as shown in Figure 12. In these figures, the red lines represent the probability density functions of the TKE, while the blue vertical lines indicate the mean TKE, allowing for a visual identification of where turbulence is strongest along the transect.” p.16, "Turbulence kinetic energy": This Section is premature. You cannot make statistics of TKE with this database and calculate effects on power production the way it is done here. The references are wrong too. Reference 8 does not provide Eq. 8. I recommend to have a close look at the IEC 61400 standards.

 We thank the reviewer for this valuable comment. We have revised the section to clarify that our analysis is not intended as a full turbulence kinetic energy (TKE) assessment, but rather as a simplified approach to provide a rough upper bound on the potential influence of turbulence on turbine performance. We now emphasize that Eq. (8) is a first-order approximation based on the curvature of the power curve and assumes symmetric fluctuations up to second order. Furthermore, we have verified the applicability of Eq. (8) using our 1-second LiDAR data, which shows that the deviations from the mean are approximately symmetric and can be fitted by a Student-t distribution. This supports the use of Eq. (8) as a conservative upper-bound rather than a precise quantification. Finally, we have corrected the references and added a note pointing the reader to the IEC 61400 standards, which provide the proper framework for turbulence intensity and load assessments. **Addressed in the manuscript as such:** “Equation 8 provides a first-order estimate of how turbulence around the mean affect turbine power output: Equation 8 *P* *turbulence*= *P* *v*+ *σ* *h* *orz*+ *P* *v*- *σ* *h* *orz* *2*- *P* *v*= *3* *2* *ρ* *A* *v* *σ* *h* *orz* *2* *>0*   However, the actual effect of turbulence is governed by the curvature of the turbine power curve: In convex sections of the power curve, positive and negative fluctuations are weighted asymmetrically, leading to a net increase in power relative to the mean wind speed. Whereas in concave sections, the reverse occurs and fluctuations produce a net decrease in power. Therefore Equation 8 represents only a rough upper-bound to the influence of turbulence on power production. It does not account for the changing curvature of the real power curve, nor for higher-order asymmetries in the wind speed distribution. For a full quantification, we refer the reader to a work that integrates the measured wind speed probability density function and other vertical variations with the actual turbine power curve as referred to in international standards. Furthermore, we have validated the assumption of second-order symmetry in the wind speed variations measured the Doppler wind LiDAR. The 1-second wind speed data from the WindCube during the campaign shows that the deviations from the mean are not perfectly normally distributed. They rather remain symmetric up to second order and an be fitted optimally with a Student-t distribution ( *ν*=2.88±0.02, *R*2=0.996 ), which captures the heavy tails observed. From this the mean horizontal deviation can be expressed as *σ* *horz*= *ν*/( *ν*-2) =1.81 , Which seems to be independent of height. Which consequently validates that the rough upper-bound still holds.” p.19, Fig.15: What is the normalized power production. There should be an equation somewhere.

**Addressed in the manuscript as such:** “The power production is normalised to 7 ms^(-1) 7 m/s by the cubic relation between power and wind speed in this range with Equation 9. Equation 9 *P* *normalised* *=P* *v* *C* *p* *(7m* *s* *-1* *)* *7 m* *s* *-1* *3* *C* *p* *(v)* *v* *3* *, 4 m* *s* *-1* *≤v≤10 m* *s* *-1*  “ p.19, "Sequential vs. Synchronized": This section does not give any new information compared to the introduction and an actual comparison cannot be made, because data from synchronized lidars is not available in this campaign.

 We have conducted additional CFD simulations to address this concern, as outlined in our earlier responses. We believe that these methodological adaptations improve the robustness of the results and provide sufficient support for our conclusions in this section. p.19, "Conclusion": "a novel method for high-resolution, three-dimensional wind profile measurements" - I disagree. The data presented here is not "high-resolution three-dimensional" It is standard wind profiles with Windcube v2.1. for short time periods at different locations.

 We thank the reviewer for this comment. While we agree that standard WindCube v2.1 instruments provide vertical wind profiles at single locations, our methodology goes beyond this typical use. By deploying the lidar sequentially along a dense transect with 150 m horizontal spacing and 10–20 m vertical resolution, we obtain a high-resolution, three-dimensional reconstruction of the wind field across complex terrain. Something that cannot be achieved with a single lidar in a fixed location. Furthermore, the newly added CFD simulations now support and address the reviewer’s concerns about temporal discontinuity and the matching algorithm. Therefore, we believe that the description of the method as "high-resolution, three-dimensional wind profile measurements" is appropriate in the context of this study. p.20, second paragraph: I disagree that the matching algorithm is successful. The analyses show that just matching the wind speed at hub height is not enough to describe a common flow situation in such complex environment. Actually it is misleading to suggest it does. The newly added CFD simulations help address the reviewer’s concerns regarding temporal discontinuity and provide validation for the adapted matching algorithm. We therefore consider the revised algorithm to be appropriate and effective for the purposes of this study.

# Reviewer report: Reviewer response for version 2

**DOI:** 10.21956/openreseurope.23094.r64909

**Published:** 2026-01-21

**Authors:** Nikolas Angelou

**Affiliations:** 1 Technical University of Dermark (DTU), Frederiksborgvej, Denmark

I would like to thank the authors for their thorough responses to the questions and comments raised in the first version of this manuscript. The revised paper is well-structured, and I appreciate the detailed explanation of the methodology as well as the additional effort to strengthen the analysis through 3D simulations of the wind field.

 However, despite these positive aspects, I think there remain some fundamental weaknesses in the study.

**Specific comments:**

1. The proposed method for matching wind profiles obtained from wind lidar profilers  relies on reference measurements (in the initial manuscript) and, in the revised version, on reference measurements and CFD data. This raises concerns about the applicability of the method for wind resource assessment, as it requires pre-existing wind turbines and additionally, the accuracy of CFD simulations in complex terrain is uncertain. Furthermore, in the updated manuscript, where CFD data are used, there is no explanation of the rationale behind the selection of the eight parameters used.
2. The accuracy of ground-based wind lidar profilers in measuring mean wind speed depends on the assumption of spatial flow homogeneity. In the revised manuscript, CFD data are used to support the hypothesis that the flow distortion at the locations where the wind lidar profiler was installed, is minimal. However, this conclusion depends on the reliability of the CFD simulation. Furthermore, even if this assumption holds for the specific site studied, flows in complex terrain are inherently inhomogeneous. Inhomogeneities that are even stronger when the lidar’s measurement volume includes turbine wakes (as the case for example of the L9 profile in Figure 11). This will introduce a bias in estimating the mean wind vector and would have an even greater impact on the accuracy of second-order moments derived from lidar measurements.
3. The authors state that their intent is not to present a climatological analysis but to investigate spatial flow variability. However, it is unclear how spatial variability can be decoupled from temporal variability. While CFD simulations could provide insight, this would require validation demonstrating the model’s accuracy in predicting wind profiles for the complex terrain under study.
4. In the section *“Gotthard sequential LIDAR transect experiment”*, it is stated that HICAR “can resolve terrain-induced flow features at the hectometer scale.” Later, in *“Instrumentation overview”*, the resolution is given as 50 m for the HICAR v1.1 model. Regardless of which value is correct, I am concerned whether this spatial resolution is sufficient to estimate the expected bias in mean wind speed caused by spatial inhomogeneities.
5. In the section *“LIDAR details”*, the manuscript mentions “five pulsed laser beams, which are magnetically controlled by the instrument.” Could the authors clarify what is meant by “magnetically controlled”? Additionally, the text states that the “return signal… 30 mW mean power laser is recaptured.” Is this accurate?

Is the study design appropriate and does the work have academic merit?

No

Is the work clearly and accurately presented and does it cite the current literature?

Partly

If applicable, is the statistical analysis and its interpretation appropriate?

No

Are all the source data underlying the results available to ensure full reproducibility?

Partly

Are the conclusions drawn adequately supported by the results?

No

Are sufficient details of methods and analysis provided to allow replication by others?

Yes

Reviewer Expertise:

Wind Lidar, Wind Energy, Atmospheric Boundary Layer

I confirm that I have read this submission and believe that I have an appropriate level of expertise to state that I do not consider it to be of an acceptable scientific standard, for reasons outlined above.

# Response

**Published:** 2026-01-30

**Authors:** Brandon van Schaik

IMPORTANT NOTE: Your review was received after resubmission of the manuscript. Due to the current system, we are unable to resubmit the changes yet until the latest version is online. Since we have had the time to address your comments below, we believe it is better to already reply to you. If you provide us your e-mail we can send you the updated manuscript with track changes for validation. We apologize for this inconvenience but it is out of our hands as authors. Nikolas Angelou I would like to thank the authors for their thorough responses to the questions and comments raised in the first version of this manuscript. The revised paper is well-structured, and I appreciate the detailed explanation of the methodology as well as the additional effort to strengthen the analysis through 3D simulations of the wind field. However, despite these positive aspects, I think there remain some fundamental weaknesses in the study. We would like to thank you for your second review of our manuscript. Before answering the specific comments below, it is important to note that we have already submitted a third version including the comments of reviewers Shengming Tang and Feng Guo, which was probably not available to you yet at the point of your review. We will therefore submit a fourth version building on that version, rather than the version you have considered. A few changes have already been made based on the other reviewers’ comments. **Specific comments:**

1. The proposed method for matching wind profiles obtained from wind lidar profilers relies on reference measurements (in the initial manuscript) and, in the revised version, on reference measurements and CFD data. This raises concerns about the applicability of the method for wind resource assessment, as it requires pre-existing wind turbines and additionally, the accuracy of CFD simulations in complex terrain is uncertain. Furthermore, in the updated manuscript, where CFD data are used, there is no explanation of the rationale behind the selection of the eight parameters used.

The inclusion of CFD simulations in the revised manuscript was made in direct response to requests from the reviewers (Norman Wildmann and yourself) in the previous review round, who suggested that reference data beyond in situ measurements would strengthen the methodological framework. We therefore find it important to clarify that the current formulation of the method does not rely on CFD simulations as a replacement for measurements, nor does it assume that CFD can accurately reproduce instantaneous wind profiles in complex terrain.   On the contrary, one of the key findings of this study is that CFD simulations in complex terrain often struggle to reproduce the detailed vertical structure of wind profiles at a given moment in time, even though they may perform reasonably well in a long-term, averaged sense. This limitation is explicitly acknowledged and is, in fact, one of the motivations for the proposed approach.   In the present methodology, CFD simulations are not used as direct profile-to-profile references. Instead, they are employed to mitigate temporal inhomogeneity between lidar observations by providing a dynamically consistent flow field from which robust, physically meaningful parameters can be derived. These eight parameters detailed in the methodology are specifically chosen because they are less sensitive to instantaneous mismatches and more representative of the flow characteristics that can be reliably captured by CFD at a given snapshot in time.   Since these eight parameters are discussed in comment #1, #2 and #3 in a similar manner, we will discuss them in detail once here:

1. GHI – this is an obvious proxy for radiative forcing and cloud cover. GHI varies smoothly in space and time and is largely insensitive to small-scale turbulence or local flow distortions. The HICAR model is based on COSMO-1 (1km resolution, 81 vertical layers) weather model, which is generally reasonably good at estimating this value even in the complex terrain.
2. Atmos. Stabil. Is based on the gradient of the levels from 10 to 247m, and is therefore a bulk thermal stratification of the lower atmosphere. Since it is based on temperature, humidity and pressure that vary smoothly over local terrain at these elevations, it is reasonable to take a single horizontal grid column to base this information from and use it for the local couple hundred meters of the pass top. Additionally, CFD models are generally more reliable at reproducing the bulk stability than the fine-scale velocity fluctuations, making it more robust than a CFD wind profile.
3. Vertical shear magnitude, this is derived form the horizontal wind vectors over a 2km transect which covers tens of grid cells and averages the bulk shear from 10 to 247m. This averaging reduces sensitivity to local topographic effects. Rather than attempting to match the exact shape of the wind profile, this parameters characterises the overall momentum redistribution with height. This flow property is most consistent than a mere single column CFD cell.
4. Variance of the vert. turb., again we average over a 2km transect as well as the 10 to 247 m heights. This metric suppresses any sensitivities to individual turbulent eddies or short-lived local events. While CFD may not accurately reproduce instantaneous turbulence structure as the reviewer suggests, it can capture the relative differences in TI associated with the stability, shear, and terrain-induced forcing.
5. And,
6. Synop. Wind components are derived from level 20 of HICAR at approx. 5km elevation and represents the large-scale forcing of the lfow. At this altitude, the wind is largely decoupled from local terrain effects and surface-layer turbulence. These are essentially direct values taken from COSMO-1, which is generally considered to be accurate at this elevation.
7. And,
8. Transect-averaged wind components are derived from levels 3-5 (61-136m) are used as a proxy for the bulk flow in the rotor relevant layer. Spatial averaging over the 2km transect again helps reduce the influence of localised flow acceleration ,separation, or recirculation induced by the individual complex terrain effects. Thus reflecting the dominant flow orientation and strength experienced by the wind turbines rather than point-scale variability.

Overall, these eight parameters are concretely averaged, Gotthard-wind-park-scale single value parameters that serve as a physically interpretable meteorological overview that is significantly more robust than CFD wind profiles. The revised manuscript now explicitly explains the rationale behind this selection in the methodology section, emphasizing that these parameters are better suited for comparative analysis than full profile matching. For example in the third version of the manuscript (which was already submitted, but not available for you during review), the title has been adapted to better reflect this new method, an additional paragraph to introduce these CFD parameters better, and the conclusion has improved to reflect on the importance of the CFD parameters in the methodology. The introduction now better reflects the scope, limitations, and intended applicability of the method in a version that was already resubmitted before your review was submitted. These revisions were specifically aimed at clarifying that the approach is designed for profile matching and flow characterization, rather than as a standalone wind resource assessment tool requiring pre-existing turbines.   We hope this clarification resolves the reviewer’s concern and better highlights the intended role of CFD simulations within the proposed framework.   Concretely, we have improved the rationale for the CFD simulations in the methodology to make the transition from wind turbine data to CFD data more obvious and logical:

*“HICAR characteristic features* *Building on the nacelle-based matching, we refine the inter-comparison by introducing eight characteristic parameters derived from the HICAR v1.1 mesoscale model. The model provides wind fields on a 50 m horizontal grid with 32 vertical levels, which were interpolated to the nacelle positions (98 m a.g.l.), and to the ten LiDAR locations with their respective range gates.* *In the complex terrain, instantaneous wind profiles are strongly influenced by transient turbulence and local flow distortions, which limits the applicability of direct profiles matching. Numerical model output is therefore only used here to provide a dynamically consistent description of the ambient meteorological conditions at the time of observation that are valid for the Gotthard wind park domain.* *HICAR is not employed to reproduce individual wind profiles, but to derive a small set of averaged, physically meaningful parameters that are less sensitive to local, and short-lived variability that is common in this terrain, and rather difficult to simulate accurately with wind models.* *Alongside the five local wind turbine nacelle wind measurements, we define eight parameters that characterise the inflow conditions from the Gotthard wind park domain, which is chosen to be around Turbine 4 as it is the most central turbine:”*  

1. The accuracy of ground-based wind lidar profilers in measuring mean wind speed depends on the assumption of spatial flow homogeneity. In the revised manuscript, CFD data are used to support the hypothesis that the flow distortion at the locations where the wind lidar profiler was installed, is minimal. However, this conclusion depends on the reliability of the CFD simulation. Furthermore, even if this assumption holds for the specific site studied, flows in complex terrain are inherently inhomogeneous. Inhomogeneities that are even stronger when the lidar’s measurement volume includes turbine wakes (as the case for example of the L9 profile in Figure 11). This will introduce a bias in estimating the mean wind vector and would have an even greater impact on the accuracy of second-order moments derived from lidar measurements.

We thank the reviewer for this detailed comment and agree that ground-based wind lidar profilers inherently rely on assumptions of spatial flow homogeneity within the measurement volume. This limitation is well known and applies to essentially all remote-sensing–based wind measurements in complex terrain.   In the revised manuscript, CFD simulations are not used to claim absolute accuracy of the LiDAR-derived wind statistics, nor are they used to validate the lidar measurements directly. Instead, CFD is employed in a supporting role, using a selection of eight robust flow parameters that are less sensitive to local, instantaneous flow distortions. Importantly, CFD wind profiles themselves are never used directly in the matching algorithm. This distinction is critical and is now clarified more explicitly in the methodology. (details on the eight parameters in the previous comment response)   Regarding flow distortion at the lidar locations, we fully agree that any conclusion drawn from CFD is conditional on the reliability of the simulation. For this reason, we deliberately avoid relying on point-wise CFD wind speeds and instead focus on bulk flow characteristics that are known to be more robust in complex terrain. The CFD results are therefore used to exclude strong, systematic flow distortion, not to assert perfect flow homogeneity.   The reviewer is also correct that flows in complex terrain are inherently inhomogeneous and that this inhomogeneity is exacerbated when the LiDAR measurement volume intersects turbine wakes (e.g., the L9 profile in Figure 11). This will inevitably introduce biases in the estimated mean wind vector and, even more so, in second-order moments such as turbulence intensity. However, this limitation is not specific to the present study, but rather reflects the current state of the art in wind energy field measurements. Volume averaging and 10-minute statistics are standard practice in both research and industry, particularly in terrain where erecting multiple 100+ m meteorological towers is not feasible.   With respect to second-order moments, we emphasize that the turbulence quantities used in this study are 10-minute averaged values, consistent with standard wind energy assessment methodologies. While these cannot capture the full complexity of turbulence that would be measured by dense arrays of sonic anemometers, such measurements are practically impossible at multiple sites across a mountain pass. The presented approach therefore represents the best achievable compromise between spatial coverage and measurement fidelity under realistic field conditions.   Finally, we note that a follow-up study by the authors (currently available as a preprint here: https://wes.copernicus.org/preprints/wes-2025-271/) uses the lidar-derived 10-minute mean wind speed and turbulence intensity obtained with the present methodology to predict 10-minute cumulative wind turbine power output, showing remarkably good agreement with observations. This independent result provides strong evidence that, despite the acknowledged limitations, the derived wind statistics are physically meaningful and representative of the flow relevant for wind energy applications.   We have added further clarification in the revised manuscript to explicitly acknowledge these limitations and to better position the method within the context of current best practices in complex-terrain wind measurements:   In the results section, Turbulence Kinetic Energy:

*“We preface this subsection by noting that turbulence quantities derived from Doppler wind LiDAR measurements are inherently influenced by volume averaging along the laser beam and over the sampling volume, which particularly affects second-order moments such as variance and, consequently, turbulence kinetic energy. As a result, the absolute magnitude of TKE should not be interpreted as a point-scale turbulence intensity in the classical sense, but rather as a relative measure for comparing different flow regimes along the transect. Despite this limitation, scanning LiDAR remains the state of the art for capturing spatial variability in turbulence over complex terrain, as the deployment of tall meteorological masts at comparable heights and at multiple locations along a 2 km transect is not practically feasible.”*   And in the Conclusions section: *“…. A qualitative assessment of the compound effects of complex terrain on the incident wind agrees well with the turbine data. The applicability of the presented approach may be reduced at exposed hilltop or ridge locations, where strong flow inclination and separation can violate the homogeneity assumptions required for ground-based Doppler LiDAR measurements. This interpretation should be understood in a relative sense, as turbulence metrics derived from Doppler LiDAR are affected by volume averaging and therefore describe spatially coherent variability rather than point-scale turbulence intensity.”*  

1. The authors state that their intent is not to present a climatological analysis but to investigate spatial flow variability. However, it is unclear how spatial variability can be decoupled from temporal variability. While CFD simulations could provide insight, this would require validation demonstrating the model’s accuracy in predicting wind profiles for the complex terrain under study.

The intent of this study is not to achieve a strict mathematical separation of spatial and temporal variability, but rather to reduce the influence of temporal variability sufficiently to allow a meaningful investigation of relative spatial flow variability across measurement locations. This distinction is important.   In practice, temporal variability is mitigated through a combination of: (i) simultaneous multi-profile lidar measurements, (ii) the use of time-averaged (10-minute) statistics consistent with standard wind energy practice, and (iii) the use of CFD-derived flow descriptors that are robust to instantaneous fluctuations and do not rely on exact profile matching. (as explained in the response to comment #1)   As emphasized throughout the revised manuscript, CFD simulations are not used to reproduce instantaneous wind profiles and are therefore not required to accurately predict profile shapes at a given moment in time. Instead, CFD provides a dynamically consistent reference flow that helps identify large-scale spatial patterns and supports the interpretation of observed variability. The parameters extracted from the CFD simulations are chosen specifically because they are less sensitive to short-term temporal variability and known limitations of CFD in complex terrain.   We fully agree that CFD-based prediction of exact wind profiles in complex terrain would require extensive validation, which is beyond the scope of this work. However, such validation is not a prerequisite for the present analysis, as the CFD simulations are not used in a prognostic or climatological sense, nor as a direct reference for the lidar measurements.   In line with comment the two comments above, we have further clarified this point in the revised manuscript by explicitly stating that the analysis aims to minimize, rather than eliminate, temporal variability in order to examine spatial flow variability under realistic field conditions. *“….”*

1. In the section “Gotthard sequential LIDAR transect experiment”, it is stated that HICAR “can resolve terrain-induced flow features at the hectometer scale.” Later, in “Instrumentation overview”, the resolution is given as 50 m for the HICAR v1.1 model. Regardless of which value is correct, I am concerned whether this spatial resolution is sufficient to estimate the expected bias in mean wind speed caused by spatial inhomogeneities.

The term “hectometer-scale” refers to the target application scale of the HICAR model and does not imply a fixed grid spacing of 100 m. In the present study, the HICAR v1.1 simulations are performed at a horizontal resolution of 50 m, as stated in the “Instrumentation overview” section. This resolution lies within the hectometer scale and is consistent with the design of the HICAR model.   Regarding the reviewer’s concern about whether this spatial resolution is sufficient to estimate potential biases in mean wind speed due to spatial inhomogeneities, we note that this question has been explicitly addressed in previous studies. In particular, the two references already cited in the manuscript demonstrate that resolutions on the order of 50–100 m are adequate to capture terrain-induced flow features relevant for assessing lidar volume-averaging effects and associated biases in complex terrain: https://doi.org/10.5194/gmd-16-5049-2023, https://doi.org/10.3389/feart.2024.1388416   We have revised the text to more clearly state that a 50 m horizontal resolution is used throughout this study, and to avoid any potential misunderstanding arising from the terminology.  

1. In the section “LIDAR details”, the manuscript mentions “five pulsed laser beams, which are magnetically controlled by the instrument.” Could the authors clarify what is meant by “magnetically controlled”? Additionally, the text states that the “return signal… 30 mW mean power laser is recaptured.” Is this accurate?

The above-mentioned excerpts are direct mentions of the WindCube’s manual that accompanied the instrument that was delivered by VAISALA. Therefore, we assume that the 30 mW mean laser power is accurate, and that the laser beams are magnetically controlled. The manual does not state exactly how this magnetic controller works exactly, but after some research on this topic this likely has to do with how a strong magnetic field can influence the reflection conditions of the prism such that the beam is reflected in one of the 5 directions; vertical, or in one of the four cardinal directions.

 Since this information is not essential for the understanding of wind energy in the complex terrain, we elect to not elaborate this any further in the manuscript, however we will include a citation to the exact manual this information this was taken form.

# Reviewer report: Reviewer response for version 2

**DOI:** 10.21956/openreseurope.23094.r64908

**Published:** 2026-01-06

**Authors:** Shengming Tang

**Affiliations:** 1 Shanghai Typhoon Institute of China Meteorological Administration, Shanghai, China

Thank you for your thoughtful revision. Regarding my second point concerning the vertical variation of wind direction at L8, I wish to clarify that my focus was specifically on wind direction changes with height​ (vertical profile), not on lateral deflection over a horizontal distance. The common expectation of wind veering with height in the Northern Hemisphere stems from the Ekman spiral​ in the planetary boundary layer—a theory fundamentally driven by the Coriolis force balancing friction over hundreds of meters vertically. Therefore, the observed wind rotation at L8 is primarily driven by local topography, which overwhelms and distorts the expected background veering pattern. I suggest the manuscript could briefly acknowledge the classical Ekman/Coriolis framework as the baseline atmospheric expectation that is being locally disrupted. This would provide a more complete and pedagogically sound explanation for readers.

Is the study design appropriate and does the work have academic merit?

Yes

Is the work clearly and accurately presented and does it cite the current literature?

Yes

If applicable, is the statistical analysis and its interpretation appropriate?

Yes

Are all the source data underlying the results available to ensure full reproducibility?

Yes

Are the conclusions drawn adequately supported by the results?

Yes

Are sufficient details of methods and analysis provided to allow replication by others?

Yes

Reviewer Expertise:

Boundary layer meteorology

I confirm that I have read this submission and believe that I have an appropriate level of expertise to confirm that it is of an acceptable scientific standard, however I have significant reservations, as outlined above.

# Response

**Published:** 2026-01-16

**Authors:** Brandon van Schaik

Shengming Tang

 Thank you for your thoughtful revision. Regarding my second point concerning the vertical variation of wind direction at L8, I wish to clarify that my focus was specifically on wind direction changes with height​ (vertical profile), not on lateral deflection over a horizontal distance. The common expectation of wind veering with height in the Northern Hemisphere stems from the Ekman spiral​ in the planetary boundary layer—a theory fundamentally driven by the Coriolis force balancing friction over hundreds of meters vertically. Therefore, the observed wind rotation at L8 is primarily driven by local topography, which overwhelms and distorts the expected background veering pattern. I suggest the manuscript could briefly acknowledge the classical Ekman/Coriolis framework as the baseline atmospheric expectation that is being locally disrupted. This would provide a more complete and pedagogically sound explanation for readers.

**Response**: We thank the reviewer for clarifying their comment. We agree that acknowledging the referred framework is beneficial. We believe, the best location for this would be the Results section: “Windshear and veer”. We address this in the manuscript by adding the following paragraph in this subsection: *“Under idealized conditions in the planetary boundary layer, wind direction is expected to veer with height in the Northern Hemisphere as a result of the Ekman balance between surface friction and the Coriolis force. This Ekman-induced veering typically develops gradually over several hundred meters in the vertical. At L8, however, the observed wind direction changes with height strongly deviate from this classical framework and are instead dominated by local topographic forcing, which overwhelms and distorts the background Ekman veering.”*

# Reviewer report: Reviewer response for version 2

**DOI:** 10.21956/openreseurope.23094.r65117

**Published:** 2025-12-30

**Authors:** Feng Guo

**Affiliations:** 1 Shanghai Jiao Tong University, Dongchuan Road, Shanghai, China

The manuscript entitled *“Resolving three-dimensional wind velocity fields with sequential wind Doppler LiDAR for wind energy in complex terrain – Gotthard Pass, Switzerland”* aims to characterize the wind conditions over the complex terrain of the Gotthard Pass in Switzerland. The study provides valuable insights into the wind characteristics driven by topographically channelled flow in this region, thereby supporting future decisions in wind farm design under similar conditions. The work demonstrates strong engineering merit; however, several improvements are recommended.

 1. The manuscript title does not clearly reflect the methods applied in the study. The proposed approach requires CFD simulations in combination with nacelle anemometer measurements. However, the title gives the impression that wind lidar alone is sufficient to obtain the three-dimensional wind velocity distributions, which is not accurate.

 2. Although the current study is conducted in complex terrain, the five turbines are aligned along a channel where the wind behavior is considerably less complicated than at hilltop locations. In hilly areas, flow inclination and flow separation can violate the homogeneous flow assumption required for ground-based lidar measurements. This limitation should be clearly acknowledged and emphasized by the authors.

 3. The quality of the figures—especially Figure 14—needs to be improved. Clearer visualization and higher resolution are recommended.

 4. Some grammatical and language issues are observed. For example, “a complete profile over the swept area by the turbine blade” should be “a complete profile over the swept area by the turbine blades,” and “a blade diameter of 92 m” should be “a rotor diameter of 92 m.” The authors are encouraged to carefully proofread the manuscript to correct similar issues.

 5. LiDAR horizontal flow homogeneity assumption:

*The authors are encouraged to illustrate the horizontal flow homogeneity assumption by presenting the simulated mean flow field in a figure.*

 6. “During snow-covered periods, which are not included in our LiDAR measurement period.”

*Blade icing, which reduces aerodynamic efficiency, may also contribute to performance variations and should be acknowledged by the authors.*

 7. “Conclusion.”

*The authors should refine the conclusion. Based on the results presented, the sequential LiDAR measurement technique appears to be a strong candidate for improving wind profile characterization in channelized wind sites within complex terrain.*

Is the study design appropriate and does the work have academic merit?

Partly

Is the work clearly and accurately presented and does it cite the current literature?

Partly

If applicable, is the statistical analysis and its interpretation appropriate?

Partly

Are all the source data underlying the results available to ensure full reproducibility?

Partly

Are the conclusions drawn adequately supported by the results?

Partly

Are sufficient details of methods and analysis provided to allow replication by others?

Yes

Reviewer Expertise:

Wind resource assessment, wind lidar, wind turbine aero-elastic

I confirm that I have read this submission and believe that I have an appropriate level of expertise to confirm that it is of an acceptable scientific standard, however I have significant reservations, as outlined above.

# Response

**Published:** 2026-01-16

**Authors:** Brandon van Schaik

Feng Guo

 The manuscript entitled “Resolving three-dimensional wind velocity fields with sequential wind Doppler LiDAR for wind energy in complex terrain – Gotthard Pass, Switzerland” aims to characterize the wind conditions over the complex terrain of the Gotthard Pass in Switzerland. The study provides valuable insights into the wind characteristics driven by topographically channelled flow in this region, thereby supporting future decisions in wind farm design under similar conditions. The work demonstrates strong engineering merit; however, several improvements are recommended.  

 1. The manuscript title does not clearly reflect the methods applied in the study. The proposed approach requires CFD simulations in combination with nacelle anemometer measurements. However, the title gives the impression that wind lidar alone is sufficient to obtain the three-dimensional wind velocity distributions, which is not accurate.

**Response: **We thank the reviewer for catching this shift in methodology had not caught up to our manuscript title. We have changed the title from: *“Resolving three-dimensional wind velocity fields with sequential wind-Doppler LiDAR for wind energy in the complex terrain - Gotthard Pass, Switzerland”* To: *“Resolving three-dimensional wind velocity fields in complex terrain using sequential wind-Doppler LiDAR, CFD, and wind turbine measurements – Gotthard Pass, Switzerland”*  

 2. Although the current study is conducted in complex terrain, the five turbines are aligned along a channel where the wind behavior is considerably less complicated than at hilltop locations. In hilly areas, flow inclination and flow separation can violate the homogeneous flow assumption required for ground-based lidar measurements. This limitation should be clearly acknowledged and emphasized by the authors.

**Response: **We agree with the reviewer and have explicitly acknowledged this limitation in the Sequential LiDAR transect subsection. The revised text now states that the turbines are aligned along a channelled plateau with less complex flow than exposed hilltop locations, and that flow inclination and separation in more complex terrain can violate the homogeneity assumption required for ground-based Doppler LiDAR measurements. This is now in addressed in the “Sequential LiDAR transect” subsection in the Field site, instrumentation and modelling Section:

*“… It is noted that, despite the overall complex terrain of the Gotthard Pass, the turbines and LiDAR transect are aligned along a topographically channeled plateau. As a consequence, the flow conditions encountered here are less complex than at exposed hilltop or ridge locations, where flow inclination and flow separation may violate the horizontal homogeneity assumption required for ground-based Doppler LiDAR wind retrievals. This represents a limitation of the present study.”*

 3. The quality of the figures—especially Figure 14—needs to be improved. Clearer visualization and higher resolution are recommended.

**Response: **We believe this is an issue with the typesetting of composite figures as the quality of the figures in our submission folder are much better. We will address this by pre-typesetting the composite figures (especially 14 and 15) and delivering a single high-DPI figure.

 4. Some grammatical and language issues are observed. For example, “a complete profile over the swept area by the turbine blade” should be “a complete profile over the swept area by the turbine blades,” and “a blade diameter of 92 m” should be “a rotor diameter of 92 m.” The authors are encouraged to carefully proofread the manuscript to correct similar issues.

**Response:**We apologize for these issues; we have proof read the manuscript to address such issues as well as the ones mentioned here.

 5. LiDAR horizontal flow homogeneity assumption: The authors are encouraged to illustrate the horizontal flow homogeneity assumption by presenting the simulated mean flow field in a figure.

**Response: **We thank the reviewer for this suggestion and agree that illustrating the horizontal homogeneity assumption would be valuable if meaningful spatial variability could be resolved. We examined the simulated mean flow fields from the HiCAR v1.1 model. However, at its 50 m horizontal resolution, the model does not resolve sub-grid-scale variability over the 60-140m spacing of the individual LiDAR beams that are relevant for the swept are of the turbine blades with the 28° angle from the horizontal. Showing the simulated mean flow does not help to address this as it appears nearly homogeneous across the transect, and a figure would not provide additional insight into the validity of the LiDAR homogeneity assumption and could potentially be misleading.

 Instead, we have clarified this limitation in the manuscript by explicitly stating that the homogeneity assumption cannot be directly illustrated using the available model resolution and must be interpreted as a methodological assumption rather than a resolved flow feature in the subsection “LiDAR horizontal flow homogeneity assumption”:

*“… Additionally, at the horizontal scales relevant for the LiDAR measurements, the HiCAR v1.1 model resolution (50 m) does not resolve sub-grid flow variability within the LiDAR sampling volume. Consequently, model-derived mean flow fields appear horizontally smooth over the transect length and are therefore not well suited to visually illustrate the LiDAR horizontal homogeneity assumption.”*

 6. “During snow-covered periods, which are not included in our LiDAR measurement period.” Blade icing, which reduces aerodynamic efficiency, may also contribute to performance variations and should be acknowledged by the authors.

**Response: **We have acknowledged these points now as follows:

*“During snow-covered periods, which are not included in our LiDAR measurement period, but are partly represented in the longer time domain of Figure 15b, a general decrease in efficiency is observed over all the turbines. In addition to surface-related and atmospheric effects, blade icing during snow-covered periods may further reduce aerodynamic efficiency and contribute to the observed performance decrease. While icing is not explicitly quantified in this study, it is a known factor affecting turbine performance in high-alpine winter conditions. …”*

 7. “Conclusion.” The authors should refine the conclusion. Based on the results presented, the sequential LiDAR measurement technique appears to be a strong candidate for improving wind profile characterization in channelized wind sites within complex terrain.

**Response: **We thank the reviewer for this constructive suggestion and agree that the applicability of the presented method should be more clearly defined. The Conclusion has been refined accordingly to better reflect the scope of the results.

 For example, we now state in the opening sentence that the method applies to *“channelized flow conditions within complex terrain”* rather than complex terrain in general. Furthermore, the concluding paragraph has been revised to emphasise that the sequential LiDAR technique is *“an excellent candidate to improve wind profile characterization in channelized wind sites within complex terrain, where flow alignment and limited separation support the underlying LiDAR measurement assumptions.” *As has been suggested by other comments above. We now also explicitly acknowledge a key limitation by noting that *“the applicability of the presented approach may be reduced at exposed hilltop or ridge locations, where strong flow inclination and separation can violate the homogeneity assumptions required for ground-based Doppler LiDAR measurements.”*

# Reviewer report: Reviewer response for version 1

**DOI:** 10.21956/openreseurope.20665.r50316

**Published:** 2025-02-22

**Authors:** Nikolas Angelou

**Affiliations:** 1 Technical University of Dermark (DTU), Frederiksborgvej, Denmark

The manuscript entitled:” Resolving three-dimensional wind velocity fields with sequential wind-Doppler LiDAR for wind energy in the complex terrain – Gotthard Pass, Switzerland” presents a study of the wind conditions over a complex terrain. The topic is relevant in a wind energy context, since as it is also pointed by the authors, the wind resource assessment over complex terrain is a challenging task that requires wind observations. For this purpose, the authors performed a field study using a ground-based wind lidar profiler, which was sequentially moved to different locations, and developed a methodology to characterise the spatial variability of the wind conditions. Even though the topic is interesting the proposed methodology has the following weaknesses:

1. The wind lidar data set consists of observations acquired in different locations during a 3-month period. During such a period the wind conditions change, therefore it is not straightforward to inter-compare the wind profile acquired over one week in June in one location with the one measured in September in another location. Additionally, the measurement duration at each location varies from 1 to 12 days. This duration is quite limited and further as the authors state the measurements were acquired over different times of the day. Therefore, the measured wind profiles are not necessary characteristic of the wind conditions at the measured locations.   
2. The matching algorithm proposed, which is one of the innovations of this study, is based on grouping the acquired wind lidar data set according to the hub-height wind speed measured by the nacelle-based anemometers. However, similar wind conditions at the hub-height do not necessary correspond to a specific wind profile.  Furthermore, the application of this method requires the measurement of the wind conditions over different locations at the hub height of a wind turbine. Therefore, it cannot be used in the context of a wind resource assessment.
3. There is no discussion in the manuscript about the flow distortion of the nacelle-mounted anemometer and the reliability of the measurement of the longitudinal and transverse component of the horizontal wind vector.
4. The accurate measurement of the wind profile using a ground-based wind profile assumes of horizontal flow homogeneity. An assumption that is not valid over a complex terrain and thus the derivation of the mean wind profile using a ground-based wind lidar profiler is not necessarily correct. This is even more relevant, when wind lidar profiler data are used to estimate the standard deviation of the wind vector components.
5. Based on the locations where the wind lidar measurements were acquired and the examined wind direction sectors, I imagine that at least part of the wind lidar measurements are biased by the wake generated by the wind turbines.

Based on the above points, I think that the statistical analysis performed is not appropriate and cannot support the conclusions drawn. Furthermore, the quality of the figures and the use of certain references should be improved/corrected (for example the reference [10] is not presenting a validation of a CFD simulation).  Therefore, I unfortunately think that the article cannot be considered for indexing in its current version.

Is the study design appropriate and does the work have academic merit?

No

Is the work clearly and accurately presented and does it cite the current literature?

Partly

If applicable, is the statistical analysis and its interpretation appropriate?

No

Are all the source data underlying the results available to ensure full reproducibility?

Partly

Are the conclusions drawn adequately supported by the results?

No

Are sufficient details of methods and analysis provided to allow replication by others?

Yes

Reviewer Expertise:

Wind Lidar, Wind Energy, Atmospheric Boundary Layer

I confirm that I have read this submission and believe that I have an appropriate level of expertise to state that I do not consider it to be of an acceptable scientific standard, for reasons outlined above.

# Response

**Published:** 2025-11-11

**Authors:** Brandon van Schaik

Nikolas Angelou

1. The wind lidar data set consists of observations acquired in different locations during a 3-month period. During such a period the wind conditions change, therefore it is not straightforward to inter-compare the wind profile acquired over one week in June in one location with the one measured in September in another location. Additionally, the measurement duration at each location varies from 1 to 12 days. This duration is quite limited and further as the authors state the measurements were acquired over different times of the day. Therefore, the measured wind profiles are not necessary characteristic of the wind conditions at the measured locations.

We acknowledge that the LiDAR measurements at each site are not necessarily representative of the full seasonal wind variability, nor are they sufficient to define a robust statistical wind distribution or wind rose for each location. However, the intent of this study is not to present a climatological analysis, but rather to investigate spatial variability and flow characteristics during the measurement period. In response to this comment, and those made by another reviewer, we have complemented the LiDAR observations with additional CFD simulations covering the full 3-month campaign period and the entire mountain pass. These simulations provide spatially and temporally continuous wind fields that support the short-term measurements. Specifically, we have identified 8 characteristic features including, global horizontal irradiance, atmospheric stability, vertical shear magnitude, variance of the vertical turbulence, an average of the transect wind field, and the synoptic wind at 5km. To this end, the Methodology section is completely overhauled and Results updated accordingly. We believe this addresses the reviewer’s concern by providing a more complete picture of the wind conditions across all sites.

1. The matching algorithm proposed, which is one of the innovations of this study, is based on grouping the acquired wind lidar data set according to the hub-height wind speed measured by the nacelle-based anemometers. However, similar wind conditions at the hub-height do not necessary correspond to a specific wind profile.  Furthermore, the application of this method requires the measurement of the wind conditions over different locations at the hub height of a wind turbine. Therefore, it cannot be used in the context of a wind resource assessment.

Thanks to your suggestions we have opted to provide additional CFD simulations to help contextualize the turbine wind vector matching algorithm. We have updated the matching algorithm as elaborated in the previous comments. We refer to the Methodology section of the manuscript for the detailed overview of the 8 characteristic features that are now implemented.

1. There is no discussion in the manuscript about the flow distortion of the nacelle-mounted anemometer and the reliability of the measurement of the longitudinal and transverse component of the horizontal wind vector.

Nacelle distortion is an important part when considering nacelle measured wind speed to wind power generated by the wind turbine. In this study we only ever consider nacelle measured wind speeds relative to measurements from the same anemometer in the same location of the same turbine. Therefore, this distortion in not relevant as the same bias is included in all comparisons and cancels out. With the new introduction of CFD simulations, this issue becomes valid in a hypothetical case where we compare CFD simulations to the nacelle measurement. We address in the manuscript that this distortion is present, and that it is not possible to directly compare them in this case. **Addressed in the manuscript as such:** *“We are unable to compare the HICAR wind vectors at the wind turbine nacelles with the in-situ data measured by the anemometers due to the flow distortion of the nacelle which is not simulated.”*

1. The accurate measurement of the wind profile using a ground-based wind profile assumes of horizontal flow homogeneity. An assumption that is not valid over a complex terrain and thus the derivation of the mean wind profile using a ground-based wind lidar profiler is not necessarily correct. This is even more relevant, when wind lidar profiler data are used to estimate the standard deviation of the wind vector components.

With the WindCube V2.1 profiler, the angled lasers are pointed 28° from the vertical. This roughly means that at 100m the N and S beam are ~100m apart. With our newly available CFD simulations we can now explore how much the wind vector has changed at 100m and 300m height with 100m and 300m horizontal spacing respectively in all 4 cardinal directions. Vertical inhomogeneity in both the N-S and E-W direction at corresponding the corresponding height above the LiDAR show the following biases and standard deviations: **100m above LiDAR**

**(100m horizontal spacing)** **Bias [m/s]** **Standard deviation [m/s]** N – S differences in v (dir. north) 0.094 0.116 E – W differences in u (dir. east) 0.002 0.251 N – S & E – W differences in w (dir. up) 0.012 0.058 **300m above LiDAR**

**(300m horizontal spacing)** **Bias [m/s]** **Standard deviation [m/s]** N – S differences in v (dir. north) 0.019 0.392 E – W differences in u (dir. east) -0.085 0.229 N – S & E – W differences in w (dir. up) 0.048 0.230 Biases are all <0.1 m/s and therefore neglectable compared to the error of the LiDAR. Standard deviations are higher and can be explained as follows. In the N-S direction, which is the prevailing direction, you can expect larger changes as you increase horizontal spacing as there is a strong channeling effect. Yet, still errors are within < 0.5m/s which is less than the LiDAR standard measuring error. For E-W direction, the error at lower height is slightly bigger which is likely due to surface effects. Our CFD simulations don’t include thermal winds such as katabatic and anabatic winds due to thermal forcing, therefore errors may be a bit bigger in reality. But still far within reason to assume homogeneity. Finally, there is a small vertical wind speed error as the pass winds have to go up over the pass and then down again. However, with the angle of the laser at 28° from the vertical, the effective error is halved (sin(28°)~=0.5) and therefore also well within reason. As the manuscript contains already a lot of tables and information, we have opted to not show this table in the manuscript and rather address this issue in the following subsection: **Addressed in the manuscript as such:** *“LiDAR horizontal flow homogeneity assumption* *In complex terrain, concerns may arise regarding the applicability of Doppler wind LiDARs, as horizontal flow inhomogeneities can violate the homogeneity assumption underlying the retrieval of vertical wind profiles. The WindCube V2.1 profiler employs four beams inclined 28° from the vertical, corresponding to a horizontal separation of approximately 100 m between opposing beams at 100 m height. To evaluate potential impacts of horizontal inhomogeneity, we analysed CFD simulations across representative distances of 100 m (at 100 m height) and 300 m (at 300 m height) in all four cardinal directions. This shows that the mean biases in all wind components remain below 0.1 ms^(-1), which is negligible relative to the intrinsic measurement uncertainty of the LiDAR. Standard deviations are generally below 0.5 ms^(-1), with slightly larger values in the north–south direction, attributable to valley channeling, and in the east–west direction near the surface, likely related to surface effects. Although the CFD simulations do not represent thermally driven flows such as katabatic or anabatic winds, which may increase variability in reality, the observed deviations are on the order of the intrinsic error of the LiDAR, thus supporting the validity of the horizontal homogeneity assumption. Consequently, LiDAR-derived vertical profiles can be interpreted without additional corrections.”*

1. Based on the locations where the wind lidar measurements were acquired and the examined wind direction sectors, I imagine that at least part of the wind lidar measurements are biased by the wake generated by the wind turbines.

Indeed we do observe some wind profiles in the LiDAR data, particularly for Northerly winds incident on Turbine 2 going to LiDAR 9, that would fit wake distortion. However, this is not a problem to our methods, but rather a feature of the sequential LiDAR transect. We do not use the LiDAR data to create matches, rather we use the matches to show off LiDAR profiles to visualize the transect 3D wind profile. Since wake distortion is an inherent part of the 3D wind field captured along the transect, its presence helps illustrate the spatial extent of the wake, specifically where it emerges and where it dissipates. The CFD simulations in HICAR do not include the wind turbines and thus also do not suffer from this during the matching algorithm.

1. Furthermore, the quality of the figures and the use of certain references should be improved/corrected (for example the reference [10] is not presenting a validation of a CFD simulation).  Therefore, I unfortunately think that the article cannot be considered for indexing in its current version.

  We have addressed figure quality and said reference. 

# Reviewer report: Reviewer response for version 1

**DOI:** 10.21956/openreseurope.20665.r49933

**Published:** 2025-02-12

**Authors:** Shengming Tang

**Affiliations:** 1 Shanghai Typhoon Institute of China Meteorological Administration, Shanghai, China

In this study, a deployment method called sequential wind-Doppler LiDAR deployment is introduced. This method proposes a matching algorithm that can group wind profiles among different LiDARs. The proposed deployment method is novel and practical because only a single wind-Doppler LiDAR is needed to construct the three-dimensional wind field instead of multiple synchronized LiDARs. This is an important work for the observational study of the near-surface wind field and the design and operation of wind power generation in mountainous areas. The structure is well designed, the method is innovative, and the analysis is detailed, comprehensive and thorough, with its conclusions are adequately supported by the results. Although this work has good academic and engineering merits, some issues are still needed to be addressed before publication.

1. Table 1: Please explain clearly how to understand the values of Easting (m) and Northing (m)? If a map projection is used, please explain it in the main text.
2. As shown in Figure 11, for northerly wind, the wind direction of L4 rotates clockwise with the increase of height. However, the wind direction of L8 rotates anticlockwise with the increasing height, which is not consistent with the common sense that the wind direction rotates clockwise with the increasing height in the northern hemisphere. Please explain why.
3. Page 16: Turbulence kinetic energy (TKE) *k* exhibits bi-modal distributions. What is the meaning of ‘bi-modal’?
4. Page 16, Equation 8: The authors assume that the mean wind speed is symmetrical until the second order (e.g. normal distribution). Actually, it is easy to verify whether the wind speed is normally distributed when having the raw data. Please provide the relevant verification process and statistical results.
5. Figures 7, 9, 10, 11 are blurry, please increase the figure resolution.

Is the study design appropriate and does the work have academic merit?

Yes

Is the work clearly and accurately presented and does it cite the current literature?

Yes

If applicable, is the statistical analysis and its interpretation appropriate?

Yes

Are all the source data underlying the results available to ensure full reproducibility?

Yes

Are the conclusions drawn adequately supported by the results?

Yes

Are sufficient details of methods and analysis provided to allow replication by others?

Yes

Reviewer Expertise:

Boundary layer meteorology

I confirm that I have read this submission and believe that I have an appropriate level of expertise to confirm that it is of an acceptable scientific standard, however I have significant reservations, as outlined above.

# Response

**Published:** 2025-11-11

**Authors:** Brandon van Schaik

Shengming Tang

1. Table 1: Please explain clearly how to understand the values of Easting (m) and Northing (m)? If a map projection is used, please explain it in the main text.

**Addressed in the manuscript as such:** “Table 1. Instrumentation and data availability. Easting and Northing coordinates in the Swiss Coordinate system LV95 (EPSG:2056).”

1. As shown in Figure 11, for northerly wind, the wind direction of L4 rotates clockwise with the increase of height. However, the wind direction of L8 rotates anticlockwise with the increasing height, which is not consistent with the common sense that the wind direction rotates clockwise with the increasing height in the northern hemisphere. Please explain why.

Coriolis forces don’t act on this scale (~1km). The forces of the topography greatly outweigh any other apparent rotational forces. The Coriolis acceleration is defined as: ***a*** *=-2* ***ω*** *×* ***v*** *,* ***a*** *≤2* ***ω*** ***v***   Where the second equation holds as the rotational vector omega and velocity vector are always perpendicular or very close to perpendicular. With omega pointing to the geometric south pole, and in the maximum acceleration case, the wind pointing true east or true west, we find the maximum possible acceleration in a clockwise direction. Earth rotates at ***ω*** *=* *2π *rad/day *24* *h* *day* *60* *min* *h* *60* *sec* *min* *=7.27⋅* *10* *-5* *rad/s* . For the maximum wind speed of ***v*** =25ms , then ***a*** *max* *=3.64⋅* *10* *-3* *m/* *s* *2* , which over the longest distance we have measured over the pass of approximately 1500m, would result in *1.5 km* *25* *m* *s* *⋅3.64⋅* *10* *-3* *m* *s* *2* *=0.28 m/s,*  of perpendicular wind speed deviation. Which equates to a rotation of *8.73 mrad=0.50°* . In fact, this rotation is independent of wind speed v, thus under no wind speeds could this deviation be greater than 0.50°. We can conclude from this that the Coriolis force is of negligible influence, and the topographic channelling forces are of much greater effect. **Addressed in the manuscript as such:** “This veering effect can not be explained by any other apparent forces on the wind such as the Coriolis force which actually act in the opposite direction. In fact, the maximum veering that can be introduced by the Coriolis effect over the Gotthard upper plateau, approximately 1500m in length, is 0.50°, independent of wind speed. This is far below the veering observed by the channelling effect created by the topography.”

1. Page 16: Turbulence kinetic energy (TKE) *k* exhibits bi-modal distributions. What is the meaning of ‘bi-modal’?

A bi-modal distribution is a probability distribution with two modes. We can separate these two distributions, with different modes, by considering northerly and southerly winds separately as they exhibit very different characteristics when considering the turbulence kinetic energy statistics. **Addressed in the manuscript as such:** “Turbulence kinetic energy (TKE) k (Equation 6),  equation 5) exhibits bi-modal distributions where northerly and southerly winds exhibit very different TKE characteristics,. This bi-model distribution is similar to the wind speed, along the LiDAR transect as shown in Figure 14”

1. Page 16, Equation 8: The authors assume that the mean wind speed is symmetrical until the second order (e.g. normal distribution). Actually, it is easy to verify whether the wind speed is normally distributed when having the raw data. Please provide the relevant verification process and statistical results.

Thank you for this very interesting suggestion. We actually refer to the *wind deviations from the mean* being normally distribution (symmetrical until the second order), but we assume that we refer to the same principle here. We verified with our 1-second resolution wind data measured by the LiDAR throughout the campaign. We observe that these deviations from the mean are actually not perfectly normally distributed, but is still symmetrical to second order. There exist other symmetric and non-skewed distribution that are symmetrical until the second order, if we fit our data with the normal distribution we observe that the tails are too heavy to create a good fit. Instead, a student-t function fit turns out to address these issues with a coefficient of *ν=2.88±.02,* *R* *2* *=0.9958, RMSE=0.0467* *m* *s* . Based on this, we can still continue with the same equation 8, where we can now determine the mean horizontal deviation *σ* *horz* *=* *ν* *ν-2* *=1.81* . Which seems to be to be independent of height. This proves that our rough upperbound still holds. **Addressed in the manuscript as such:** “The difference in power due to this non-linearity is given in 8: P_turbulence=(P(v+σ_horz )+P(v-σ_horz ))/2-P(v)=3/2 ρ A v σ_horz^2>0       (8) It should be noted that only a part of this turbulence kinetic energy can be transferred to the blades and therefore this value can only be used as an upper-bound. Additionally, this equation assumes that the wind speed deviations to the mean wind speed are symmetrical until the second order, the turbulence power can be considered only as a rough upper-bound to the influence of turbulence on the wind turbine performance. The assumption that the wind speed deviations from the mean is symmetrical to the second order can be validated by considering the 1-second wind speed data measured by the WindCube throughout the campaign. The distribution is symmetrical to the second order and can be fitted with a student-t distribution which exhibits these symmetries, with ν=2.88±0.02, R^2=0.9958.”

1. Figures 7, 9, 10, 11 are blurry, please increase the figure resolution.

We have addressed figure quality.

# Reviewer report: Reviewer response for version 1

**DOI:** 10.21956/openreseurope.20665.r50315

**Published:** 2025-02-11

**Authors:** Norman Wildmann

**Affiliations:** 1 Institut für Physik der Atmosphäre, Oberpfaffenhofen, Germany

Van Schaik et al. present an experimental study which utilizes one lidar of type Windcube v2.1 to measure wind profiles at an Alpine pass at different locations. The topic is relevant, since complex terrain flow effects are critical for wind energy siting and many uncertainties remain. I disagree with the authors that their method of sequential lidar scans was shown to be effective for site assessment in this study. The study touches a lot of interesting topics, but lacks a clear focus and in depth analyses of specific results. It is shooting very high with very general goals, but cannot convincingly show the value of the results. I recommend to reconsider the goals, restructure and resubmit a more focused study which can be backed up with the measurements that are present. General and specific comments are given below. I recommend to reject the manuscript at this point.

 General comments

 - It should be made more clear in the introduction what the actual goal is of the measurements. You could describe what is the standard procedure for WEA, how long the requirements are for instrument deployment and statistics of wind sectors t benchmark your results with. If wind resource assessment is the goal, than I get the impression that the database is much too small and the sequential lidar transects fails to provide the necessary information. If the goal is to study specific flow conditions, I would expect more of a focus on specific situations and some numerical simulations to accompany the measurements.

 - The experimental setup is flawed from my perspective because there is no reference to benchmark the results with. I think it would have been important to have at least one lidar continuously at one position. A CFD model to simulate specific wind conditions and check if matching different situations is appropriate could also be applied, but is not provided. Atmospheric stability is not measured at all, but is crucial.

 - The method section is not well introduced and the purpose for the whole wind speed matching remains very unclear when it is first introduced and is not well explained. The equations are unprecise and variable names are not well introduced.

 - The way I understand the matching algorithm, two situations are considered the same if wind vectors are within a certain margin, but that is not a full description of the flow.

 - Figures are of bad quality (low resolution). The captions are not describing the figures well.

 - Equations are not well explained and variables not described.

 - The structure of the manuscript is not consistent jumping back and forth between methods, results, definition of goals. There is no clear story-line.

 - Many statements and references are unprecise and sometimes even wrong. The authors should carefully revise the text.

 - Drawing conclusions from wind profiles that are put together from different days and conditions, just matched by wind speed at hub height can be extremely misleading.

 - The manuscript is provided in a format which is hard to review (2 columns, no line numbers, etc.). That is maybe not the authors fault, but editors should consider to change the format. 

 Specific comments:

 p.4, paragraph 1: There are clear definitions for terrain complexity in the IEC 61400. It is probably good to elude a bit on that in the introduction and make clear that the complexity in this study is of the highest complexity (I assume)

 p.4, paragraph 1: "influencing the performance of wind turbines by reducing or augmenting their efficiency". That sentence is not giving much information

 p.4, 2nd column 2nd paragraph: "airborne lidar" - that is not correct. There was no airborne lidar measuring wind transects across the valley at Perdigao.

 p.4, 2nd column 2nd paragraph: simulations cannot prove the validity of lidar measurements and were not used for that purpose in the referenced studies.

 p.4, 2nd column 3rd paragraph: WEAs operate lidars to perform WEA? that is a weird construction

 p.7, 2nd column "Lidar details": The range gate increments was already introduced in the chapter before. It may make sense to move this section before the explanation of the sequential Instrumentation overview. 

 p.9, 1st column, 2nd paragraph: why is the temperature sensor at 1m? Usually 2m is a standardized height for such measurements.

 p.9, Eq.1 etc.: At this point in the manuscript it is unclear why you want to calculate the difference of all 10-minute wind speeds for the five turbines

 p.10, Fig.6: the resolution of this figure is very low and texts are hard to read.

 p.11, right column, 2nd paragraph: pressure, temperature and humidity are not shown in this manuscript though, right? Which instrumentation is used to check it? Are there any measures for stability? How the flow develops along the transect is surely dependent on stability.

 p.11, right column, "Results": are Fig.6&7 not already results?

 p.11, right column, last paragraphs: why are these paragraphs italic? where do they belong to?

 p.13, Table 3: why not show the distributions?

 p.11, "above rated wind speed": the wind roses do not distinguish between above and below rated wind speed, the upper bin is >8m/s, but rated wind speed is 11m/s.

 p.12, Figure 8: What are turbines A-E? So far they were referenced with numbers 1-5 or T1-T5. Please be consistent or describe in the caption.

 p.13, Figure 9: The caption should be more descriptive about what the colors and arrows mean.

 p.13, right column, first paragraph: If you see a flow acceleration between the position, how can you be sure that acceleration effects are not also present at hub height and would this not be a problem for the wind matching? You actually match conditions which are very different.

 p.14, Figure 11: The figure clearly shows that for positions L4 and L8 there was strong shear and veer, thus a very different flow condition than for the other positions. How can you put these conditions together? It does not make sense.

 p.14, right column, last paragraph: I cannot believe that the shear and veer at a specific location is independent of atmospheric stability. 

 p.15, Table 4: the table shows that wind profiles of early morning, early afternoon and late afternoon are mixed.

 p.17, Fig.14: what are the axis of those TKE distribution plots? 

 p.16, "Turbulence kinetic energy": This Section is premature. You cannot make statistics of TKE with this database and calculate effects on power production the way it is done here. The references are wrong too. Reference 8 does not provide Eq. 8. I recommend to have a close look at the IEC 61400 standards.

 p.19, Fig.15: What is the normalized power production. There should be an equation somewhere.

 p.19, "Sequential vs. Synchronized": This section does not give any new information compared to the introduction and an actual comparison cannot be made, because data from synchronized lidars is not available in this campaign.

 p.19, "Conclusion": "a novel method for high-resolution, three-dimensional wind profile measurements" - I disagree. The data presented here is not "high-resolution three-dimensional" It is standard wind profiles with Windcube v2.1. for short time periods at different locations.

 p.20, second paragraph: I disagree that the matching algorithm is successful. The analyses show that just matching the wind speed at hub height is not enough to describe a common flow situation in such complex environment. Actually it is misleading to suggest it does.

Is the study design appropriate and does the work have academic merit?

No

Is the work clearly and accurately presented and does it cite the current literature?

No

If applicable, is the statistical analysis and its interpretation appropriate?

Partly

Are all the source data underlying the results available to ensure full reproducibility?

Partly

Are the conclusions drawn adequately supported by the results?

No

Are sufficient details of methods and analysis provided to allow replication by others?

Yes

Reviewer Expertise:

boundary-layer meteorology, lidar measurements, complex terrain, wind energy

I confirm that I have read this submission and believe that I have an appropriate level of expertise to state that I do not consider it to be of an acceptable scientific standard, for reasons outlined above.

# Response

**Published:** 2025-11-11

**Authors:** Brandon van Schaik

Norman Wildmann

 - It should be made more clear in the introduction what the actual goal is of the measurements. You could describe what is the standard procedure for WEA, how long the requirements are for instrument deployment and statistics of wind sectors t benchmark your results with. If wind resource assessment is the goal, than I get the impression that the database is much too small and the sequential lidar transects fails to provide the necessary information. If the goal is to study specific flow conditions, I would expect more of a focus on specific situations and some numerical simulations to accompany the measurements.

**R: **The section title of Methods was misplaced in this draft which meant that a large part of the introduction was actually displayed as the start of the introduction. We have indicated this to be changed to the correct position in the next iteration. Regarding the clarity of the goals, it is **addressed in the manuscript as such:** “This work serves as a proof of concept and as a preliminary showcase of the unique data sets and analyses that are unlocked by the sequential LiDAR experimental methodology, enabling the high-resolution study of the three-dimensional wind over the entire wind park domain.”. In response to this comment, and those made by another reviewer, we have complemented the LiDAR observations with additional CFD simulations covering the full 3-month campaign period and the entire mountain pass. These simulations provide spatially and temporally continuous wind fields that support the short-term measurements. Specifically, we have identified 8 characteristic features including, global horizontal irradiance, atmospheric stability, vertical shear magnitude, variance of the vertical turbulence, an average of the transect wind field, and the synoptic wind at 5km. To this end, the Methodology section is completely overhauled and Results updated accordingly. We believe this addresses the reviewer’s concern by providing a more complete picture of the wind conditions across all sites.

 - The experimental setup is flawed from my perspective because there is no reference to benchmark the results with. I think it would have been important to have at least one lidar continuously at one position. A CFD model to simulate specific wind conditions and check if matching different situations is appropriate could also be applied, but is not provided. Atmospheric stability is not measured at all, but is crucial.

**R: **Thanks to your suggestions we have opted to provide additional CFD simulations to help contextualize the turbine wind vector matching algorithm. We have updated the matching algorithm as elaborated in the previous comments. We refer to the Methodology section of the manuscript for the detailed overview of the 8 characteristic features that are now implemented.

 - The method section is not well introduced and the purpose for the whole wind speed matching remains very unclear when it is first introduced and is not well explained.

**R: **The equations are unprecise and variable names are not well introduced. To make this transition to the Methodology more clear, as well as to introduce the idea of the wind speed matching immediately, we have moved the LiDAR details to the instrumentation section. We have improved equations and variables and completely overhauled the Methodology section to include the 8 CFD characteristic features. We believe this overhaul has greatly improved the flow of this section.

 - The way I understand the matching algorithm, two situations are considered the same if wind vectors are within a certain margin, but that is not a full description of the flow.

**R: **We have updated the matching algorithm as elaborated in the previous comments. We refer to the Methodology section of the manuscript for the detailed overview of the 8 characteristic features that are now implemented.

 - Figures are of bad quality (low resolution). The captions are not describing the figures well. **R: **Figure quality has been addressed. We are limited to 15 words per caption by the journal, we have optimised captions within this limit.

 - Equations are not well explained and variables not described.

**R: **We have checked all equations and verified that variables are introduced and described.

 - The structure of the manuscript is not consistent jumping back and forth between methods, results, definition of goals. There is no clear story-line.

**R: **We have updated the matching algorithm as elaborated in the previous comments. We refer to the Methodology section of the manuscript for the detailed overview of the 8 characteristic features that are now implemented.

 - Many statements and references are unprecise and sometimes even wrong. The authors should carefully revise the text.

**R: **We assume said statements and references are described in the reviewer’s specific comments. We have addressed those below.

 - Drawing conclusions from wind profiles that are put together from different days and conditions, just matched by wind speed at hub height can be extremely misleading.

**R: **We have updated the matching algorithm as elaborated in the previous comments. We refer to the Methodology section of the manuscript for the detailed overview of the 8 characteristic features that are now implemented.

 - The manuscript is provided in a format which is hard to review (2 columns, no line numbers, etc.). That is maybe not the authors fault, but editors should consider to change the format. **R: **We thank the reviewer for pointing this out. The manuscript format is determined by the journal’s submission system, and unfortunately we do not have direct control over line numbering or layout. We trust that the editorial office will take this into consideration.

 Specific comments:

 p.4, paragraph 1: There are clear definitions for terrain complexity in the IEC 61400. It is probably good to elude a bit on that in the introduction and make clear that the complexity in this study is of the highest complexity (I assume)

**R: **We appreciate the reviewer’s suggestion. While the IEC 61400 provides clear definitions for terrain complexity, it is not an open-access document, and therefore we have chosen not to cite it directly. Nonetheless, we agree that the terrain in our study area qualifies as highly complex according to commonly used classifications. We have clarified this in the revised introduction to better frame the context of our work, without relying on proprietary standards. **Addressed in the manuscript as such:** “The Gotthard Wind Park can be categorised as the highest degree of complex terrain for wind energy development, characterised by large elevation changes, up- and downwind valleys and steep escarpments within the wind park itself.”

 p.4, paragraph 1: "influencing the performance of wind turbines by reducing or augmenting their efficiency". That sentence is not giving much information

**R:** **Addressed in the manuscript as such:** “influencing the performance of the wind turbine by the non-idealised wind flow into the turbine.”

 p.4, 2nd column 2nd paragraph: "airborne lidar" - that is not correct. There was no airborne lidar measuring wind transects across the valley at Perdigao.

**R: **We thank the reviewer for catching this mistake. The reference to “airborne lidar” was incorrect and has been removed. It was a confusion with the airborne surface scanning LiDAR used at Perdigão.

 p.4, 2nd column 2nd paragraph: simulations cannot prove the validity of lidar measurements and were not used for that purpose in the referenced studies.

**R:** **Addressed in the manuscript as such:** “establishing the use of LiDAR measurements as a valid wind assessment tool over a wide range of spatial resolutions.”

 p.4, 2nd column 3rd paragraph: WEAs operate lidars to perform WEA? that is a weird construction

**R: **“WEAs rarely operate more than one LiDAR to perform a wind energy assessment.” **Replaced by:** “WEA studies rarely operate more than one LiDAR”

 p.7, 2nd column "Lidar details": The range gate increments was already introduced in the chapter before. It may make sense to move this section before the explanation of the sequential Instrumentation overview. 

**R: **Thank you for the helpful suggestion, we have adopted this in the revision.

 p.9, 1st column, 2nd paragraph: why is the temperature sensor at 1m? Usually 2m is a standardized height for such measurements.

**R: **Out of practical reasons this was done. A one metre rebar pole could be easily hammered into the soil. Longer poles to mount the sensor at 2m would require guywires which was not always possible in the terrain. Since the LiDAR was moved every week, this was not practical. Since the surface topography is free of brushes and rocks, this difference should not impact the variances in the temperature readings much. Particularly since we average over 10min intervals. Since the temperature is only used for air density calculations in which the temperature only has a small sensitivity, we do not see a reason to distrust the accuracy of this data. **Addressed in the manuscript as such:** “A radiation-shielded combined pressure, temperature, and humidity sensor was placed near the LiDAR 1 m above the ground which is solely used to calculate the local air density.”

 p.9, Eq.1 etc.: At this point in the manuscript it is unclear why you want to calculate the difference of all 10-minute wind speeds for the five turbines

**R: **With the additional CFD simulations methods in the manuscript, this has been addressed accordingly. We have also introduced the goals more clearly, and earlier in the manuscript.

 p.10, Fig.6: the resolution of this figure is very low and texts are hard to read.

**R: **All figures have been replaced with higher-resolution versions in the revised manuscript. In addition, Figure 6 is no longer included, as it became irrelevant following the revisions to the Methodology section and has been removed.

 p.11, right column, 2nd paragraph: pressure, temperature and humidity are not shown in this manuscript though, right? Which instrumentation is used to check it? Are there any measures for stability? How the flow develops along the transect is surely dependent on stability.

**R: **We only use pressure temperature and humidity to calculate the average air density over the whole campaign. We mention the existence of this sensor as it is included in the data set that is co-published with this paper. The sensor in use is the standard PTH sensor provided by VAISALA for the WindCube v2.1. More details are now given in the instrumentation table (Table 1). Atmospheric stability is now addressed with CFD simulations. We refer the reviewer to our reply of his first comment as well as the Methodology section of the revised manuscript for the full details.

 p.11, right column, "Results": are Fig.6&7 not already results?

**R: **These figure have become irrelevant following the revisions to the Methodology section and have since been removed.

 p.11, right column, last paragraphs: why are these paragraphs italic? where do they belong to?

**R: **We thank the reviewer for noting this editing error. The paragraphs are now placed correctly in the revised manuscript.

 p.13, Table 3: why not show the distributions?

**R: **In our view, the distributions are not directly relevant to the goals of the study. Presenting the results in tabular form provides a more space-efficient and quantitative summary, allowing readers to readily compare the two wind directions as well as reproduce/refer to the data immediately.

 p.11, "above rated wind speed": the wind roses do not distinguish between above and below rated wind speed, the upper bin is >8m/s, but rated wind speed is 11m/s.

**R: **Excellent suggestion, the wind roses are updated to reflect the different stages in the E-92 power curve, and the figure has been updated.

 p.12, Figure 8: What are turbines A-E? So far they were referenced with numbers 1-5 or T1-T5. Please be consistent or describe in the caption.

**R: **Combined with the previous comment, we have adapted this as well in the figure.

 p.13, Figure 9: The caption should be more descriptive about what the colors and arrows mean.

**R: **We are limited to 15 words per figure/table caption by the journal regulations. We have described this in more detail in the text. We have also added a legend to explain the difference in colours. Besides, this figure has been replaced due to the improved methodology as suggested in your earlier comments. We refer the reviewer to the Transect wind profile evolution and Wind shear and veer subsections in the results for our improved results.

 p.13, right column, first paragraph: If you see a flow acceleration between the position, how can you be sure that acceleration effects are not also present at hub height and would this not be a problem for the wind matching? You actually match conditions which are very different. **R: **We have updated the matching algorithm as elaborated in the previous comments. We refer to the Methodology section of the manuscript for the detailed overview of the 8 characteristic features that are now implemented. This flow acceleration can now be identified using by CFD simulation characteristic features: “Vertical shear magnitude”, “Variance of the vertical turbulence”, and “Transect-averaged wind”. We refer the reviewer to the Transect wind profile evolution and Wind shear and veer subsections in the results for our improved results.

 p.14, Figure 11: The figure clearly shows that for positions L4 and L8 there was strong shear and veer, thus a very different flow condition than for the other positions. How can you put these conditions together? It does not make sense.

**R: **We have updated the matching algorithm as elaborated in the previous comments. We refer to the Methodology section of the manuscript for the detailed overview of the 8 characteristic features that are now implemented. This shear/veer concern can now be identified using by CFD simulation characteristic features: “Vertical shear magnitude”, “Variance of the vertical turbulence”, “Global Horizontal Irradiance”, “Atmospheric stability”, and “Synoptic wind at 5km”. We refer the reviewer to the Transect wind profile evolution and Wind shear and veer subsections in the results for our improved results.

 p.14, right column, last paragraph: I cannot believe that the shear and veer at a specific location is independent of atmospheric stability. 

**R: **We have updated the matching algorithm as elaborated in the previous comments. We refer to the Methodology section of the manuscript for the detailed overview of the 8 characteristic features that are now implemented. This shear/veer and atmospheric stability concern can now be identified using by CFD simulation characteristic features: “Vertical shear magnitude”, “Variance of the vertical turbulence”, and “Atmospheric stability”. We refer the reviewer to the Transect wind profile evolution and Wind shear and veer subsections in the results for our improved results.

 p.15, Table 4: the table shows that wind profiles of early morning, early afternoon and late afternoon are mixed.

**R: **We have updated the matching algorithm as elaborated in the previous comments. We refer to the Methodology section of the manuscript for the detailed overview of the 8 characteristic features that are now implemented. This time of day mixing can now be identified using by CFD simulation characteristic features: “Global Horizontal Irradiance”, and “Atmospheric Stability” We have consequently updated the table to address the stricter matching protocol discussed in the overhauled Methodology section.

 p.17, Fig.14: what are the axis of those TKE distribution plots? 

**R: **We have revised the captions to clarify that the plots represent probability density functions of the TKE. The as the exact values of TKE are not directly relevant for our conclusions, and for clarity we therefore choose to not include x ticks or y ticks. Since our caption size is limited, we have updated the manuscript section in which the figure is first referred to as shown below. The plots are intended to highlight relative differences, and since all TKE distributions share identical x-limits, they can be visually intercompared as discussed in the manuscript. **Addressed in the manuscript as such:** “This bi-model distribution is similar to the wind speed, along the LiDAR transect as shown in Figure 12. In these figures, the red lines represent the probability density functions of the TKE, while the blue vertical lines indicate the mean TKE, allowing for a visual identification of where turbulence is strongest along the transect.”

 p.16, "Turbulence kinetic energy": This Section is premature. You cannot make statistics of TKE with this database and calculate effects on power production the way it is done here. The references are wrong too. Reference 8 does not provide Eq. 8. I recommend to have a close look at the IEC 61400 standards.

**R: **We thank the reviewer for this valuable comment. We have revised the section to clarify that our analysis is not intended as a full turbulence kinetic energy (TKE) assessment, but rather as a simplified approach to provide a rough upper bound on the potential influence of turbulence on turbine performance. We now emphasize that Eq. (8) is a first-order approximation based on the curvature of the power curve and assumes symmetric fluctuations up to second order. Furthermore, we have verified the applicability of Eq. (8) using our 1-second LiDAR data, which shows that the deviations from the mean are approximately symmetric and can be fitted by a Student-t distribution. This supports the use of Eq. (8) as a conservative upper-bound rather than a precise quantification. Finally, we have corrected the references and added a note pointing the reader to the IEC 61400 standards, which provide the proper framework for turbulence intensity and load assessments. **Addressed in the manuscript as such:** “Equation 8 provides a first-order estimate of how turbulence around the mean affect turbine power output: Equation 8 *P* *turbulence*= *P* *v*+ *σ* *h* *orz*+ *P* *v*- *σ* *h* *orz* *2*- *P* *v*= *3* *2* *ρ* *A* *v* *σ* *h* *orz* *2* *>0*   However, the actual effect of turbulence is governed by the curvature of the turbine power curve: In convex sections of the power curve, positive and negative fluctuations are weighted asymmetrically, leading to a net increase in power relative to the mean wind speed. Whereas in concave sections, the reverse occurs and fluctuations produce a net decrease in power. Therefore Equation 8 represents only a rough upper-bound to the influence of turbulence on power production. It does not account for the changing curvature of the real power curve, nor for higher-order asymmetries in the wind speed distribution. For a full quantification, we refer the reader to a work that integrates the measured wind speed probability density function and other vertical variations with the actual turbine power curve as referred to in international standards. Furthermore, we have validated the assumption of second-order symmetry in the wind speed variations measured the Doppler wind LiDAR. The 1-second wind speed data from the WindCube during the campaign shows that the deviations from the mean are not perfectly normally distributed. They rather remain symmetric up to second order and an be fitted optimally with a Student-t distribution ( *ν*=2.88±0.02, *R*2=0.996 ), which captures the heavy tails observed. From this the mean horizontal deviation can be expressed as *σ* *horz*= *ν*/( *ν*-2) =1.81 , Which seems to be independent of height. Which consequently validates that the rough upper-bound still holds.”

 p.19, Fig.15: What is the normalized power production. There should be an equation somewhere.

**R:** **Addressed in the manuscript as such:** “The power production is normalised to 7 ms^(-1) 7 m/s by the cubic relation between power and wind speed in this range with Equation 9. Equation 9 *P* *normalised* *=P* *v* *C* *p* *(7m* *s* *-1* *)* *7 m* *s* *-1* *3* *C* *p* *(v)* *v* *3* *, 4 m* *s* *-1* *≤v≤10 m* *s* *-1*  “

 p.19, "Sequential vs. Synchronized": This section does not give any new information compared to the introduction and an actual comparison cannot be made, because data from synchronized lidars is not available in this campaign.

**R: **We have conducted additional CFD simulations to address this concern, as outlined in our earlier responses. We believe that these methodological adaptations improve the robustness of the results and provide sufficient support for our conclusions in this section.

 p.19, "Conclusion": "a novel method for high-resolution, three-dimensional wind profile measurements" - I disagree. The data presented here is not "high-resolution three-dimensional" It is standard wind profiles with Windcube v2.1. for short time periods at different locations.

**R: **We thank the reviewer for this comment. While we agree that standard WindCube v2.1 instruments provide vertical wind profiles at single locations, our methodology goes beyond this typical use. By deploying the lidar sequentially along a dense transect with 150 m horizontal spacing and 10–20 m vertical resolution, we obtain a high-resolution, three-dimensional reconstruction of the wind field across complex terrain. Something that cannot be achieved with a single lidar in a fixed location. Furthermore, the newly added CFD simulations now support and address the reviewer’s concerns about temporal discontinuity and the matching algorithm. Therefore, we believe that the description of the method as "high-resolution, three-dimensional wind profile measurements" is appropriate in the context of this study.

 p.20, second paragraph: I disagree that the matching algorithm is successful. The analyses show that just matching the wind speed at hub height is not enough to describe a common flow situation in such complex environment. Actually it is misleading to suggest it does.

**R: **The newly added CFD simulations help address the reviewer’s concerns regarding temporal discontinuity and provide validation for the adapted matching algorithm. We therefore consider the revised algorithm to be appropriate and effective for the purposes of this study.
